# Prediction of Bioactive Metabolites from American *Aconitum* Using Network Integrating Cellular Morphological Profiling and Mass Spectrometry Data

**DOI:** 10.1021/acs.jnatprod.4c01458

**Published:** 2025-07-11

**Authors:** Yi Zhao, Dennis Y. Liu, Trevor N. Clark, Roger G. Linington, Edward J. Kennelly

**Affiliations:** † Department of Biological Sciences, Lehman College, The City University of New York, 250 Bedford Park Boulevard West, Bronx, New York 10468, United States; ‡ Biology Ph.D. Program, The Graduate Center, The City University of New York, 365 Fifth Avenue, New York, New York 10016, United States; § Department of Chemistry, 1763 Simon Fraser University, 8888 University Drive, Burnaby, British Columbia V5A 1S6, Canada

## Abstract

Asian and American *Aconitum* species are phylogenetically close, but only certain Asian species have been well-studied for their medicinal properties. This study aims to discover bioactive compounds in two American *Aconitum* species based on a systematic networking strategy integrating both mass spectrometry data and biological profiles from a high-throughput phenotypic screening assay, Cell Painting. The chemical profiles of four different plant parts of two American *Aconitum* species (*A. columbianum* and *A. uncinatum*) were obtained by ion mobility mass spectrometry and compared with two Asian (*A. carmichaelii* and *A. fischeri*), and one European species (*A. napellus*). Biological screening, image analysis, and feature extraction were performed on *Aconitum* extracts using the Cell Painting assay. The results provided 2,090 unique morphological features per extract, which were further reduced to 429. In conjunction with 4,400 chemicals from a library with known mechanisms of action, 198 unique hierarchical clusters were established. An overall activity heuristic called CP score was calculated for each sample. After integrating the CP score and spectrometric data, a network filtered for higher CP scores was constructed and the compounds with high activity were tentatively identified. The network contained mostly American *Aconitum* species, suggesting that these understudied plants produce useful bioactive compounds.

## Graphical abstract

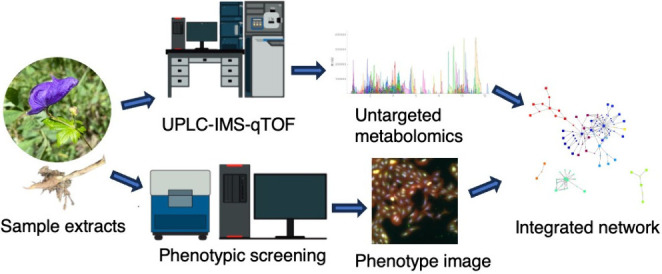

Roots of *Aconitum* have been used as a traditional medicine in Asian countries for thousands of years to treat bronchial asthma, diarrhea, edema, fainting, gastroenteritis, pain, rheumatoid arthritis, and other conditions.^1,2^ These therapeutic effects are mainly attributed to their bioactive diterpenoid alkaloids.^3^ Compared with the large number of studies on Asian *Aconitum*, there are few records about how American *Aconitum* has been used medicinally.^4^ However, the molecular phylogeny using international transcribed spacer sequencing results of *Aconitum* suggest that American *Aconitum* are more closely related to important medicinal Chinese species, especially two listed in the *Chinese Pharmacopeia*, than to other Asian species and European species.^5,6^ Since diterpenoid alkaloids can serve as chemotaxonomic markers in *Aconitum*,^7–9^ we hypothesize that American *Aconitum* also contain bioactive diterpenoid alkaloids and could be potential sources for novel drugs. This study aims to analyze the chemical profile of American *Aconitum*, explore the medical potential using a high-throughput screening method, and target bioactive compounds in *Aconitum* with cellular mechanisms of action (MOAs).

The chemical profiles were obtained by ultra-performance liquid chromatography quadrupole-traveling wave ion mobility-time-of-flight mass spectrometry (UPLC-q-TWIM-TOF-MS). Ion mobility spectrometry (IMS) is a powerful technique that can even separate isomers based on their conformations through differential mobility. Traveling wave ion mobility (TWIM) coupled with time-of-flight (TOF) mass spectrometry allows this separation on the time scale of standard UPLC-based chromatography.^10^ This technique is useful in providing high-resolution untargeted detection of natural products in *Aconitum*.

The potential effects on cells and the MOAs of these *Aconitum* extracts were tested using a high-content screening assay, Cell Painting. Cell Painting is an unbiased, image-based cellular phenotype screening assay, allowing for extensive, quantitative, and high-throughput morphological profiling and systematic measurement of cellular response to chemical or genetic perturbation.^11,12^ The assay involves staining human osteosarcoma U2OS cells with five fluorescent dyes that target cellular DNA, RNA, actin, Golgi apparatus, plasma membrane, endoplasmic reticulum, and mitochondria. After cell perturbation with plant extracts and the addition of fluorescent dyes, morphological features were extracted by imagining internal cellular structures using a high-content microscope.^11^ By analyzing changes to morphological features induced by natural product extracts and comparing these profiles to those of library compounds with known MOAs, the assay helps to predict molecular MOAs at the cellular level and has proven useful in new drug discovery.^13^ Cell Painting is a highly standardized phenotypic assay that demonstrates robust reproducibility across different batches of plates, different operators, and different institutions.^14^ It has been applied toward the classification of dozens of unique MOAs across multiple small molecule libraries.^15^

A network integrating both high-throughput cell phenotype screening results and untargeted mass spectrometry data was established on the open-access data integration platform NP Analyst.^16^ This bioactivity-based network directly predicts bioactive constituents by calculating the strength and consistency of the activity profiles for each mass spectrometric feature in complex mixtures. After setting up cutoff values for both the activity strength value and consistency score, the network only retains the mass spectrometric features that are Cell Painting active. By focusing on “high activity score” compounds, the predicted bioactive compounds were identified on the network.

## Results and Discussion

A total of 58 individual *Aconitum* plants from five different species (Table S1) were analyzed, including two American (*A. columbianum* Nutt. and *A. uncinatum* L.), two Asian (*A. carmichaelii* Debeaux and *A. fischeri* Rchb.), and one European species (*A. napellus* L.), and these species have been abbreviated as ACo, AU, ACa, AF, and AN, respectively, in the figures. Different parts of each plant, including flowers, leaves, roots, and stems, were extracted and analyzed by LC-MS and Cell Painting.

### Cell Painting Assay Analysis

Biological screening, image analysis, and feature extraction were performed on extracts assayed in four different concentrations in Cell Painting, each with 2,090 unique morphological features measured per extract. To explore the morphological consequence of extract treatment on U2OS cells, hierarchical clustering was performed on this data set in conjunction with the TargetMol library of 4,400 compounds with known MOAs. Prior to hierarchical clustering, the combined data set (6,088 samples × 2,090 features; including controls) was subjected to feature reduction and activity filtering using the following principles: first, a feature selection strategy known as fast correlation-based filtering^17^ was employed to reduce the total number of features to 429 by eliminating invariant and redundant features; and second, an overall activity heuristic called the CP score was calculated for each sample with those samples at or below the CP score of the vehicle removed from analysis (2,240 samples × 429 features remaining). Cell Painting features were first converted from cell-by-cell values to normalized well-by-well values using the histogram-difference (HisDiff) algorithm.^18^ CP scores, which provide a single metric to assess the overall strength of the phenotype, were calculated as the sum of the squares of the HisDiff values for each feature.

Hierarchical clustering of the resulting data set produced 198 unique clusters as gated by contiguous clustering of positive control, latrunculin B, an actin polymerization inhibitor.^19^ Selected clusters containing all *Aconitum* extracts above the CP score activity cutoff, as well as the positive control latrunculin B, are shown in Figure 1. The full hierarchy of 198 clusters is shown in the Supporting Information (Figure S1). Twenty-four of the 388 plant extract samples have a higher CP score than the vehicle control, and most of these extracts are from wildcrafted American *Aconitum* species. Merged, false color images containing all imaged channels for each clustered control, TargetMol compound, and *Aconitum* extracts (Figure 2) are located within Clusters 40, 42, 120, 135, 185, and 198. Hierarchical clustering heatmaps (Figure 3) for the aforementioned clusters include selected size and shape features for treated cells, normalized against the comparable values from negative control wells, with yellow cells denoting values that are higher than control values and blue cells denoting values that are lower than control values. A high-resolution scalable vector version of this graph is in the Supporting Information (Figure S2). Within each cluster, samples generally exhibit strong uniformity in terms of the features being perturbed and the vector of that perturbation (either positive or negative changes compared with the control).

**Figure 1:**
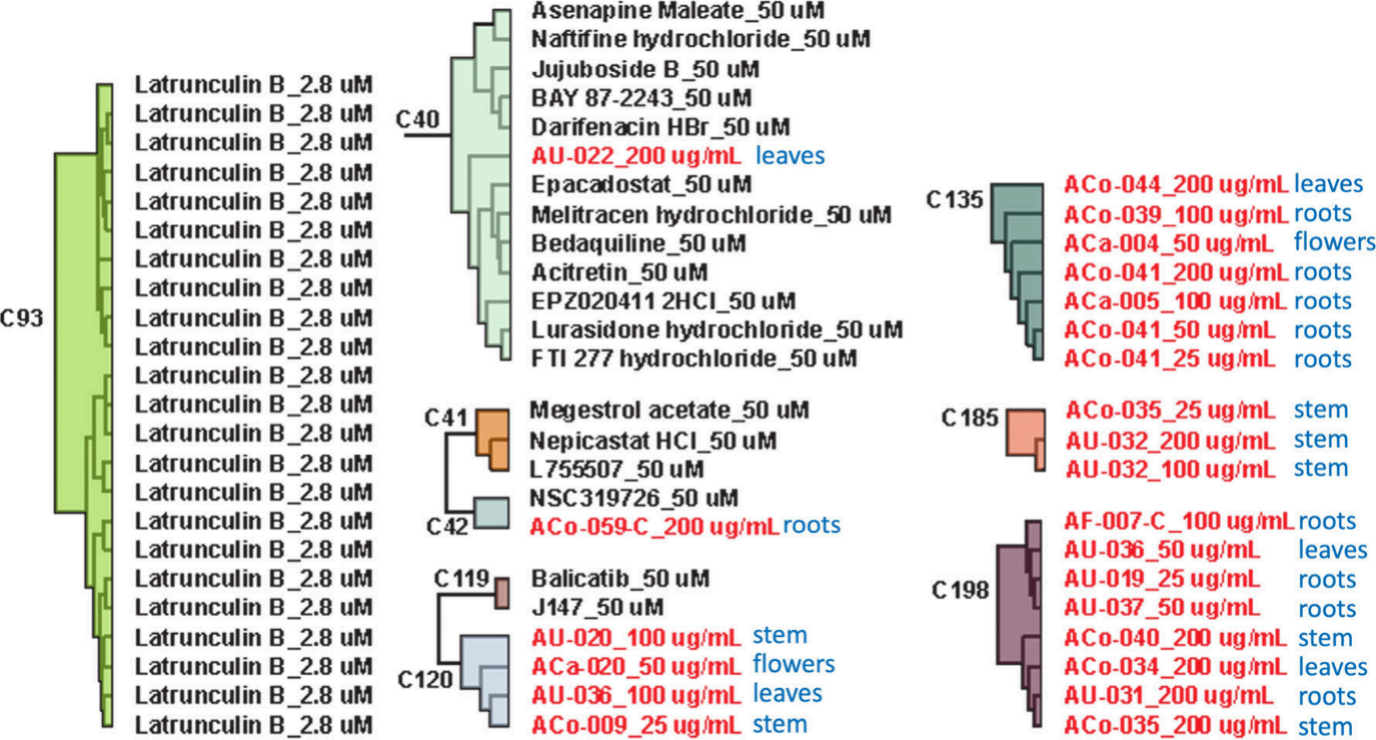
Select hierarchical clusters of *Aconitum* samples, control compounds, and members of the TargetMol library. Cluster 93 shows positive control latrunculin B as a frame of reference for establishing clustering thresholds. Samples denoted in black text are either positive controls or compounds from the TargetMol library. Samples in the red text are *Aconitum* extracts.

**Figure 2:**
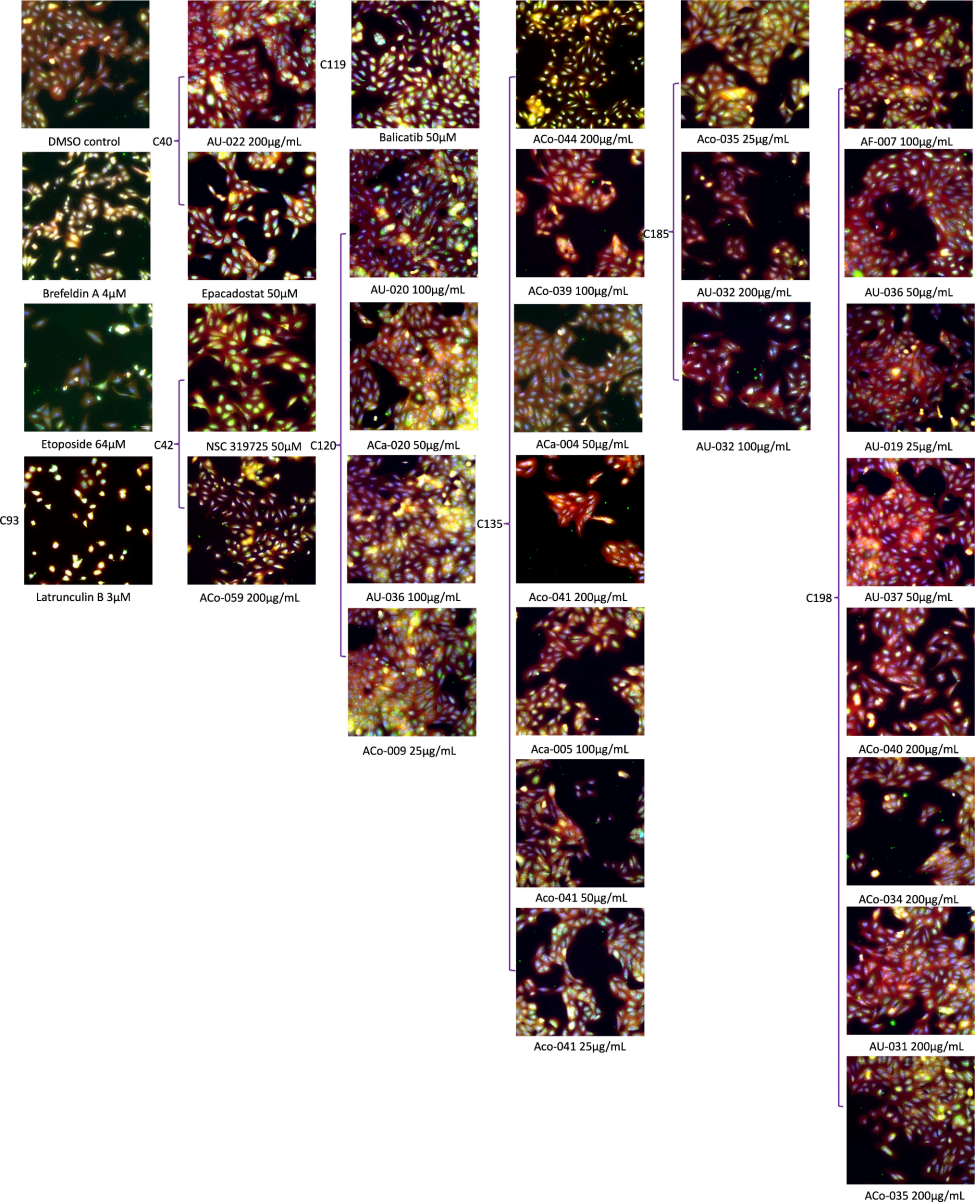
Composite fluorescent images of stained U2OS cells treated with *Aconitum* extracts and controls. False coloring legend: blue, Hoechst 33342 (DNA); green, Fluor 488-Concanavalin A (endoplasmic reticulum); yellow, PhenoVue 512 (RNA); cyan, PhenoVue 641 (mitochondria); red, Fluor 568-Phalloidin (actin) and Fluor 555-WGA (Golgi and plasma membrane). Cluster numbers of each sample are shown at the left side of the image.

**Figure 3:**
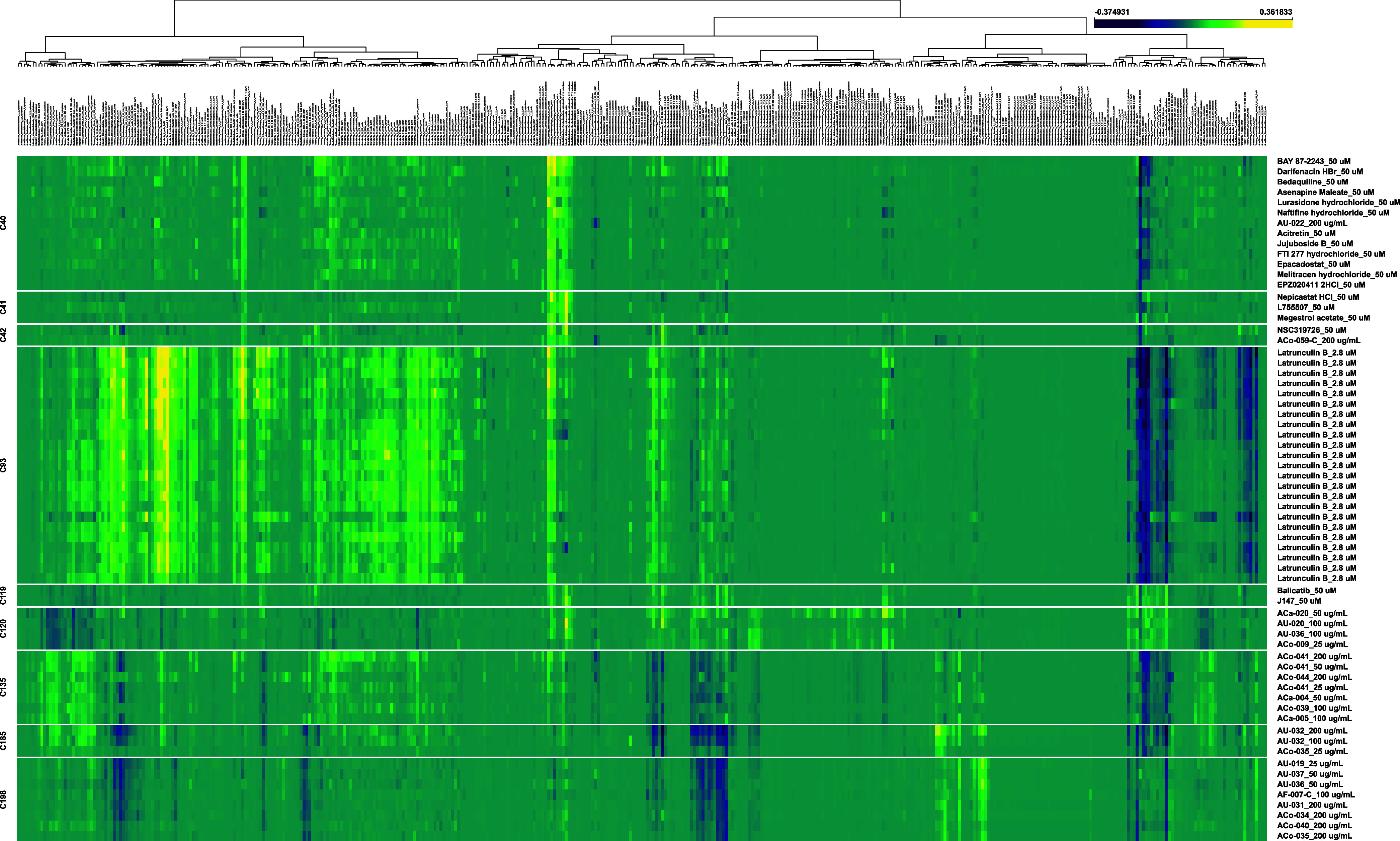
Cell Painting heatmap of select *Aconitum*, control, and TargetMol library clusters. Vertical axis shows Cell Painting profiles for all clusters containing *Aconitum* samples and positive control latrunculin B. The horizontal axis shows hierarchical clustering of a reduced subset of 429 features. Yellow shading indicates increasing HistDiff values compared to vehicle control while deep blue shading indicates decreasing HistDiff values compared to vehicle control.

Compounds with similar MOAs result in similar phenotypic profiles in the Cell Painting assay.^20^ Conversely, similar phenotypic profiles may be caused by compounds with similar MOAs. Examination of each cluster in Figure 1 may help to uncover the MOAs of *Aconitum* extracts. Table S2 shows the target pathway and compound class for each TargetMol compound, as reported by the manufacturer. Clusters 40, 41, 42, 119, and 120 contain close clustering between *Aconitum* extracts and TargetMol compounds. Clusters 119 and 120 are adjacent, and Cluster 119 contains two compounds from the TargetMol library: balicatib and J147. Balicatib is an inhibitor of osteoclastic enzyme cathepsin K, which is an important target for osteoporosis treatment.^21^ By inhibiting cathepsin K, balicatib could cause actin-bundling,^22^ as well as causing an increase in the cellular lysosomal concentration,^23^ which may be observed in Figure 2. J147 is a neurotrophic and neuroprotective compound that can modulate the adenosine monophosphate-activated protein kinase/mammalian target-of-rapamycin (AMPK/mTOR) pathway and inhibits mitochondrial α-F1-ATP synthase. Comparing the heatmap with Cluster 119 (Figure 3), *Aconitum* extracts in Cluster 120 appear to also enhance cell growth with an increased number of cells and higher values of organelle feature measurements. Cluster 120 only includes *Aconitum* extracts from the aerial parts, which is known to have lower content of alkaloids than roots.^24^ The chromatograms of different parts of American *Aconitum* are shown in Figure S3. The active compounds in Cluster 120 are more likely determined by nonalkaloidal constituents, such as flavonoid glycosides, phenylpropanoids, steroids, and polysaccharides.^25^ These nonalkaloidal constitutes in *Aconitum*, therefore, warrant further investigation.

Clusters 41 and 42 are adjacent and share similar Cell Painting profiles. *A. columbianum* root extract is the only *Aconitum* extract in these two clusters. The pharmacological effect of *Aconitum* is most closely associated with diterpenoid alkaloids, and roots have higher concentrations of diterpenoid alkaloids as compared to the arial parts.^24^ Figure 4 shows that the root constituents in American *Aconitum* roots are chemically different from non-American species, suggesting that constituents found in *A. columbianum* merit further investigation for their bioactive properties. The TargetMol compound NSC319726, a potent cancer cell growth inhibitor (Table S2), clusters with *A. columbianum* root extract, indicating that diterpenoid alkaloids in *A. columbianum* may also impact cancer cell development.

**Figure 4:**
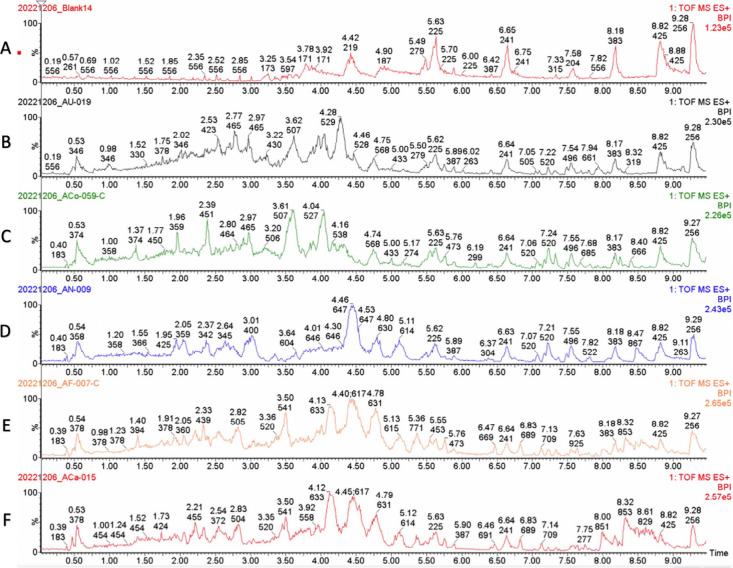
Typical UPLC-MS chromatograms of root samples from five *Aconitum* species. (A) blank; (B) *A. uncinatum*; (C) *A. columbianum*; (D) *A. napellus*; (E) *A. fischeri*; (F) *A. carmichaelii*.

Cluster 41 contains three TargetMol compounds: megestrol acetate, nepicastat HCl, and L755507. Megestrol acetate, a synthetic derivative of a steroid hormone, is used clinically in hormonally relative cancers.^26^ L755507 can inhibit c-Myc heterodimerization and induce cancer cells apoptosis.^27^ Since some *Aconitum* diterpenoid alkaloids have reported anticancer activities,^28^ they may account for these CP phenotypic effects in Cluster 41.

Cluster 40 has only one *A. uncinatum* leaf extract (AU-022) with 12 TargetMol compounds presenting a diverse spectrum of pharmacology (Table S2). The mass spectrum of AU-022 showed only minor differences compared to those of other *A. uncinatum* leaf samples. Progenesis QI was used to analyze mass spectrometry data, and the alignment of chemical features (retention time, accurate mass, and mass intensity) among different injections was made to one target injection to compensate for drifts in retention time between runs. The alignment score of AU-022, which is calculated to measure the alignment quality in Progenesis QI, was 74.3% and comparable with other samples. Principal component analysis (PCA) also confirmed that AU-022 is not chemically distinct from other *A. uncinatum* leaf samples (Figure S4). However, when we performed orthogonal projections to latent structure discriminant analysis (OPLS-DA) of *A. uncinatum* leaf samples (Figure S5), the chemical features from *A. uncinatum* leaf samples vary from each other. This would explain why leaf extracts from different individuals of *A. uncinatum* did not show a consistent clustering distribution (Figure 1), which dispersed in Clusters 40, 120, and 198, even though they were collected from the same population at the same time (Table S1). The phenotypic features induced by AU-022 and other *A. uncinatum* leaf extracts are not uniform on the heatmap (Figure 3). Leaf chemistry is complex and can vary significantly under different environmental conditions, such as light and water.^29^ In contrast to *A. columbianum* root extracts, which all grouped in Cluster 135, and *A. uncinatum* root extracts, which all grouped in Cluster 198, leaf extracts are more widely distributed (Figure 1) due to greater variation in their chemical feature expression.

Clusters 135, 185, and 198 do not contain known controls and are exclusively composed of *Aconitum* extracts with unique profiles. The CP assay relies on having a comprehensive library that covers a wide range of MOAs that have been well validated. However, many compounds in the TargetMol are assigned MOAs based on the limited literature data that is available, and this is especially challenging for compounds with limited mechanistic studies. We have found that TargetMol tends to have more drugs that are antiproliferative inhibitors and fewer modulators of cell health. The Cell Painting assay is a tool for predicting MOA rather than definitively assigning MOA. The greater the overlap in compounds sharing a defined MOA with the target sample, the greater is the probability that the sample also shares that MOA. Clusters 135, 185, and 198 that do not contain any controls may be driven by a single, or some combination of, common metabolites that are not reflected in the TargetMol library or were not assayed at their critical concentration. However, both Clusters 135 and 185 contain duplicate extracts but at different concentrations, supporting their respective clustering parameters. This effect is commonly observed in multiparametric image-based screening because biological fingerprints vary with concentration, leading to fragmentation of clustering for samples with the same MOA at different testing concentrations. Finally, Cluster 185 is composed of only stems from American *Aconitum*. The two different concentrations of AU-032 in Cluster 185 exhibit the highest CP scores among all the extracts, 0.92 for 200 μg/mL and 0.57 for 100 μg/mL. This indicates that chemical features uniquely found in the stems of American *Aconitum* may contribute to the Cell Painting activities and unique MOAs on cells and thus warrant further investigation.

### Chemical Profile Results

The chemical profiles of all extracts were obtained using UPLC-IMS-qTOF in positive mode. Sample chromatograms are shown in Figure 4. Diterpenoid alkaloids, important *Aconitum* markers, are responsible for much of their reported bioactivities and/or toxicities.^30^ Since the concentration of diterpenoid alkaloids in roots is much higher than that in aerial parts, Figure 4 shows the root extracts from different species. The chemical chromatograms of each plant sample were further analyzed with MS2Analyte and Progenesis QI. Most of these diterpenoid alkaloids eluted from 2 to 6 min based on their polarity, mass range, and fragmentation. In addition, ten diterpenoid alkaloid standards we coinjected, including aconitine, benzyolaconine, benzolhypaconine, benzoylmesaconine, fuziline, hypaconitine, lappaconitine, mesaconitine, neoline, and talasamine. They have similar chemical skeletons,^28^ making the identification of each compound challenging. A total of 17,439 chemical features were found using Progenesis QI of which 5,705 were tentatively identified with the help of an in-house *Aconitum* database. All of these chemical features, identified or unidentified, were imported into NP Analyst with CP score results to generate the network to target active compounds in *Aconitum*. Principal component analysis (PCA) was performed on all plant samples of different species (Figure 5). The results showed that the two American species, two Asian species, and one European species clustered, respectively. Two Asian species and the European species are close to each other, implying their chemical similarity. American species are distinct from non-American species, indicating that they are more chemically different from non-American species.

**Figure 5:**
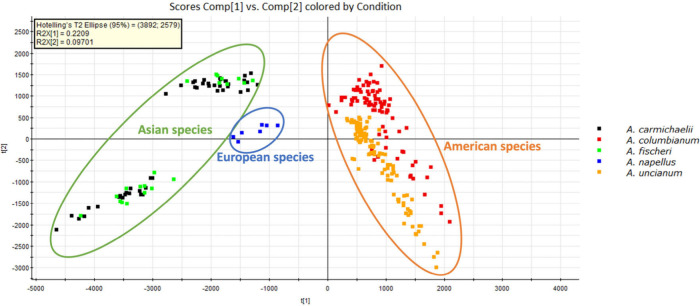
PCA of 106 *Aconitum* extracts from five color-coded species. Each square represents one injection, and each extract was injected three times.

### Network Construction

A network (Figure 6) integrating both mass spectrometry features (retention time, accurate mass, and mass intensity) and Cell Painting activity was created by using a minimum cluster score of 0.5 and a minimum activity score of 0.03, revealing strong correlations between chemical features and the Cell Painting activity. The cluster score (a measure of phenotype similarity) of 0.5 is high enough to require close alignment of the phenotypes between samples, while the 0.03 activity score (a measure of phenotype strength) is a low filter that requires activities to be stronger than background noise but is inclusive of selective and weaker phenotypes.^16^ The samples with bioactive chemical features above the selected scores are shown in the network in Figure 6. Each square represents one sample, and each circle represents a chemical feature; the circle radius describes the activity score (a larger radius = a higher activity score). The same color samples and chemical features in the network indicate that they belong to the same community. These communities share high interconnectivity between sample nodes, meaning that samples in each community are connected by bioactive MS features. From the network, it was observed that most of the American *Aconitum* species samples contained features that were above the default cluster score of 0.5, as well as activity score of 0.03. For example, AU_032, AU_035, AU_049, and AU_052 were found at the top left in Community 7 (Figure 6). ACo_031, ACo_038, ACo_039, and ACo_040 were found in the bottom right corner in a light green color (Community 8). These AU and ACo are the samples from American *Aconitum*. Few non-American *Aconitum* were found in Figure 6, only one or two ACa, AF, and AN squares, which represent non-American *Aconitum* samples can be observed in the middle of this figure.

**Figure 6:**
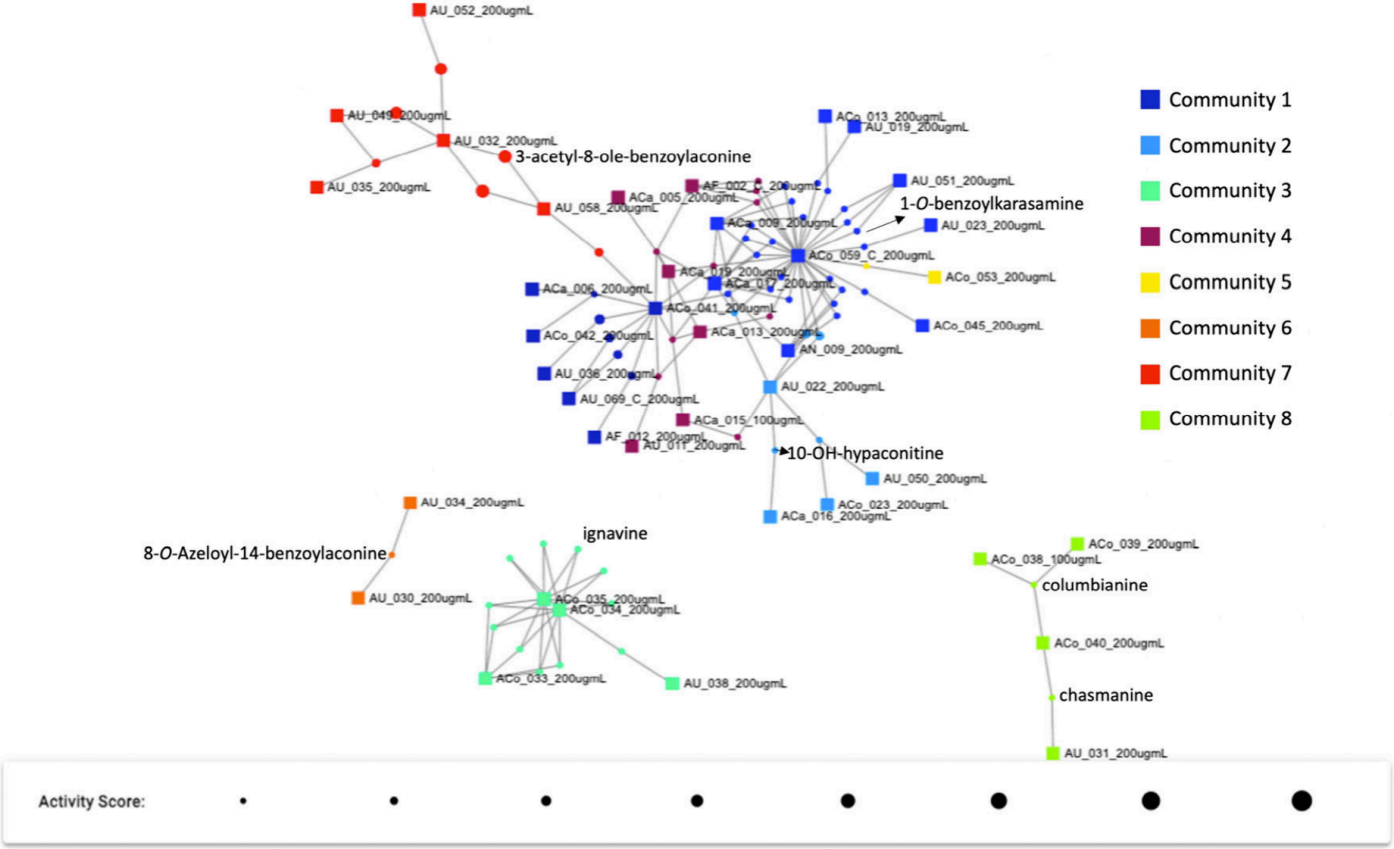
NP Analyst network integrating both mass spectrometry data and Cell Painting activity profiles. Each square represents one sample, and each circle represents a chemical feature. The size of the circle represents the activity score. The same color of the network indicates that they belong to the same community, sharing a higher interconnection.

There have been few chemical and biological publications reporting on the American *Aconitum* species. American *Aconitum* are phylogenetically close to medicinally important Chinese *Aconitum* species,^6^ and our study supports the hypothesis that American *Aconitum* also contain bioactive constituents. This model, integrating qToF-IMS data with Cell Painting activity, demonstrates how chemical features present in botanical extracts can be potentially correlated to bioactivity using the CP assay. To identify the chemical features that contribute to the Cell Painting activity, Progenesis QI, an informatics platform, and an in-house chemical library have been used.

### Active Compound Identification

From the NP Analyst network (Figure 6), each circle represents one chemical feature with a CP > 0.03, and these active chemical features are precursor adducts from the active compounds targeted from the network. The identification of chemical features in the network was aided by Progenesis QI. In addition, an in-house *Aconitum* database containing 1,166 compounds was established from SciFinder, including scientific name, structure formula, neutral mass, and CAS number. Using both the in-house database and Progenesis MetaScope, we tentatively identified chemical features. These chemical features and their adducts were identified based on exact mass, fragmentation ions, compound polarity, and other properties, but further confirmation of these structures is necessary.

Seven diterpenoid alkaloids (Figure 7) have been tentatively identified from the network. Columbianine (**1**) and ignavine (**2**) were only detected from *A. columbianum*; chasmanine (**3**) and 3-acetyl-8-ole-benzoylaconine (**6**) were only identified from *A. uncinatum*; both are American species (Table 1). These four markers may contribute to the observed biological effects in the Cell Painting assay (Figure 1). Compound **1** is a nonester diterpenoid alkaloid (NDA), and **2** is a monoester diterpenoid (MDA). Since NDAs and MDAs are known to be less toxic than the diester diterpenoid alkaloids (DDAs) in *A. carmichaelii*, they may warrant further investigation into their biological activity. Ignavine (**2**) augmented the μ opioid receptor agonist at a low concentration and inhibited it at a high concentration.^31^ Markers **3** and **6** are both DDAs, but **6** has a 0.23 activity score, which is 3–5 times higher than other markers; however, it is not clear if the activity is related to any potential toxicity associated with DDAs.

**Figure 7:**
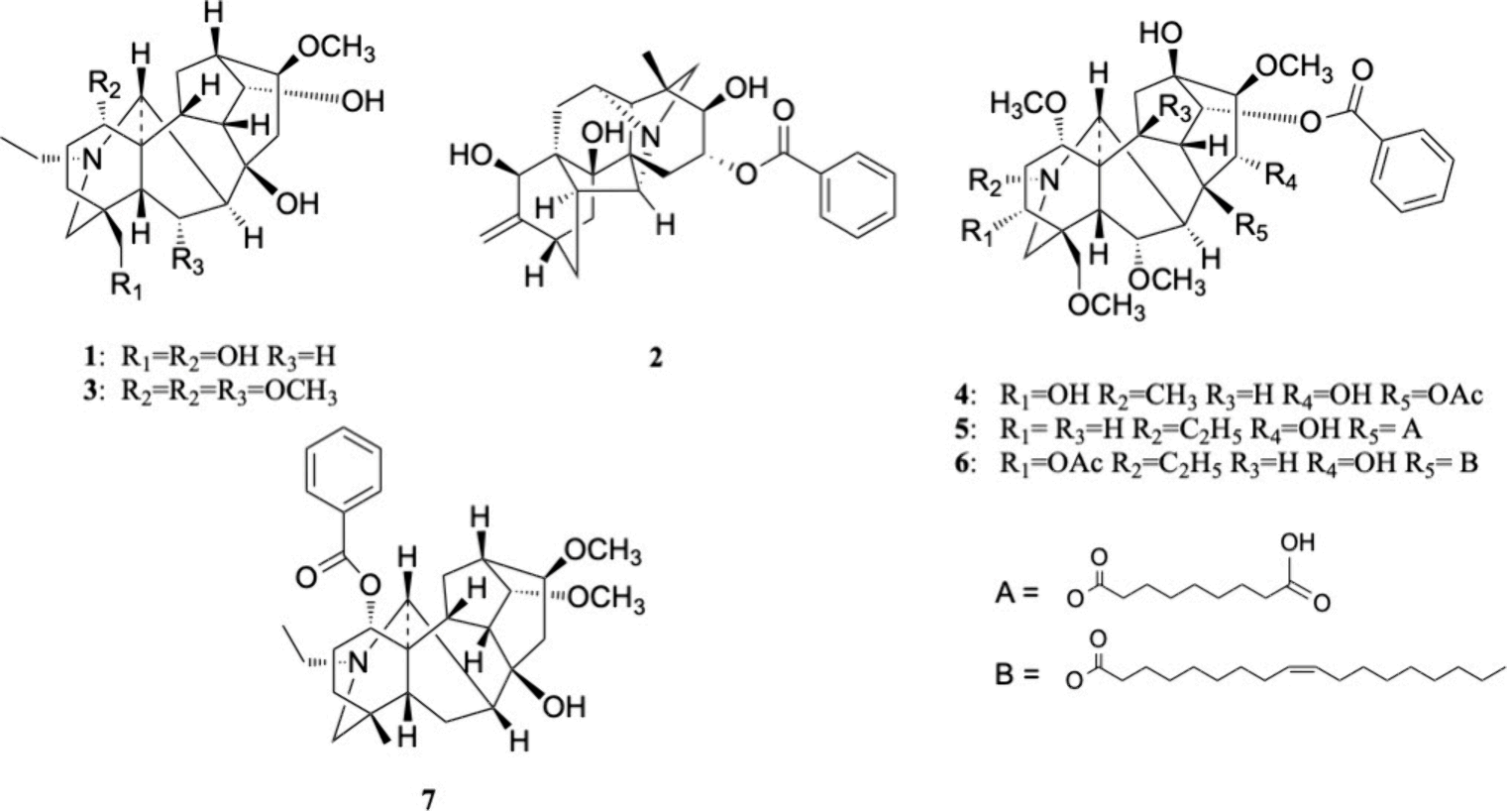
Tentative identification of bioactive features identified from the NP Analyst.

**Table 1:** Diterpenoid Alkaloids Tentatively Identified from Four *Aconitum* Species (ACo, AU, ACa, and AF) in This Study

						Detected in Species						
No.	R.T. (min)_Mass (*m*/*z*)	Identification	Formula	Adducts	PPM	ACo	AU	ACa	AF	AN	Activity Score	Citation
1	1.87_416.2431	Columbianine	C_22_H_35_NO_5_	[M + Na]^+^	4.3	×					0.04	^41^
2	3.68_450.2295	Ignavine	C_27_H_31_NO_5_	[M + H]^+^	3.3	×					0.07	^42^
3	4.75_474.2835	Chasmanine	C_25_H_41_NO_6_	[M + Na]^+^, [M + H – H_2_O – CH_2_O]^+^	0.6		×				0.07	^43^
4	4.88_632.3106	10-OH-hypaconitine	C_33_H_45_NO_11_	[M + H]^+^	5.5		×	×	×		0.06	^44^
5	5.75_774.4071	8-*O*-Azeloyl-14-benzoylaconine	C_41_H_59_NO_13_	[M + H]^+^	0.8		×		×		0.04	^45^
6	6.40_944.5516	3-Acetyl-8-ole-benzoylaconine	C_52_H_79_NO_12_	[M + Na]^+^	1.7		×				0.23	^46^
7	6.47_518.2877	1-*O*-Benzoylkarasamine	C_30_H_41_NO_5_	[M + Na]^+^	–1.0	×	×				0.04	^47^

Some toxic diester diterpenoid alkaloids do not appear in the network (Figure 6). For example, the well-known toxic compound aconitine was eliminated from the network because of its low CP score, and mesaconitine is not part of the network because of its low cluster score. The pharmacological activity of C-19 diterpenoid alkaloids from *Aconitum*, such as aconitine and its derivatives, is due to their affinity for voltage-dependent Na^+^ channels and the action on synaptosomal Na^+^ and Ca^+^ homeostasis.^32^ The effects of C-19 diterpenoid alkaloids on the CP osteosarcoma U2OS cell line has not been described previously.^33^ Natural C-19 norditerpenoid alkaloids have been reported to have significant inhibitory growth against the HePG2 cell line,^34^ but these alkaloids had no effect or only a slight effect against human tumor cell lines, such as A172, A549, HeLa, and Raji.^35^ Even though altering energy metabolism is an emerging cancer hallmark and processed *A. carmichaelii* roots (Fuzi) in some traditional Chinese medicine formulas are found to modulate energy metabolism, there is no direct evidence that *Aconitum* enhances mitochondrial oxidative phosphorylation in human cancer cells.^36^ Lappaconitine, approved for use as an analgesic in China,^37^ has also been shown to inhibit the growth of A549 cells by causing G0/G1 cell cycle arrest, apoptosis, and downregulation of cyclin E1 gene expression.^38^ Chodoeva et al. isolated 8-*O*-azeloyl-14-benzoylaconine, a new *Aconitum* alkaloid, from the roots of *A. karakolicum* Rapaics, and it showed antitumor properties against three human tumor cell lines, HCT-15, A549, and MCF-7.^39^ C-19 diester diterpenoid alkaloids, such as aconitine, mesaconitine, and hypaconitine, have not been detected in our LC-MS studies of American *Aconitum*, but some nonester diterpenoid alkaloids, with lower cardiotoxicity, have been identified as candidate active compounds using NP Analyst.

## Conclusions

In conclusion, we used a strategy to establish a network integrating both mass spectrometry data and Cell Painting activity as well as using an activity heuristic called a Cell Painting score to target the active compounds in American and non-American *Aconitum* species. Several active diterpenoid alkaloids have been tentatively identified. North American *Aconitum* species used in this study displayed notable activity in the Cell Painting assay. This network model does not include all of the known active diterpenoid alkaloids in *Aconitum* because of either low CP score or low cluster score. However, this network model is useful to help identify active compounds and can play a useful role in natural product high-throughput screening and drug discovery. Although *Aconitum* species have been explored for analgesic activity, diterpenoid alkaloids have been studied in a far greater range of bioactivities, from anti-inflammatory to anticancer.^40^ Using Cell Painting provides us with a different perspective to screen *Aconitum* for a wider scope of bioactive constituents that may be useful therapeutics.

## Experimental Section

### General Experimental Procedures

UPLC-qTOF-IMS was performed using an Acquity UPLC I-Class SYNAPT G2-Si hybrid quadrupole-traveling wave ion mobility (TWIM)-time-of-flight (TOF) mass spectrometer equipped with an electrospray ionization (ESI) source in positive mode (Waters Corporation, Milford, MA, USA). The Separation was performed on an ACQUITY UPLC HSS T3 C18 column (100 mm × 2.1 mm, 1.7 μm; Waters Corporation, Milford, MA, USA). The Cell Painting assay was performed according to the PerkinElmer PhenoVue Cell Painting kit (PING11) protocol. After the cells were cultured overnight in Corning 384-well microscopy assay plates (Corning, NY, USA), compounds were transferred to their corresponding assay using a Tecan Freedom EVO 100 high-throughput liquid handling robot (Männedorf, Switzerland) with attached V&P Scientific pin-tool (384 pins; V&P Scientific, Inc., San Diego, CA, USA). Then, the assay plants were stained with fluorescent probes (Shelton, CT, USA) after overnight incubation. Images were taken via a Molecular Devices ImageXpress micro XLS automated microscope (20× objective; channel: DAPI, FITC, TRITC, Cy5, and Texas Red).

### Plant Material

Two native American species, *A. uncinatum* and *A. columbianum*, along with three non-native species, *A. carmichaelii*, *A. fischeri*, and *A. napellus*, were selected for sampling. Eighteen *A. uncinatum* plants were collected off the Mountains to Sea Trail near Sylva, North Carolina, and 14 *A. columbianum* were harvested at San Juan National Forest, Colorado in August 2021. All these plants were collected in flowering season and harvested with different parts, including roots, stems, leaves, and flowers. Besides the wild collected samples, the roots of these plants were also harvested from cultivars. Roots of *A. columbianum* were collected from Sevenoaks Native Nursery (Albany, OR, USA) and roots of *A. uncinatum*, from Wood Thrush Native Nursery (Floyd, VA, USA). Four to ten replicates of three non-native species were ordered from Digging Dog Nursery (Albion, CA, USA) in August 2021. Parts of the roots were harvested immediately after receiving them. Flowers of *A. carmichaelii* were harvested two months later in a greenhouse. All harvested parts were dried under ambient temperature and then pulverized using a freezing mortar and pestle, which had been stored at −80 °C for at least one hour. All samples were stored at −80 °C until extraction.

### Chemicals

GR grade methanol was purchased from VWR Inc. (Bridgeport, PA, USA). Optima LC-MS grade methanol, acetonitrile, and formic acid were purchased from Thermo Fisher Scientific (Fair Lawn, NY, USA). Water was filtered with a Millipore Milli-Q system (EMD Millipore Corporation, Mahopac, NY, USA). Aconitine, mesaconitine, hypaconitine, talatisamine, benzyolaconine, benzoylmesaconine, and benzolhypaconine were purchased from Lemeitian Pharmaceutical Technology Co., Ltd. (Chengdu, Sichuan, China), and fuziline, neoline, and lappaconitine were obtained from Shanghai Yuanye Bio-Technology Co., Ltd. (Shanghai, China).

Dimethyl sulfoxide (DMSO) and Triton X-100 were ordered from Sigma-Aldrich (St. Louis, MO, USA). Electron microscopy grade paraformaldehyde aqueous (PFA) solution was purchased from Fisher Scientific (Fair Lawn, NY, USA). Brefeldin A, etoposide, and other library compounds were purchased from TargetMol (L4000, Boston, MA, USA). McCoy’s 5A medium, fetal bovine serum (FBS), trypsin-EDTA solution, and penicillin–streptomycin solution were purchased from ATCC (Manassas, VA, USA). PhenoVue Cell Painting kit (PING 11) comprising six validated, preoptimized fluorescent probes and associated PhenoVue Dye Diluent A were obtained from PerkinElmer (Shelton, CT, USA) for Cell Painting analysis. The fluorescent probes contain PhenoVue Hoechst 33342 nuclear stain, fluor 488 concanavalin A, 512 nucleic acid stain, fluor 555 – WGA, fluor 568 – phalloidin, and 641 mitochondrial stain. Hanks’ balanced salt solution (HBSS) was obtained from ATCC (Manassas, VA, USA).

### Extraction

Appropriately 0.25 g of 106 *Aconitum* samples (Table S1) was weighed and extracted with 10 mL of GR grade methanol. All samples were sonicated for 30 min (Misonix, Farmingdale, NY, USA) and then centrifuged at 2700 rpm for 5 min (IEC Centra GP8 benchtop centrifuge, IEC international equipment company, Needham, MA, USA). The supernatants were syringe filtered (0.45 μm) into 7 mL tared scintillation vials. Each filtered supernatant was dried under nitrogen gas, and the mass was calculated. All extracts were stored at −4 °C until further analysis.

### Compound Plate Preparation

*Aconitum* extracts were solubilized with DMSO at a stock concentration of 40 mg/mL. Then, 40 μL of the stock solutions were transferred to a Nunc 384-well polypropylene microplate (Thermo Fisher Scientific, Rochester, NY, USA) alongside control compounds. 2-fold serial dilution was performed four times for each sample. Rows A, B, O, and P and columns 1 and 24 were reserved for DMSO vehicle controls and buffering against edge effects. Columns 2 and 23 were reserved for positive controls with serial dilution of brefeldin A (C2: 25.6 mM to N2: ∼12.5 μM) and etoposide (C23: 25.6 mM to N23: ∼12.5 μM).

### U2OS Cell Culturing

Human bone osteosarcoma epithelial cells (U2OS) were obtained from ATCC (Manassas, VA, USA). The cells were cultured in McCoy’s 5A base medium supplemented with 10% FBS and 1% Pen/Strep at 37 °C, 5% CO_2_. Upon 80% confluence, cells were trypsinized following the standard procedure recommended by ATCC and cell concentration was determined using a BioRad TC20 automated cell counter (Hercules, CA, USA). Trypsinized cells were then seeded into Corning 384-well microscopy assay plates (Corning, NY, USA) at a volume of 40 μL, which is approximately 800 cells per well using a BioTek EL406 automated plate washer/dispenser (Agilent BioTek, Winooski, VT, USA). The cells were then incubated at 37 °C and 5% CO_2_ overnight before tests.

### Cell Painting High-Content Imaging

Test compounds were transferred from the compound plate to their corresponding overnight assay plates at a volume of 200 nL/well using a Tecan Freedom EVO 100 high-throughput liquid handling robot (Männedorf, Switzerland) with attached V&P Scientific pin-tool (384 pins; V&P Scientific, Inc., San Diego, CA, USA). Pinned assay plates were then placed back into the incubator at 37 °C and 5% CO_2_ overnight. After overnight incubation, all assay plates were treated, stained, and imaged according to the protocol outlined by the PerkinElmer PhenoVue Cell Painting kit (PING11). In brief, 30 μL of medium was aspirated from each well in the assay plate and replaced with a staining solution containing PhenoVue 641 mitochondrial Stain (500 nM final concentration) and PhenoVue dye diluent A (HBSS + 1% BSA). Assay plates were then incubated at 37 °C and 5% CO_2_ for 30 min in the dark before fixation using 16% PFA (3.2% final concentration). Fixed assay plates were incubated at room temperature for 20 min before washing with HBSS and permeabilization using a 0.1% Triton X-100 (250 mL) solution, followed by another 20 min incubation at RT. After a second HBSS wash, 30 μL of a second staining solution was added to each well containing the following dyes at their respective final concentrations: PhenoVue 512 Nucleic acid stain (3 μM), PhenoVue Fluor 488-Concanavalin A (960 nM), PhenoVue Fluor 555-WGA (43.7 nM), PhenoVue Hoechst 33342 Nuclear stain (8.12 μM), PhenoVue Fluor 568-Phalloidin (33 nM), and PhenoVue Dye Diluent A (HBSS + 1% BSA). After incubating for 30 min at room temperature, assay plates were lidded with adhesive aluminum foil (Fisher Scientific 12-56-398) prior to imaging via a Molecular Devices ImageXpress micro XLS automated microscope (20× objective; channel: DAPI, FITC, TRITC, Cy5, and Texas Red).

Batch-to-batch variation was controlled using the following approaches: first, we strictly adhered to version 3 of the Cell Painting protocol,^14,48^ including purchasing JUMP-CP^49^ approved dye kits from PerkinElmer; second, we included on every plate batch controls in serial dilution that align with historical data sets such as the TargetMol library (compounds include latrunculin B, brefeldin A, etoposide, and DMSO negative control); third, we have constructed a QC front-end that connects to a standardized CellProfiler segmentation and feature extraction pipeline, which performs illumination correction, outlier removal, and other normalization techniques while calculating batch reproducibility across DMSO control wells; last, we clustered our raw data systematically using a standardized method and gate cluster size using batch controls. In other Cell Painting deployments taking place months apart, we have shown that the eight positive controls recommended by the JUMP-CP Consortium consistently align and cluster together with historical replicates.

### Data Normalization, Analysis, and Feature Selection

Cell Painting images were processed using a modified protocol adapted from Bray et al. and proceeded as follows.^55^ Cell segmentation and feature extraction via CellProfiler^50^ produced 2,090 raw features on the basis of cell count and fluorescence intensity, granularity, texture, area, shape, and overlap for each of the five channels. Raw feature values for each sample were normalized to the DMSO vehicle control using the robust histogram-difference method (HistDiff).^51^ In order to identify potential modes of action from sample compounds, a reference library containing 4,400 compounds with known biological function (TargetMol, L4000) was screened in Cell Painting at 50 μM with the corresponding image data processed and normalized as described above; then, it was combined with the sample compound data. The CP score^52^ was calculated as the square root of the sum of the squares of all 2,090 feature values for each sample and reference library compound. All sample compounds and reference library compounds with CP scores at or below the DMSO vehicle controls were removed, resulting in 2,240 samples with nonzero feature profiles. Feature selection was performed via the Fast Correlation-Based Filtering (FCBF) algorithm on Orange^53^ to identify features with maximal correlation distribution while removing redundant features, resulting in a reduced subset of 435 features. The trimmed data set, containing a combined 2,240 sample and reference library compounds multiplied by 429 unique features (CP fingerprints), was then hierarchically clustered using Spearman correlations and Ward linkage parameters to group like fingerprints together. Hierarchical clustering trees and heatmaps were visualized using Orange.^53^

### UPLC-qTOF-IMS

All *Aconitum* extracts were dissolved with LC-MS grade methanol to a 2 mg/mL concentration and filtered in an LC-MS vial. All measurements were conducted on an Acquity UPLC I-Class SYNAPT G2-Si hybrid quadrupole-traveling wave ion mobility (TWIM)-time-of-flight (TOF) mass spectrometer equipped with an electrospray ionization (ESI) source in positive mode (Waters Corporation, Milford, MA, USA). The separation used an ACQUITY UPLC HSS T3 C18 column (100 mm × 2.1 mm, 1.7 μm; Waters Corporation, Milford, MA, USA) with the following gradients of (A) water with 0.01% formic acid and (B) acetonitrile with 0.01% formic acid: 0–0.3 min, 95% A; 0.3–9.1 min, 95–10% A; 9.1–10.7 min, 10–2% A; 10.7–14.0 min, 2% A. The flow rate is 500 μL/min, and the column temperature is 40 °C. HDMS^E^ mode was set to alternate between collision energies of 0 and 30 eV per 0.25 s. Every 10 s, 200 pg/μL leucine enkephalin lockspray infusion was enabled within electrospray mode. Mass spectra ranges were set up as 50–1500 *m*/*z* at a 4 Hz scan rate in continuum mode without applying lockmass correction.

### IMS Data Analysis

The mass spectrometry data was analyzed using Progenesis QI software (Waters, Milford, MA, USA). After all mass raw data was imported to Progenesis QI, the retention times of all injections were aligned automatically, and peaks were picked from 0 to 11 min with default sensitivity and filtered with 1% of base peak intensity. These peaks were deconvoluted based on the retention time and adduct parameters, which was edited based on previous *Aconitum* studies. An in-house *Aconitum* database containing 1,166 compounds was created from SciFinder to help with compound identification. Each compound was reviewed by using compound statistics to determine their distribution in each injection.

### NP Analyst Network

All mass spectrometry data were exported from Progenesis QI software (Waters, Milford, MA, USA). The strength of the phenotype activity for each mass spectrometric feature was calculated by taking the sum of the mean of the squares of the bioactivity values for each extract containing a given mass spectrometric feature, which is called CP score in this study.^54^ The consistency of each mass spectrometric feature is determined by taking the average Pearson similarity scores between the biological fingerprints of all extracts containing a given mass spectrometric feature, which is referred to as cluster score.^54^ The CP score file containing plant sample feature profiles (ranging from 0 to 1) and the mass spectrometry csv file of all plant samples were uploaded to the NP Analyst Web site (https://www.npanalyst.org/). The network was created after selecting the activity score of 0.03 and a cluster score of 0.5 (ranging from −1 to 1) as minimum thresholds to be included in the network following NP analyst protocols.^16^

## Supplementary Material



## Notes

*Aconitum* sample list; target enzymes, pathways, and cell painting phenotypes of select controls; full hierarchical clusters of *Aconitum* samples, control compounds, and members of the TargetMol library; cell painting heatmap of select *Aconitum*, controls, and TargetMol library clusters; chromatograms of different parts from American *Aconitum*; principal component analysis of *Aconitum uncinatum* leaf samples; S-plot of *Aconitum uncinatum* leaf samples (PDF)
