# Supplementary material for: Prediction of Bioactive Metabolites from American Aconitum Using Network Integrating Cellular Morphological Profiling and Mass Spectrometry Data
Source: J Nat Prod. 2025 Jul 11;88(7):1557–67. doi: 10.1021/acs.jnatprod.4c01458 (PMC12305659; doi:10.1021/acs.jnatprod.4c01458)
Supplement: Supplementary file 1 [file np4c01458_si_001.pdf]

Prediction of Bioactive Metabolites from American *Aconitum* Using Network Integrating Cellular Morphological Profiling and Mass Spectrometry Data

Yi Zhao,[†‡] Dennis Y. Liu,[§] Trevor N. Clark,[§] Roger G. Linington,[§] Edward J. Kennelly[†‡*]

[†] Department of Biological Sciences, Lehman College, The City University of New York, 250 Bedford Park Boulevard West, Bronx, New York 10468, USA

[‡] Biology Ph.D. Program, The Graduate Center, The City University of New York, 365 Fifth Avenue, New York, NY 10016, USA.

[§] Department of Chemistry, Simon Fraser University, 8888 University Drive, Burnaby, British Columbia, Canada V5A 1S6.

* Corresponding author. E-mail: Edward.kennelly@lehman.cuny.edu (E.J. Kennelly). Phone: +1 718 960 1105. Fax: +1 718 960 8236.

Contents

Table S1. *Aconitum* sample list ---3

Table S2. Target enzymes, pathways, and cell painting phenotypes for select controls ----------8

Figure S1. Full hierarchical clusters of *Aconitum* samples, control compounds, and members of the TargetMol library---9

Figure S2. Cell Painting heatmap of select *Aconitum*, control, and the TargetMol librsary clusters--11

Figure S3. Chromatograms of different parts from American *Aconitum*--------------------------13

Figure S4. Principal component analysis of *Aconitum uncinatum* leaf samples------------------14

Figure S5. S-plot of *Aconitum uncinatum* leaf samples--15

Table S1. ***Aconitum*** **sample list**

Sample ID	Sample Code	Genus	Species	Native to	Location	Part	Collecting time
AU-005	08172021_01S	*Aconitum*	*uncinatum*	North America	Mountains-to-Sea Trail, Sylva, NC	Stems	8/17/21
AU-007	08172021_02R	*Aconitum*	*uncinatum*	North America	Mountains-to-Sea Trail, Sylva, NC	Roots	8/17/21
AU-009	08172021_02S	*Aconitum*	*uncinatum*	North America	Mountains-to-Sea Trail, Sylva, NC	Stems	8/17/21
AU-010	08172021_02L	*Aconitum*	*uncinatum*	North America	Mountains-to-Sea Trail, Sylva, NC	Leaves	8/17/21
AU-011	08172021_03S	*Aconitum*	*uncinatum*	North America	Mountains-to-Sea Trail, Sylva, NC	Stems	8/17/21
AU-014	08172021_03L	*Aconitum*	*uncinatum*	North America	Mountains-to-Sea Trail, Sylva, NC	Leaves	8/17/21
AU-015	08172021_04R	*Aconitum*	*uncinatum*	North America	Mountains-to-Sea Trail, Sylva, NC	Roots	8/17/21
AU-019	08172021_05R	*Aconitum*	*uncinatum*	North America	Mountains-to-Sea Trail, Sylva, NC	Roots	8/17/21
AU-020	08172021_05S	*Aconitum*	*uncinatum*	North America	Mountains-to-Sea Trail, Sylva, NC	Stems	8/17/21
AU-022	08172021_05L	*Aconitum*	*uncinatum*	North America	Mountains-to-Sea Trail, Sylva, NC	Leaves	8/17/21
AU-023	08172021_06L	*Aconitum*	*uncinatum*	North America	Mountains-to-Sea Trail, Sylva, NC	Leaves	8/17/21
AU-024	08172021_06S	*Aconitum*	*uncinatum*	North America	Mountains-to-Sea Trail, Sylva, NC	Stems	8/17/21
AU-025	08172021_06R	*Aconitum*	*uncinatum*	North America	Mountains-to-Sea Trail, Sylva, NC	Roots	8/17/21
AU-027	08172021_07R	*Aconitum*	*uncinatum*	North America	Mountains-to-Sea Trail, Sylva, NC	Roots	8/17/21
AU-028	08172021_07S	*Aconitum*	*uncinatum*	North America	Mountains-to-Sea Trail, Sylva, NC	Stems	8/17/21
AU-030	08172021_07L	*Aconitum*	*uncinatum*	North America	Mountains-to-Sea Trail, Sylva, NC	Leaves	8/17/21
AU-031	08172021_08R	*Aconitum*	*uncinatum*	North America	Mountains-to-Sea Trail, Sylva, NC	Roots	8/17/21
AU-032	08172021_08S	*Aconitum*	*uncinatum*	North America	Mountains-to-Sea Trail, Sylva, NC	Stems	8/17/21
AU-034	08172021_08L	*Aconitum*	*uncinatum*	North America	Mountains-to-Sea Trail, Sylva, NC	Leaves	8/17/21
AU-035	08172021_09S	*Aconitum*	*uncinatum*	North America	Mountains-to-Sea Trail, Sylva, NC	Stems	8/17/21
AU-036	08172021_09L	*Aconitum*	*uncinatum*	North America	Mountains-to-Sea Trail, Sylva, NC	Leaves	8/17/21
AU-037	08172021_10R	*Aconitum*	*uncinatum*	North America	Mountains-to-Sea Trail, Sylva, NC	Roots	8/17/21
AU-038	08172021_10S	*Aconitum*	*uncinatum*	North America	Mountains-to-Sea Trail, Sylva, NC	Stems	8/17/21
AU-040	08172021_10L	*Aconitum*	*uncinatum*	North America	Mountains-to-Sea Trail, Sylva, NC	Leaves	8/17/21
AU-041	08172021_11S	*Aconitum*	*uncinatum*	North America	Mountains-to-Sea Trail, Sylva, NC	Stems	8/17/21

AU-042	08172021_11R	*Aconitum*	*uncinatum*	North America	Mountains-to-Sea Trail, Sylva, NC	Roots	8/17/21
AU-044	08172021_11L	*Aconitum*	*uncinatum*	North America	Mountains-to-Sea Trail, Sylva, NC	Leaves	8/17/21
AU-045	08172021_12S	*Aconitum*	*uncinatum*	North America	Mountains-to-Sea Trail, Sylva, NC	Stems	8/17/21
AU-046	08172021_12R	*Aconitum*	*uncinatum*	North America	Mountains-to-Sea Trail, Sylva, NC	Roots	8/17/21
AU-049	08172021_13S	*Aconitum*	*uncinatum*	North America	Mountains-to-Sea Trail, Sylva, NC	Stems	8/17/21
AU-050	08172021_13L	*Aconitum*	*uncinatum*	North America	Mountains-to-Sea Trail, Sylva, NC	Leaves	8/17/21
AU-051	08172021_14S	*Aconitum*	*uncinatum*	North America	Mountains-to-Sea Trail, Sylva, NC	Stems	8/17/21
AU-052	08172021_15S	*Aconitum*	*uncinatum*	North America	Mountains-to-Sea Trail, Sylva, NC	Stems	8/17/21
AU-054	08172021_15R	*Aconitum*	*uncinatum*	North America	Mountains-to-Sea Trail, Sylva, NC	Roots	8/17/21
AU-055	08172021_15L	*Aconitum*	*uncinatum*	North America	Mountains-to-Sea Trail, Sylva, NC	Leaves	8/17/21
AU-056	08172021_16S	*Aconitum*	*uncinatum*	North America	Mountains-to-Sea Trail, Sylva, NC	Stems	8/17/21
AU-058	08172021_17S	*Aconitum*	*uncinatum*	North America	Mountains-to-Sea Trail, Sylva, NC	Stems	8/17/21
AU-060	08172021_17L	*Aconitum*	*uncinatum*	North America	Mountains-to-Sea Trail, Sylva, NC	Leaves	8/17/21
AU-062	08172021_18L	*Aconitum*	*uncinatum*	North America	Mountains-to-Sea Trail, Sylva, NC	Leaves	8/17/21
AU-068	09212021_14R	*Aconitum*	*uncinatum*	North America	Wood Thrush Native Nursery	Roots	11/12/21
AU-069	09212021_16R	*Aconitum*	*uncinatum*	North America	Wood Thrush Native Nursery	Roots	11/12/21
AU-071	09212021_17R	*Aconitum*	*uncinatum*	North America	Wood Thrush Native Nursery	Roots	11/12/21
ACo-006	08032021_02S	*Aconitum*	*columbianum*	North America	a trail near Taylor Creek, San Juan National Forest, CO	Stems	8/3/21
ACo-007	08032021_02R	*Aconitum*	*columbianum*	North America	a trail near Taylor Creek, San Juan National Forest, CO	Roots	8/3/21
ACo-008	08032021_02L	*Aconitum*	*columbianum*	North America	a trail near Taylor Creek, San Juan National Forest, CO	Leaves	8/3/21
ACo-009	08032021_03S	*Aconitum*	*columbianum*	North America	a trail near Taylor Creek, San Juan National Forest, CO	Stems	8/3/21
ACo-011	08032021_03L	*Aconitum*	*columbianum*	North America	a trail near Taylor Creek, San Juan National Forest, CO	Leaves	8/3/21
ACo-012	08032021_03R	*Aconitum*	*columbianum*	North America	a trail near Taylor Creek, San Juan National Forest, CO	Roots	8/3/21

ACo-013	08032021_04L	*Aconitum*	*columbianum*	North America	a trail near Taylor Creek, San Juan National Forest, CO	Leaves	8/3/21
ACo-014	08032021_04S	*Aconitum*	*columbianum*	North America	a trail near Taylor Creek, San Juan National Forest, CO	Stems	8/3/21
ACo-017	08032021_05L	*Aconitum*	*columbianum*	North America	a trail near Taylor Creek, San Juan National Forest, CO	Leaves	8/3/21
ACo-023	08032021_06L	*Aconitum*	*columbianum*	North America	a trail near Taylor Creek, San Juan National Forest, CO	Leaves	8/3/21
ACo-024	08032021_06S	*Aconitum*	*columbianum*	North America	a trail near Taylor Creek, San Juan National Forest, CO	Stems	8/3/21
ACo-025	08032021_07R	*Aconitum*	*columbianum*	North America	a trail near Taylor Creek, San Juan National Forest, CO	Roots	8/3/21
ACo-031	08032021_08S	*Aconitum*	*columbianum*	North America	a trail near Taylor Creek, San Juan National Forest, CO	Stems	8/3/21
ACo-032	08032021_08L	*Aconitum*	*columbianum*	North America	a trail near Taylor Creek, San Juan National Forest, CO	Leaves	8/3/21
ACo-033	08032021_09R	*Aconitum*	*columbianum*	North America	a trail near Taylor Creek, San Juan National Forest, CO	Roots	8/3/21
ACo-034	08032021_09L	*Aconitum*	*columbianum*	North America	a trail near Taylor Creek, San Juan National Forest, CO	Leaves	8/3/21
ACo-035	08032021_09S	*Aconitum*	*columbianum*	North America	a trail near Taylor Creek, San Juan National Forest, CO	Stems	8/3/21
ACo-038	08032021_10L	*Aconitum*	*columbianum*	North America	a trail near Taylor Creek, San Juan National Forest, CO	Leaves	8/3/21
ACo-039	08032021_10R	*Aconitum*	*columbianum*	North America	a trail near Taylor Creek, San Juan National Forest, CO	Roots	8/3/21
ACo-040	08032021_10S	*Aconitum*	*columbianum*	North America	a trail near Taylor Creek, San Juan National Forest, CO	Stems	8/3/21
ACo-041	08032021_11R	*Aconitum*	*columbianum*	North America	a trail near Taylor Creek, San Juan National Forest, CO	Roots	8/3/21

ACo-042	08032021_11S	*Aconitum*	*columbianum*	North America	a trail near Taylor Creek, San Juan National Forest, CO	Stems	8/3/21
ACo-044	08032021_11L	*Aconitum*	*columbianum*	North America	a trail near Taylor Creek, San Juan National Forest, CO	Leaves	8/3/21
ACo-045	08032021_12R	*Aconitum*	*columbianum*	North America	a trail near Taylor Creek, San Juan National Forest, CO	Roots	8/3/21
ACo-046	08032021_12S	*Aconitum*	*columbianum*	North America	a trail near Taylor Creek, San Juan National Forest, CO	Stems	8/3/21
ACo-048	08032021_12L	*Aconitum*	*columbianum*	North America	a trail near Taylor Creek, San Juan National Forest, CO	Leaves	8/3/21
ACo-052	07262021_14L	*Aconitum*	*columbianum*	North America	a trail near Taylor Creek, San Juan National Forest, CO	Leaves	7/26/21
ACo-053	07262021_14S	*Aconitum*	*columbianum*	North America	a trail near Taylor Creek, San Juan National Forest, CO	Stems	7/26/21
ACo-054	07262021_14R	*Aconitum*	*columbianum*	North America	a trail near Taylor Creek, San Juan National Forest, CO	Roots	7/26/21
ACo-056	10292021_1R	*Aconitum*	*columbianum*	North America	Sevenoaks Native Nursery	Roots	10/29/21
ACo-057	10292021_2R	*Aconitum*	*columbianum*	North America	Sevenoaks Native Nursery	Roots	11/2/21
ACo-058	10292021_3R	*Aconitum*	*columbianum*	North America	Sevenoaks Native Nursery	Roots	11/2/21
ACo-059	10292021_4R	*Aconitum*	*columbianum*	North America	Sevenoaks Native Nursery	Roots	11/2/21
ACo-060	10292021_5R	*Aconitum*	*columbianum*	North America	Sevenoaks Native Nursery	Roots	11/2/21
AF-002	08232021_02R	*Aconitum*	*fischeri*	East Asia and Siberia	Digging Dog Nursery	Roots	8/23/21
AF-003	08232021_02F	*Aconitum*	*fischeri*	East Asia and Siberia	Digging Dog Nursery	Flowers	8/23/21
AF-005	08232021_04R	*Aconitum*	*fischeri*	East Asia and Siberia	Digging Dog Nursery	Roots	8/23/21
AF-006	08232021_04F	*Aconitum*	*fischeri*	East Asia and Siberia	Digging Dog Nursery	Flowers	8/23/21
AF-007	08232021_05R	*Aconitum*	*fischeri*	East Asia and Siberia	Digging Dog Nursery	Roots	8/23/21

AF-008	08232021_06R	*Aconitum*	*fischeri*	East Asia and Siberia	Digging Dog Nursery	Roots	8/23/21
AF-009	08232021_06F	*Aconitum*	*fischeri*	East Asia and Siberia	Digging Dog Nursery	Flowers	12/5/21
AF-010	08232021_07R	*Aconitum*	*fischeri*	East Asia and Siberia	Digging Dog Nursery	Roots	8/23/21
AF-012	08232021_09R	*Aconitum*	*fischeri*	East Asia and Siberia	Digging Dog Nursery	Roots	8/23/21
ACa-001	08232021_01R	*Aconitum*	*carmichaelii*	China	Digging Dog Nursery	Roots	8/23/21
ACa-002	08232021_01F	*Aconitum*	*carmichaelii*	China	Digging Dog Nursery	Flowers	10/31/21
ACa-003	08232021_02R	*Aconitum*	*carmichaelii*	China	Digging Dog Nursery	Roots	8/23/21
ACa-004	08232021_02F	*Aconitum*	*carmichaelii*	China	Digging Dog Nursery	Flowers	11/22/21
ACa-005	08232021_03R	*Aconitum*	*carmichaelii*	China	Digging Dog Nursery	Roots	8/23/21
ACa-006	08232021_03F	*Aconitum*	*carmichaelii*	China	Digging Dog Nursery	Flowers	10/15/21
ACa-008	08232021_04F	*Aconitum*	*carmichaelii*	China	Digging Dog Nursery	Roots	11/8/21
ACa-009	08232021_05R	*Aconitum*	*carmichaelii*	China	Digging Dog Nursery	Roots	8/23/21
ACa-010	08232021_05F	*Aconitum*	*carmichaelii*	China	Digging Dog Nursery	Flowers	10/28/21
ACa-012	08232021_06F	*Aconitum*	*carmichaelii*	China	Digging Dog Nursery	Flowers	11/3/21
ACa-013	08232021_07R	*Aconitum*	*carmichaelii*	China	Digging Dog Nursery	Roots	8/23/21
ACa-014	08232021_07F	*Aconitum*	*carmichaelii*	China	Digging Dog Nursery	Flowers	10/29/21
ACa-015	08232021_08R	*Aconitum*	*carmichaelii*	China	Digging Dog Nursery	Roots	8/23/21
ACa-016	08232021_08F	*Aconitum*	*carmichaelii*	China	Digging Dog Nursery	Flowers	10/17/21
ACa-017	08232021_09R	*Aconitum*	*carmichaelii*	China	Digging Dog Nursery	Roots	8/23/21
ACa-019	08232021_10R	*Aconitum*	*carmichaelii*	China	Digging Dog Nursery	Roots	8/23/21
ACa-020	08232021_10F	*Aconitum*	*carmichaelii*	China	Digging Dog Nursery	Flowers	8/23/21
AN-002	08232021_02R	*Aconitum*	*napellus*	Europe	Digging Dog Nursery	Roots	8/23/21
AN-003	08232021_03R	*Aconitum*	*napellus*	Europe	Digging Dog Nursery	Roots	8/23/21
AN-006	08232021_06R	*Aconitum*	*napellus*	Europe	Digging Dog Nursery	Roots	8/23/21
AN-009	08232021_09R	*Aconitum*	*napellus*	Europe	Digging Dog Nursery	Roots	8/23/21

Table S2 Target enzymes, pathways, and cell painting phenotypes for select controls

Cluster	Compound	Target/Pathway	Class
40	Asenapine Maleate	Serotonin/Norepinephrine/Dopamine/Histamine Receptors Antagonist	Antipsychotic
40	Naftifine HCl	Sterol Biosynthesis	Antifungal
40	Jujuboside B	Collagen-, Thrombin-, AA-, and ADP-induced Aggregation Inhibitor	Traditional Chinese medicine
40	BAY 87-2243	HIF-1/Mitochondrial Complex I Inhibitor	
40	Darifenacin HBr	M3 Muscarinic Receptor Antagonist	Anticholinergic
40	Epacadostat	IDO1 Inhibitor	
40	Malitracen HCl	Dopamine D1/2 Receptor Antagonist	Antidepressant
40	Bedaquiline	ATP Synthesis	Antimycobacterial
40	Acitretin	ADAM10/Mitochondrial Phosphorylation	Retinoid
40	EPZ020411 2HCl	PRMT6 Inhibitor	Antiproliferative
40	Lurasidone HCl	D2/5-HT2/5-HT7/alpha2A/alpha2C Receptor Antagonist	Antipsychotic
40	FTI 277 HCl	FTase Inhibitor	Antiproliferative
41	Megestrol Acetate	Progesterone Receptor Agonist	Progestogen
41	Nepicastat HCl	Dopamine-beta-hydroxylase Inhibitor	Antipsychotic
41	L755507	Beta3-AR Agonist	Neuromodulator
42	NSC319726	P53R175 Inhibitor	Antiproliferative
93	Latrunculin B	G/F-actin Inhibitor	Natural Product
119	Balicatib	Cathepsin K	Antiosteoporotic
119	J147	N/A	Neuromodulator

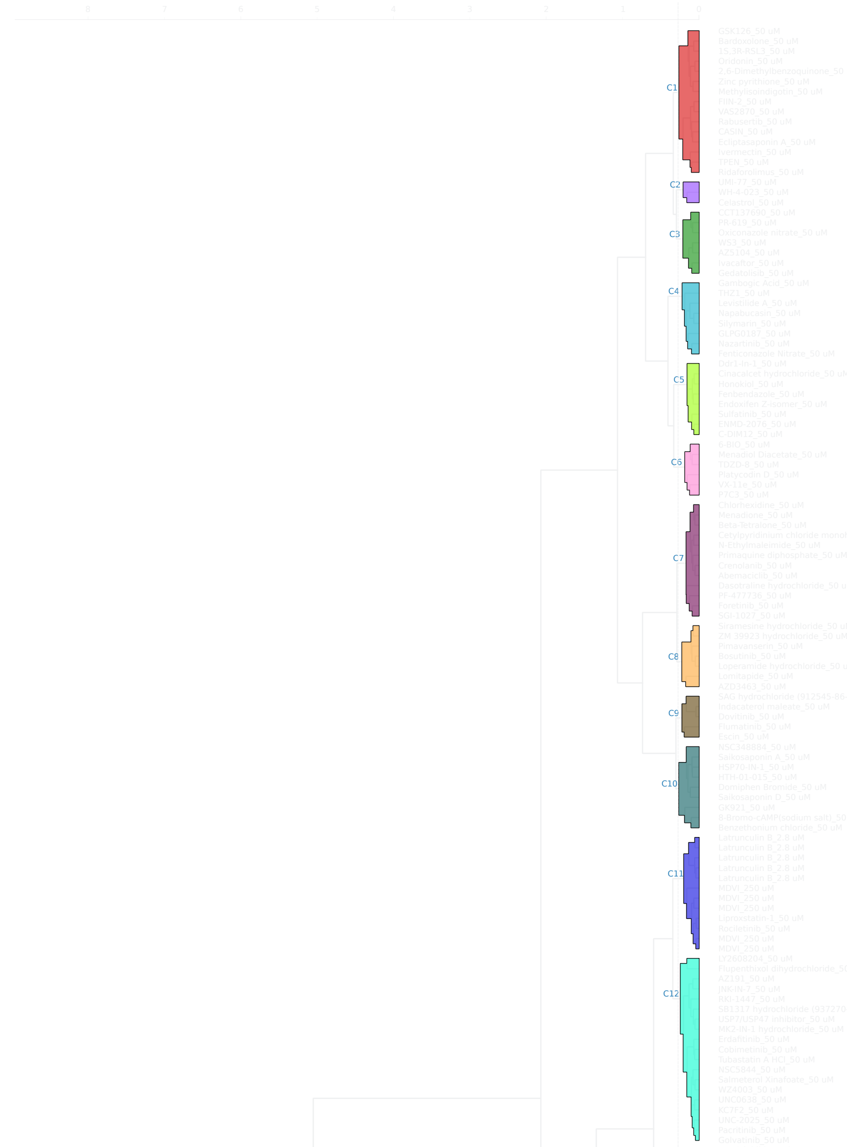
8
7
6
5
4
3
2
1
0
C1
C2
C3
C4
C5
C6
C7
C8
C9
C10
C11
C12
GSK126_50 uM
Bardoxolone_50 uM
1S,3R-RSL3_50 uM
Oridonin_50 uM
2,6-Dimethylbenzoquinone_50
Zinc pyrithione_50 uM
Methylisoindigotin_50 uM
FIIN-2_50 uM
VAS2870_50 uM
Rabusertib_50 uM
CASIN_50 uM
Ecliptasaponin A_50 uM
Ivermectin_50 uM
TPEN_50 uM
Ridaforolimus_50 uM
UMI-77_50 uM
WH-4-023_50 uM
Celastrol_50 uM
CCT137690_50 uM
PR-619_50 uM
Oxiconazole nitrate_50 uM
WS3_50 uM
AZ5104_50 uM
Ivacaftor_50 uM
Gedatolisib_50 uM
Gambogic Acid_50 uM
THZ1_50 uM
Levistilide A_50 uM
Napabucasin_50 uM
Silymarin_50 uM
GLPG0187_50 uM
Nazartinib_50 uM
Fenticonazole Nitrate_50 uM
Ddr1-In-1_50 uM
Cinacalcet hydrochloride_50 uM
Honokiol_50 uM
Fenbendazole_50 uM
Endoxifen Z-isomer_50 uM
Sulfatinib_50 uM
ENMD-2076_50 uM
C-DIM12_50 uM
6-BIO_50 uM
Menadiol Diacetate_50 uM
TDZD-8_50 uM
Platycodin D_50 uM
VX-11e_50 uM
P7C3_50 uM
Chlorhexidine_50 uM
Menadione_50 uM
Beta-Tetralone_50 uM
Cetylpyridinium chloride monoh
N-Ethylmaleimide_50 uM
Primaquine diphosphate_50 uM
Crenolanib_50 uM
Abemaciclib_50 uM
Dasotraline hydrochloride_50 u
PF-477736_50 uM
Foretinib_50 uM
SGI-1027_50 uM
Siramesine hydrochloride_50 u
ZM 39923 hydrochloride_50 uM
Pimavanserin_50 uM
Bosutinib_50 uM
Loperamide hydrochloride_50 u
Lomitapide_50 uM
AZD3463_50 uM
SAG hydrochloride (912545-86-
Indacaterol maleate_50 uM
Dovitinib_50 uM
Flumatinib_50 uM
Escin_50 uM
NSC348884_50 uM
Saikosaponin A_50 uM
HSP70-IN-1_50 uM
HTH-01-015_50 uM
Domiphen Bromide_50 uM
Saikosaponin D_50 uM
GK921_50 uM
8-Bromo-cAMP(sodium salt)_50
Benzethonium chloride_50 uM
Latrunculin B_2.8 uM
Latrunculin B_2.8 uM
Latrunculin B_2.8 uM
Latrunculin B_2.8 uM
Latrunculin B_2.8 uM
MDVI_250 uM
MDVI_250 uM
MDVI_250 uM
Liproxstatin-1_50 uM
Rociletinib_50 uM
MDVI_250 uM
MDVI_250 uM
LY2608204_50 uM
Flupenthixol dihydrochloride_50
AZ191_50 uM
JNK-IN-7_50 uM
RKI-1447_50 uM
SB1317 hydrochloride (937270-
USP7/USP47 inhibitor_50 uM
MK2-IN-1 hydrochloride_50 uM
Erdafitinib_50 uM
Cobimetinib_50 uM
Tubastatin A HCl_50 uM
NSC5844_50 uM
Salmeterol Xinafoate_50 uM
WZ4003_50 uM
UNC0638_50 uM
KC7F2_50 uM
UNC-2025_50 uM
Pacritinib_50 uM
Golvatinib_50 uM

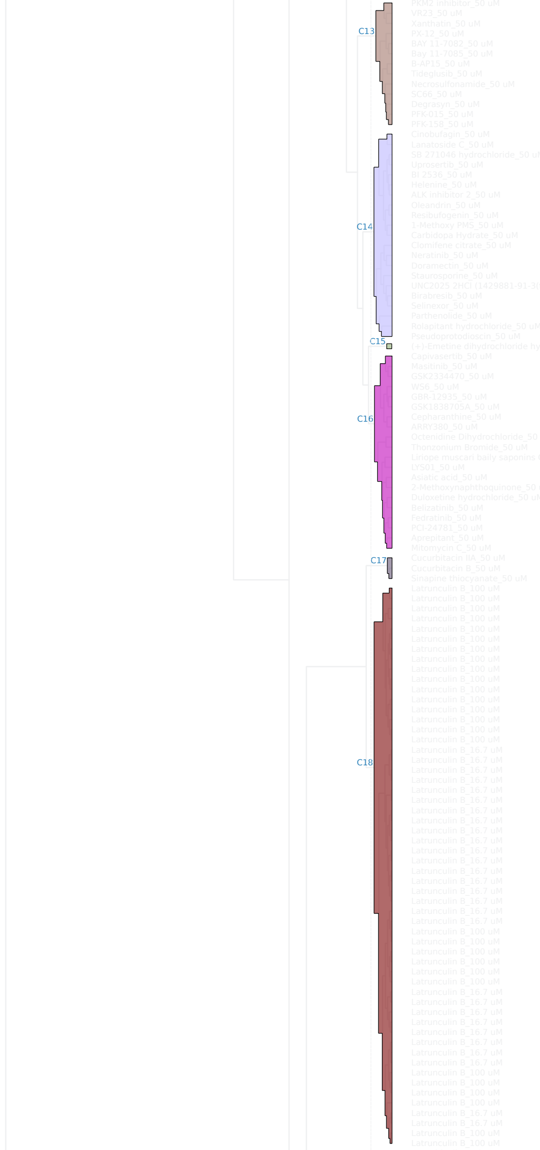
C13
C14
C15
C16
C17
C18
PKM2 inhibitor_50 uM
VR23_50 uM
Xanthatin_50 uM
PX-12_50 uM
BAY 11-7082_50 uM
Bay 11-7085_50 uM
B-AP15_50 uM
Tideglusib_50 uM
Necrosulfonamide_50 uM
SC66_50 uM
Degrasyn_50 uM
PFK-015_50 uM
PFK-158_50 uM
Cinobufagin_50 uM
Lanatoside C_50 uM
SB 271046 hydrochloride_50 u
Uprosertib_50 uM
BI 2536_50 uM
Helenine_50 uM
ALK inhibitor 2_50 uM
Oleandrin_50 uM
Resibufogenin_50 uM
1-Methoxy PMS_50 uM
Carbidopa Hydrate_50 uM
Clomifene citrate_50 uM
Neratinib_50 uM
Doramectin_50 uM
Staurosporine_50 uM
UNC2025 2HCl (1429881-91-3(
Birabresib_50 uM
Selinexor_50 uM
Parthenolide_50 uM
Rolapitant hydrochloride_50 uM
Pseudoprotodioscin_50 uM
(+)-Emetine dihydrochloride hy
Capivasertib_50 uM
Masitinib_50 uM
GSK2334470_50 uM
WS6_50 uM
GBR-12935_50 uM
GSK1838705A_50 uM
Cepharanthine_50 uM
ARRY380_50 uM
Octenidine Dihydrochloride_50
Thonzonium Bromide_50 uM
Liriope muscari baily saponins (
LYS01_50 uM
Asiatic acid_50 uM
2-Methoxynaphthoquinone_50
Duloxetine hydrochloride_50 uM
Belizatinib_50 uM
Fedratinib_50 uM
PCI-24781_50 uM
Aprepitant_50 uM
Mitomycin C_50 uM
Cucurbitacin IIA_50 uM
Cucurbitacin B_50 uM
Sinapine thiocyanate_50 uM
Latrunculin B_100 uM
Latrunculin B_100 uM
Latrunculin B_100 uM
Latrunculin B_100 uM
Latrunculin B_100 uM
Latrunculin B_100 uM
Latrunculin B_100 uM
Latrunculin B_100 uM
Latrunculin B_100 uM
Latrunculin B_100 uM
Latrunculin B_100 uM
Latrunculin B_100 uM
Latrunculin B_100 uM
Latrunculin B_100 uM
Latrunculin B_100 uM
Latrunculin B_100 uM
Latrunculin B_16.7 uM
Latrunculin B_16.7 uM
Latrunculin B_16.7 uM
Latrunculin B_16.7 uM
Latrunculin B_16.7 uM
Latrunculin B_16.7 uM
Latrunculin B_16.7 uM
Latrunculin B_16.7 uM
Latrunculin B_16.7 uM
Latrunculin B_16.7 uM
Latrunculin B_16.7 uM
Latrunculin B_16.7 uM
Latrunculin B_16.7 uM
Latrunculin B_16.7 uM
Latrunculin B_16.7 uM
Latrunculin B_16.7 uM
Latrunculin B_16.7 uM
Latrunculin B_16.7 uM
Latrunculin B_100 uM
Latrunculin B_100 uM
Latrunculin B_100 uM
Latrunculin B_100 uM
Latrunculin B_100 uM
Latrunculin B_100 uM
Latrunculin B_16.7 uM
Latrunculin B_16.7 uM
Latrunculin B_16.7 uM
Latrunculin B_16.7 uM
Latrunculin B_16.7 uM
Latrunculin B_16.7 uM
Latrunculin B_16.7 uM
Latrunculin B_16.7 uM
Latrunculin B_100 uM
Latrunculin B_100 uM
Latrunculin B_100 uM
Latrunculin B_100 uM
Latrunculin B_16.7 uM
Latrunculin B_16.7 uM
Latrunculin B_100 uM
Latrunculin B_100 uM

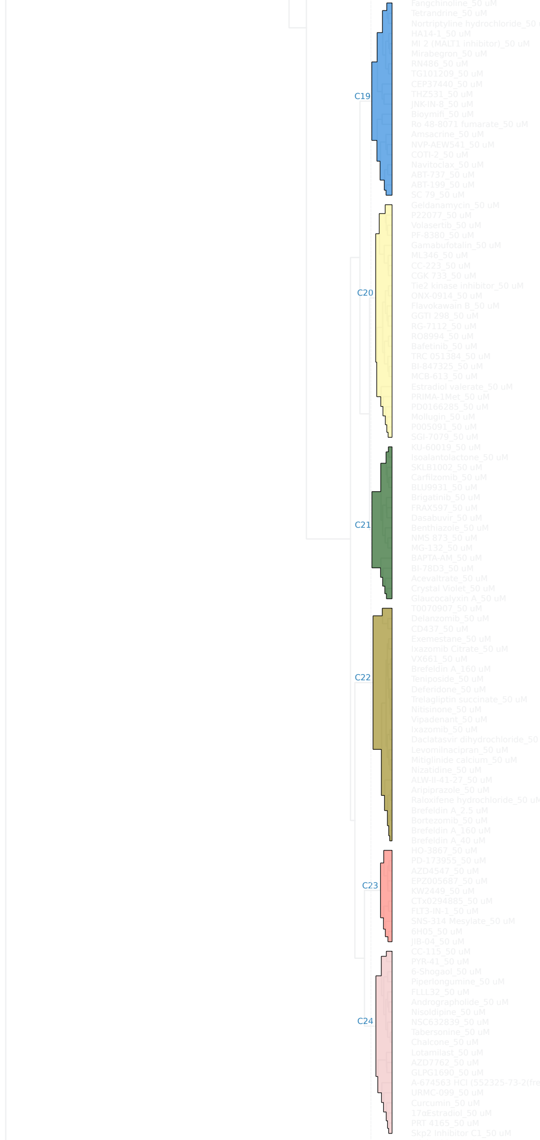

C19
C20
C21
C22
C23
C24
Fangchinoline_50 uM
Tetrandrine_50 uM
Nortriptyline hydrochloride_50
HA14-1_50 uM
MI 2 (MALT1 inhibitor)_50 uM
Mirabegron_50 uM
RN486_50 uM
TG101209_50 uM
CEP37440_50 uM
THZ531_50 uM
JNK-IN-8_50 uM
Bioymifi_50 uM
Ro 48-8071 fumarate_50 uM
Amsacrine_50 uM
NVP-AEW541_50 uM
COTI-2_50 uM
Navitoclax_50 uM
ABT-737_50 uM
ABT-199_50 uM
SC 79_50 uM
Geldanamycin_50 uM
P22077_50 uM
Volasertib_50 uM
PF-8380_50 uM
Gamabufotalin_50 uM
ML346_50 uM
CC-223_50 uM
CGK 733_50 uM
Tie2 kinase inhibitor_50 uM
ONX-0914_50 uM
Flavokawain B_50 uM
GGTI 298_50 uM
RG-7112_50 uM
RO8994_50 uM
Bafetinib_50 uM
TRC 051384_50 uM
BI-847325_50 uM
MCB-613_50 uM
Estradiol valerate_50 uM
PRIMA-1Met_50 uM
PD0166285_50 uM
Mollugin_50 uM
P005091_50 uM
SGI-7079_50 uM
KU-60019_50 uM
Isoalantolactone_50 uM
SKLB1002_50 uM
Carfilzomib_50 uM
BLU9931_50 uM
Brigatinib_50 uM
FRAX597_50 uM
Dasabuvir_50 uM
Benthiazole_50 uM
NMS 873_50 uM
MG-132_50 uM
BAPTA-AM_50 uM
BI-78D3_50 uM
Acevaltrate_50 uM
Crystal Violet_50 uM
Glaucocalyxin A_50 uM
T0070907_50 uM
Delanzomib_50 uM
CD437_50 uM
Exemestane_50 uM
Ixazomib Citrate_50 uM
VX661_50 uM
Brefeldin A_160 uM
Teniposide_50 uM
Deferidone_50 uM
Trelagliptin succinate_50 uM
Nitisinone_50 uM
Vipadenant_50 uM
Ixazomib_50 uM
Daclatasvir dihydrochloride_50
Levomilnacipran_50 uM
Mitiglinide calcium_50 uM
Nizatidine_50 uM
ALW-II-41-27_50 uM
Aripiprazole_50 uM
Raloxifene hydrochloride_50 uM
Brefeldin A_2.5 uM
Bortezomib_50 uM
Brefeldin A_160 uM
Brefeldin A_40 uM
HO-3867_50 uM
PD-173955_50 uM
AZD4547_50 uM
EPZ005687_50 uM
KW2449_50 uM
CTx0294885_50 uM
FLT3-IN-1_50 uM
SNS-314 Mesylate_50 uM
6H05_50 uM
JIB-04_50 uM
CC-115_50 uM
PYR-41_50 uM
6-Shogaol_50 uM
Piperlongumine_50 uM
FLLL32_50 uM
Andrographolide_50 uM
Nisoldipine_50 uM
NSC632839_50 uM
Tabersonine_50 uM
Chalcone_50 uM
Lotamilast_50 uM
AZD7762_50 uM
GLPG1690_50 uM
A-674563 HCl (552325-73-2(fre
URMC-099_50 uM
Curcumin_50 uM
17αEstradiol_50 uM
PRT 4165_50 uM
Skp2 Inhibitor C1_50 uM

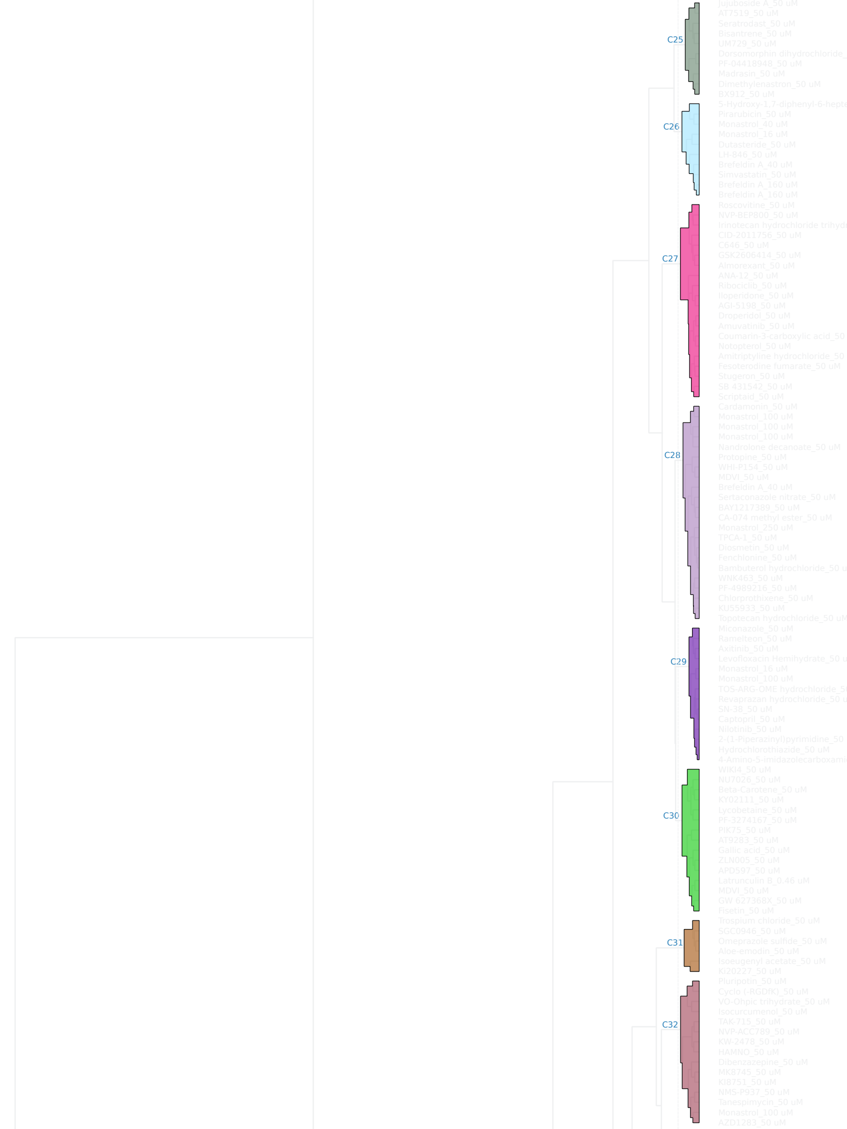
C25
C26
C27
C28
C29
C30
C31
C32
Jujuboside A_50 uM
AT7519_50 uM
Seratrodast_50 uM
Bisantrene_50 uM
UM729_50 uM
Dorsomorphin dihydrochloride_
PF-04418948_50 uM
Madrasin_50 uM
Dimethylenastron_50 uM
BX912_50 uM
5-Hydroxy-1,7-diphenyl-6-hepte
Pirarubicin_50 uM
Monastrol_40 uM
Monastrol_16 uM
Dutasteride_50 uM
LH-846_50 uM
Brefeldin A_40 uM
Simvastatin_50 uM
Brefeldin A_160 uM
Brefeldin A_160 uM
Roscovitine_50 uM
NVP-BEP800_50 uM
Irinotecan hydrochloride trihydr
CID-2011756_50 uM
C646_50 uM
GSK2606414_50 uM
Almorexant_50 uM
ANA-12_50 uM
Ribociclib_50 uM
Iloperidone_50 uM
AGI-5198_50 uM
Droperidol_50 uM
Amuvatinib_50 uM
Coumarin-3-carboxylic acid_50
Notopterol_50 uM
Amitriptyline hydrochloride_50
Fesoterodine fumarate_50 uM
Stugeron_50 uM
SB 431542_50 uM
Scriptaid_50 uM
Cardamonin_50 uM
Monastrol_100 uM
Monastrol_100 uM
Monastrol_100 uM
Nandrolone decanoate_50 uM
Protopine_50 uM
WHI-P154_50 uM
MDVI_50 uM
Brefeldin A_40 uM
Sertaconazole nitrate_50 uM
BAY1217389_50 uM
CA-074 methyl ester_50 uM
Monastrol_250 uM
TPCA-1_50 uM
Diosmetin_50 uM
Fenchlonine_50 uM
Bambuterol hydrochloride_50 u
WNK463_50 uM
PF-4989216_50 uM
Chlorprothixene_50 uM
KU55933_50 uM
Topotecan hydrochloride_50 uM
Miconazole_50 uM
Ramelteon_50 uM
Axitinib_50 uM
Levofloxacin Hemihydrate_50 u
Monastrol_16 uM
Monastrol_100 uM
TOS-ARG-OME hydrochloride_5
Revaprazan hydrochloride_50 u
SN-38_50 uM
Captopril_50 uM
Nilotinib_50 uM
2-(1-Piperazinyl)pyrimidine_50
Hydrochlorothiazide_50 uM
4-Amino-5-imidazolecarboxami
WIKI4_50 uM
NU7026_50 uM
Beta-Carotene_50 uM
KY02111_50 uM
Lycobetaine_50 uM
PF-3274167_50 uM
PIK75_50 uM
AT9283_50 uM
Gallic acid_50 uM
ZLN005_50 uM
APD597_50 uM
Latrunculin B_0.46 uM
MDVI_50 uM
GW 627368X_50 uM
Fisetin_50 uM
Trospium chloride_50 uM
SGC0946_50 uM
Omeprazole sulfide_50 uM
Aloe-emodin_50 uM
Isoeugenyl acetate_50 uM
Ki20227_50 uM
Pluripotin_50 uM
Cyclo (-RGDfK)_50 uM
VO-Ohpic trihydrate_50 uM
Isocurcumenol_50 uM
TAK-715_50 uM
NVP-ACC789_50 uM
KW-2478_50 uM
HAMNO_50 uM
Dibenzazepine_50 uM
MK8745_50 uM
KI8751_50 uM
NMS-P937_50 uM
Tanespimycin_50 uM
Monastrol_100 uM
AZD1283_50 uM

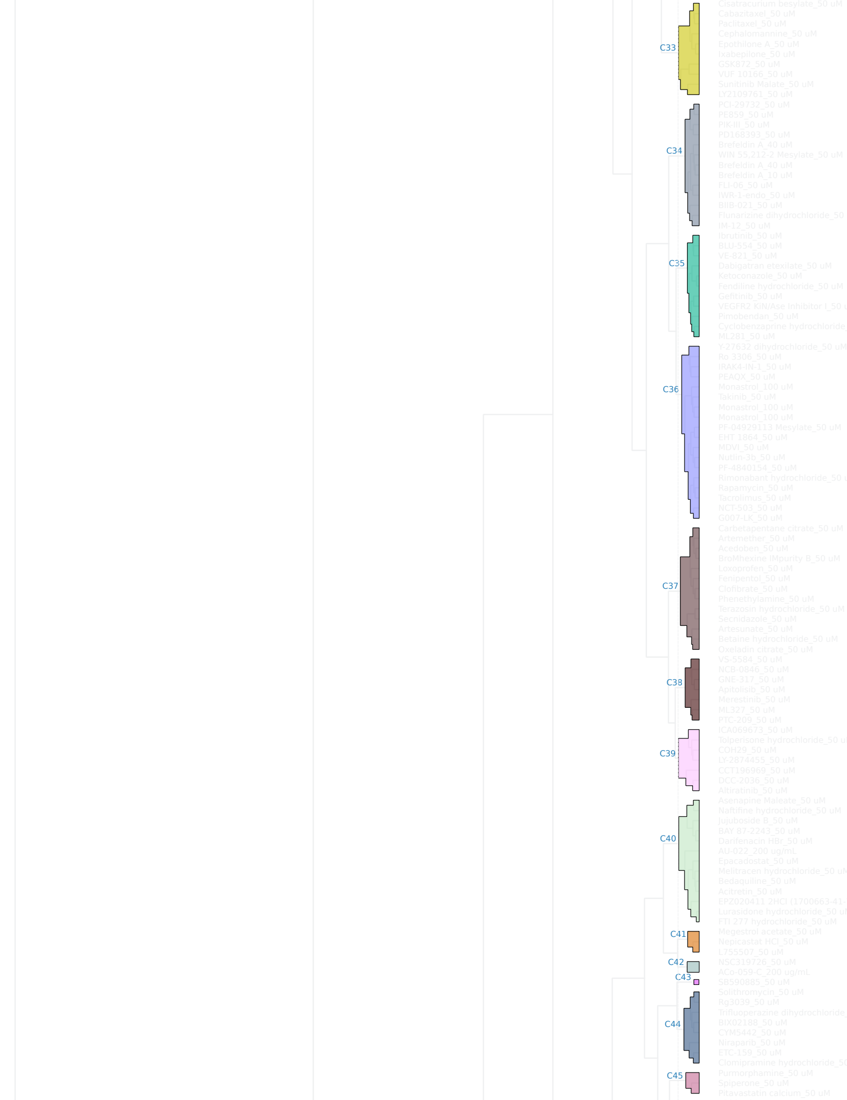
Cisatracurium besylate_50 uM
Cabazitaxel_50 uM
Paclitaxel_50 uM
Cephalomannine_50 uM
Epothilone A_50 uM
Ixabepilone_50 uM
GSK872_50 uM
VUF 10166_50 uM
Sunitinib Malate_50 uM
LY2109761_50 uM
PCI-29732_50 uM
PE859_50 uM
PIK-III_50 uM
PD168393_50 uM
Brefeldin A_40 uM
WIN 55,212-2 Mesylate_50 uM
Brefeldin A_40 uM
Brefeldin A_10 uM
FLI-06_50 uM
IWR-1-endo_50 uM
BIIB-021_50 uM
IM-12_50 uM
Ibrutinib_50 uM
BLU-554_50 uM
VE-821_50 uM
Dabigatran etexilate_50 uM
Ketoconazole_50 uM
Fendiline hydrochloride_50 uM
Gefitinib_50 uM
Pimobendan_50 uM
ML281_50 uM
Y-27632 dihydrochloride_50 uM
Ro 3306_50 uM
IRAK4-IN-1_50 uM
PEAQX_50 uM
Monastrol_100 uM
Takinib_50 uM
Monastrol_100 uM
Monastrol_100 uM
PF-04929113 Mesylate_50 uM
EHT 1864_50 uM
MDVI_50 uM
Nutlin-3b_50 uM
PF-4840154_50 uM
Rapamycin_50 uM
Tacrolimus_50 uM
NCT-503_50 uM
G007-LK_50 uM
Carbetapentane citrate_50 uM
Artemether_50 uM
Acedoben_50 uM
BroMhexine IMpurity B_50 uM
Loxoprofen_50 uM
Fenipentol_50 uM
Clofibrate_50 uM
Phenethylamine_50 uM
Terazosin hydrochloride_50 uM
Secnidazole_50 uM
Artesunate_50 uM
Betaine hydrochloride_50 uM
Oxeladin citrate_50 uM
VS-5584_50 uM
NCB-0846_50 uM
GNE-317_50 uM
Apitolisib_50 uM
Merestinib_50 uM
ML327_50 uM
PTC-209_50 uM
ICA069673_50 uM
COH29_50 uM
LY-2874455_50 uM
CCT196969_50 uM
DCC-2036_50 uM
Altiratinib_50 uM
Asenapine Maleate_50 uM
Naftifine hydrochloride_50 uM
Jujuboside B_50 uM
BAY 87-2243_50 uM
Darifenacin HBr_50 uM
AU-022_200 ug/mL
Epacadostat_50 uM
Bedaquiline_50 uM
Acitretin_50 uM
FTI 277 hydrochloride_50 uM
Megestrol acetate_50 uM
Nepicastat HCl_50 uM
L755507_50 uM
NSC319726_50 uM
ACo-059-C_200 ug/mL
SB590885_50 uM
Solithromycin_50 uM
Rg3039_50 uM
BIX02188_50 uM
CYM5442_50 uM
Niraparib_50 uM
ETC-159_50 uM
Purmorphamine_50 uM
Spiperone_50 uM
Pitavastatin calcium_50 uM
C33
C34
C35
C36
C37
C38
C39
C40
C41
C42
C43
C44
C45

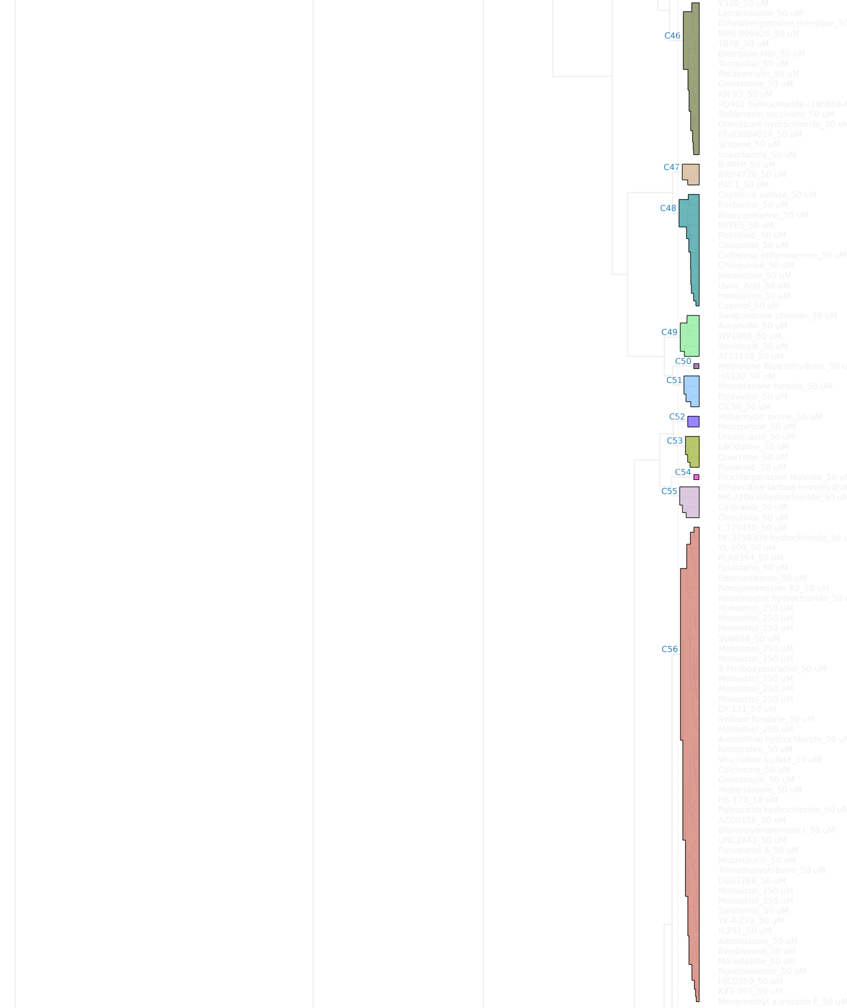
C46
C47
C48
C49
C50
C51
C52
C53
C54
C55
C56
Y320_50 uM
Lercanidipine_50 uM
Dihydroergotoxine mesylate_50
BMS 599626_50 uM
TBPB_50 uM
Eletriptan HBr_50 uM
Tariquidar_50 uM
Retapamulin_50 uM
Galeterone_50 uM
KN-93_50 uM
PQ401 hydrochloride (196868-6
Solifenacin succinate_50 uM
Otenabant hydrochloride_50 uM
PF-03084014_50 uM
Scopine_50 uM
Importazole_50 uM
R-IMPP_50 uM
BRD4770_50 uM
PAC1_50 uM
Coptisine sulfate_50 uM
Berberine_50 uM
Broxyquinoline_50 uM
BPTES_50 uM
Roblitinib_50 uM
Clioquinol_50 uM
Ciclopirox ethanolamine_50 uM
Chloquinan_50 uM
Nitroxoline_50 uM
Usnic Acid_50 uM
Iodoquinol_50 uM
Capitrol_50 uM
Sanguinarine chloride_50 uM
Auranofin_50 uM
WP1066_50 uM
Sonidegib_50 uM
AT13148_50 uM
Methylene Blue trihydrate_50 u
HA130_50 uM
Mometasone furoate_50 uM
Etravirine_50 uM
CIL56_50 uM
Milbemycin oxime_50 uM
Perospirone_50 uM
Ursolic acid_50 uM
Lacidipine_50 uM
Quercetin_50 uM
Ponatinib_50 uM
Prochlorperazine Maleate_50 u
Ethacridine lactate monohydrat
MK-2206 dihydrochloride_50 uM
Cediranib_50 uM
Olmutinib_50 uM
L-779450_50 uM
PF-3758309 hydrochloride_50 u
YL-109_50 uM
PLX8394_50 uM
Fatostatin_50 uM
Eburnalritardo_50 uM
Notoginsenoside R2_50 uM
Resminostat hydrochloride_50
Monastrol_250 uM
Monastrol_250 uM
Monastrol_250 uM
SU6656_50 uM
Monastrol_250 uM
Monastrol_250 uM
2-Methoxyestradiol_50 uM
Monastrol_250 uM
Monastrol_250 uM
Monastrol_250 uM
DY-131_50 uM
Sodium fusidate_50 uM
Monastrol_250 uM
Amorolfine hydrochloride_50 uM
Ketoprofen_50 uM
Vincristine sulfate_50 uM
Colchicine_50 uM
Ganetespib_50 uM
Mebendazole_50 uM
HS-173_50 uM
Palbociclib hydrochloride_50 uM
AZD0156_50 uM
Bisindolylmaleimide I_50 uM
UNC2881_50 uM
Purvalanol A_50 uM
Midostaurin_50 uM
Trimethoxystilbene_50 uM
DB07268_50 uM
Monastrol_250 uM
Monastrol_250 uM
Salubrinal_50 uM
YK-4-279_50 uM
IC261_50 uM
Albendazole_50 uM
Evodiamine_50 uM
Nocodazole_50 uM
Norethindrone_50 uM
HJC0350_50 uM
KX2-391_50 uM
Monomethyl auristatin E_50 uM

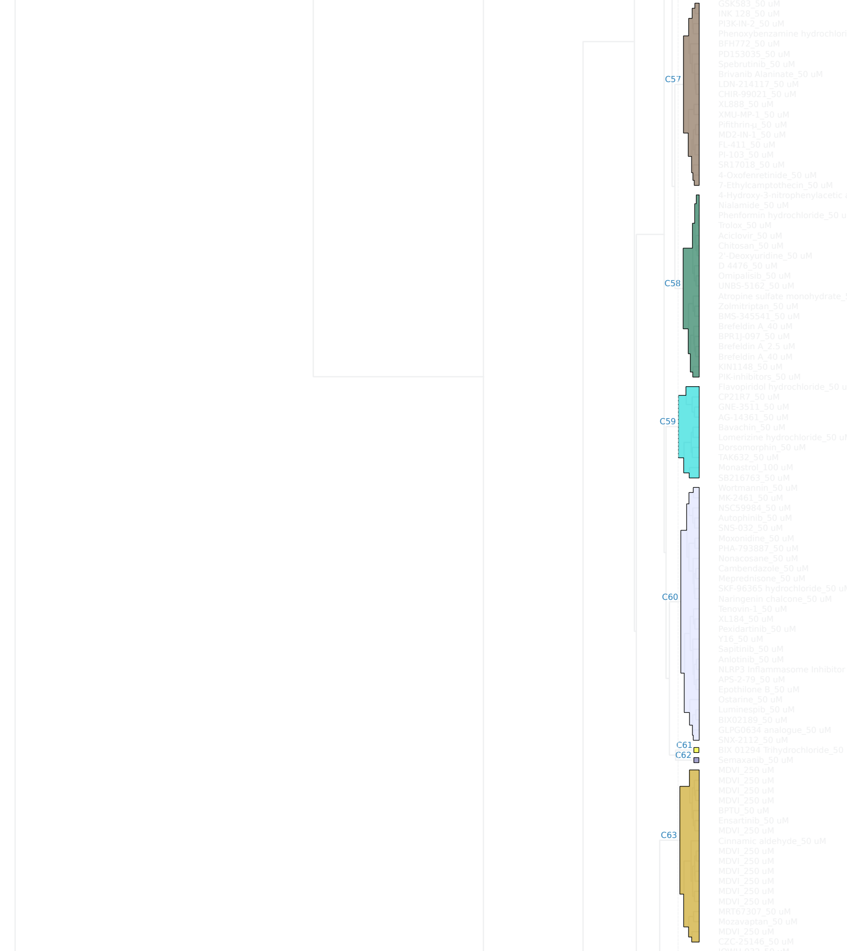
C57
C58
C59
C60
C61
C62
C63
GSK583_50 uM
INK 128_50 uM
PI3K-IN-2_50 uM
Phenoxybenzamine hydrochlori
BFH772_50 uM
PD153035_50 uM
Spebrutinib_50 uM
Brivanib Alaninate_50 uM
LDN-214117_50 uM
CHIR-99021_50 uM
XL888_50 uM
XMU-MP-1_50 uM
Pifithrin-μ_50 uM
MD2-IN-1_50 uM
FL-411_50 uM
PI-103_50 uM
SR17018_50 uM
4-Oxofenretinide_50 uM
7-Ethylcamptothecin_50 uM
4-Hydroxy-3-nitrophenylacetic a
Nialamide_50 uM
Phenformin hydrochloride_50 u
Trolox_50 uM
Aciclovir_50 uM
Chitosan_50 uM
2'-Deoxyuridine_50 uM
D 4476_50 uM
Omipalisib_50 uM
UNBS-5162_50 uM
Atropine sulfate monohydrate_
Zolmitriptan_50 uM
BMS-345541_50 uM
Brefeldin A_40 uM
BPR1J-097_50 uM
Brefeldin A_2.5 uM
Brefeldin A_40 uM
KIN1148_50 uM
PIK-inhibitors_50 uM
Flavopiridol hydrochloride_50 u
CP21R7_50 uM
GNE-3511_50 uM
AG-14361_50 uM
Bavachin_50 uM
Lomerizine hydrochloride_50 uM
Dorsomorphin_50 uM
TAK632_50 uM
Monastrol_100 uM
SB216763_50 uM
Wortmannin_50 uM
MK-2461_50 uM
NSC59984_50 uM
Autophinib_50 uM
SNS-032_50 uM
Moxonidine_50 uM
PHA-793887_50 uM
Nonacosane_50 uM
Cambendazole_50 uM
Meprednisone_50 uM
SKF-96365 hydrochloride_50 uM
Naringenin chalcone_50 uM
Tenovin-1_50 uM
XL184_50 uM
Pexidartinib_50 uM
Y16_50 uM
Sapitinib_50 uM
Anlotinib_50 uM
NLRP3 Inflammasome Inhibitor
APS-2-79_50 uM
Epothilone B_50 uM
Ostarine_50 uM
Luminespib_50 uM
BIX02189_50 uM
GLPG0634 analogue_50 uM
SNX-2112_50 uM
BIX 01294 Trihydrochloride_50
Semaxanib_50 uM
MDVI_250 uM
MDVI_250 uM
MDVI_250 uM
MDVI_250 uM
BPTU_50 uM
Ensartinib_50 uM
MDVI_250 uM
Cinnamic aldehyde_50 uM
MDVI_250 uM
MDVI_250 uM
MDVI_250 uM
MDVI_250 uM
MDVI_250 uM
MDVI_250 uM
MRT67307_50 uM
Mozavaptan_50 uM
MDVI_250 uM
CZC-25146_50 uM

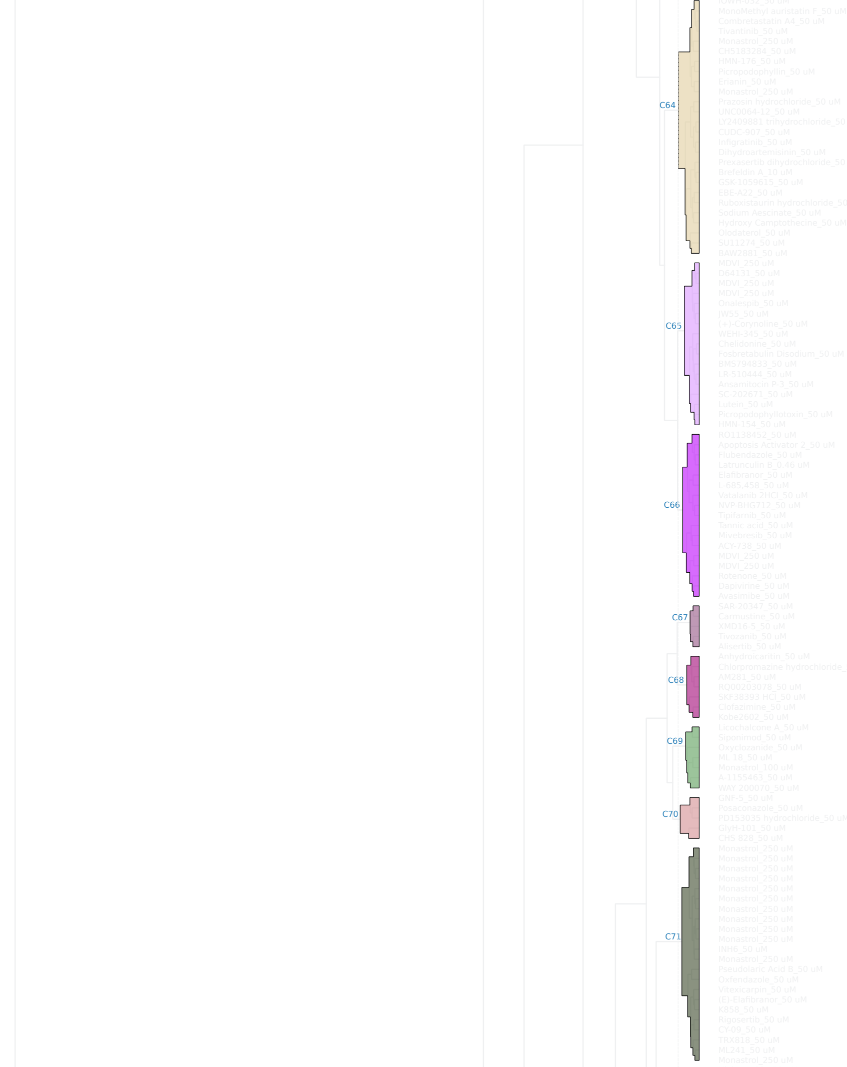

MonoMethyl auristatin F_50 uM
Combretastatin A4_50 uM
Tivantinib_50 uM
Monastrol_250 uM
CH5183284_50 uM
HMN-176_50 uM
Picropodophyllin_50 uM
Erianin_50 uM
Monastrol_250 uM
Prazosin hydrochloride_50 uM
UNC0064-12_50 uM
LY2409881 trihydrochloride_50
CUDC-907_50 uM
Infigratinib_50 uM
Dihydroartemisinin_50 uM
Prexasertib dihydrochloride_50
Brefeldin A_10 uM
GSK-1059615_50 uM
EBE-A22_50 uM
Ruboxistaurin hydrochloride_50
Sodium Aescinate_50 uM
Hydroxy Camptothecine_50 uM
Olodaterol_50 uM
SU11274_50 uM
BAW2881_50 uM
MDVI_250 uM
D64131_50 uM
MDVI_250 uM
MDVI_250 uM
Onalespib_50 uM
JW55_50 uM
(+)-Corynoline_50 uM
WEHI-345_50 uM
Chelidonine_50 uM
Fosbretabulin Disodium_50 uM
BMS794833_50 uM
LR-510444_50 uM
Ansamitocin P-3_50 uM
SC-202671_50 uM
Lutein_50 uM
Picropodophyllotoxin_50 uM
HMN-154_50 uM
RO1138452_50 uM
Apoptosis Activator 2_50 uM
Flubendazole_50 uM
Latrunculin B_0.46 uM
Elafibranor_50 uM
L-685,458_50 uM
Vatalanib 2HCl_50 uM
NVP-BHG712_50 uM
Tipifarnib_50 uM
Tannic acid_50 uM
Mivebresib_50 uM
ACY-738_50 uM
MDVI_250 uM
MDVI_250 uM
Rotenone_50 uM
Dapivirine_50 uM
Avasimibe_50 uM
SAR-20347_50 uM
Carmustine_50 uM
XMD16-5_50 uM
Tivozanib_50 uM
Alisertib_50 uM
Anhydroicaritin_50 uM
Chlorpromazine hydrochloride_
AM281_50 uM
RQ00203078_50 uM
SKF38393 HCl_50 uM
Clofazimine_50 uM
Kobe2602_50 uM
Licochalcone A_50 uM
Siponimod_50 uM
Oxyclozanide_50 uM
ML 18_50 uM
Monastrol_100 uM
A-1155463_50 uM
WAY 200070_50 uM
GNF-5_50 uM
Posaconazole_50 uM
PD153035 hydrochloride_50 u
GlyH-101_50 uM
CHS 828_50 uM
Monastrol_250 uM
Monastrol_250 uM
Monastrol_250 uM
Monastrol_250 uM
Monastrol_250 uM
Monastrol_250 uM
Monastrol_250 uM
Monastrol_250 uM
Monastrol_250 uM
Monastrol_250 uM
INH6_50 uM
Monastrol_250 uM
Pseudolaric Acid B_50 uM
Oxfendazole_50 uM
Vitexicarpin_50 uM
(E)-Elafibranor_50 uM
K858_50 uM
Rigosertib_50 uM
CY-09_50 uM
TRX818_50 uM
ML241_50 uM
Monastrol_250 uM
C64
C65
C66
C67
C68
C69
C70
C71

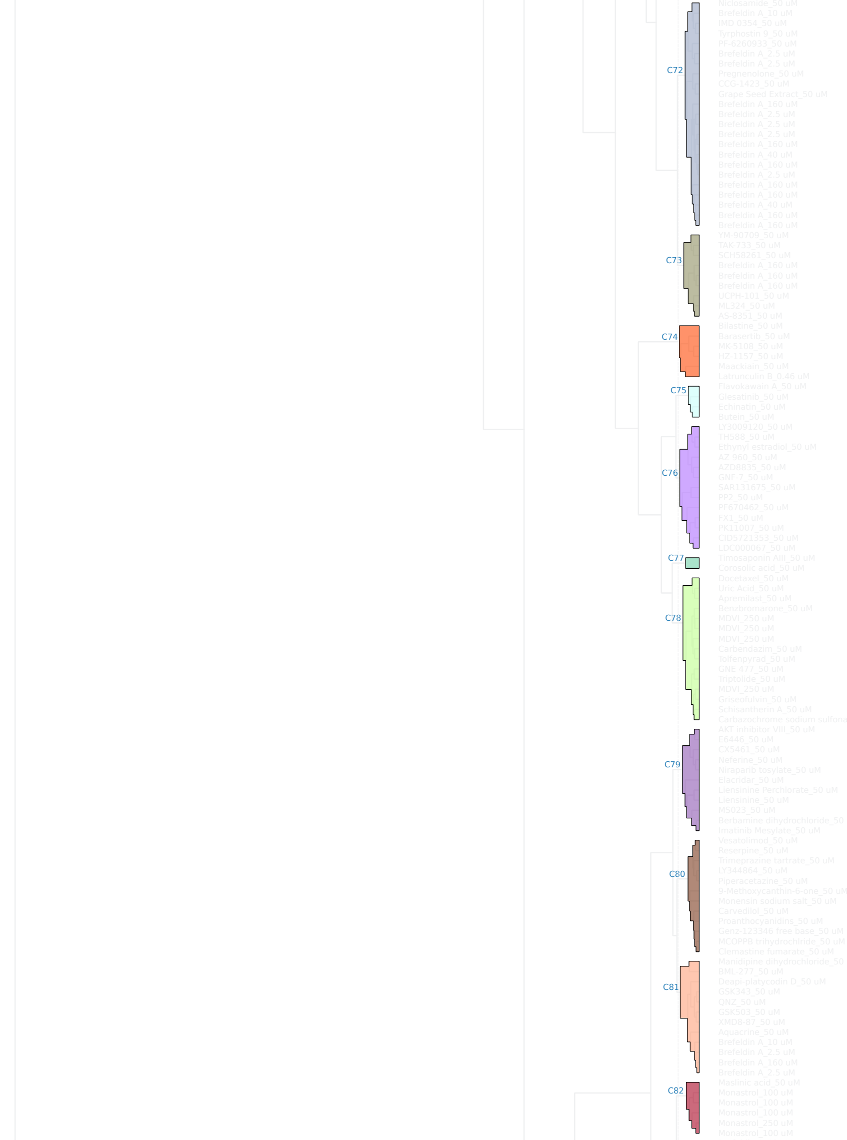

C72
C73
C74
C75
C76
C77
C78
C79
C80
C81
C82
Niclosamide_50 uM
Brefeldin A_10 uM
IMD 0354_50 uM
Tyrphostin 9_50 uM
PF-6260933_50 uM
Brefeldin A_2.5 uM
Brefeldin A_2.5 uM
Pregnenolone_50 uM
CCG-1423_50 uM
Grape Seed Extract_50 uM
Brefeldin A_160 uM
Brefeldin A_2.5 uM
Brefeldin A_2.5 uM
Brefeldin A_2.5 uM
Brefeldin A_160 uM
Brefeldin A_40 uM
Brefeldin A_160 uM
Brefeldin A_2.5 uM
Brefeldin A_160 uM
Brefeldin A_160 uM
Brefeldin A_40 uM
Brefeldin A_160 uM
Brefeldin A_160 uM
YM-90709_50 uM
TAK-733_50 uM
SCH58261_50 uM
Brefeldin A_160 uM
Brefeldin A_160 uM
Brefeldin A_160 uM
UCPH-101_50 uM
ML324_50 uM
AS-8351_50 uM
Bilastine_50 uM
Barasertib_50 uM
MK-5108_50 uM
HZ-1157_50 uM
Maackiain_50 uM
Latrunculin B_0.46 uM
Flavokawain A_50 uM
Glesatinib_50 uM
Echinatin_50 uM
Butein_50 uM
LY3009120_50 uM
TH588_50 uM
Ethynyl estradiol_50 uM
AZ 960_50 uM
AZD8835_50 uM
GNF-7_50 uM
SAR131675_50 uM
PP2_50 uM
PF670462_50 uM
FX1_50 uM
PK11007_50 uM
CID5721353_50 uM
LDC000067_50 uM
Timosaponin AIII_50 uM
Corosolic acid_50 uM
Docetaxel_50 uM
Uric Acid_50 uM
Apremilast_50 uM
Benzbromarone_50 uM
MDVI_250 uM
MDVI_250 uM
MDVI_250 uM
Carbendazim_50 uM
Tolfenpyrad_50 uM
GNE 477_50 uM
Triptolide_50 uM
MDVI_250 uM
Griseofulvin_50 uM
Schisantherin A_50 uM
Carbazochrome sodium sulfona
AKT inhibitor VIII_50 uM
E6446_50 uM
CX5461_50 uM
Neferine_50 uM
Niraparib tosylate_50 uM
Elacridar_50 uM
Liensinine Perchlorate_50 uM
Liensinine_50 uM
MS023_50 uM
Berbamine dihydrochloride_50
Imatinib Mesylate_50 uM
Vesatolimod_50 uM
Reserpine_50 uM
Trimeprazine tartrate_50 uM
LY344864_50 uM
Piperacetazine_50 uM
9-Methoxycanthin-6-one_50 uM
Monensin sodium salt_50 uM
Carvedilol_50 uM
Proanthocyanidins_50 uM
Genz-123346 free base_50 uM
MCOPPB trihydrochlride_50 uM
Clemastine fumarate_50 uM
Manidipine dihydrochloride_50
BML-277_50 uM
Deapi-platycodin D_50 uM
GSK343_50 uM
QNZ_50 uM
GSK503_50 uM
XMD8-87_50 uM
Aquacrine_50 uM
Brefeldin A_10 uM
Brefeldin A_2.5 uM
Brefeldin A_160 uM
Brefeldin A_2.5 uM
Maslinic acid_50 uM
Monastrol_100 uM
Monastrol_100 uM
Monastrol_100 uM
Monastrol_250 uM
Monastrol_100 uM

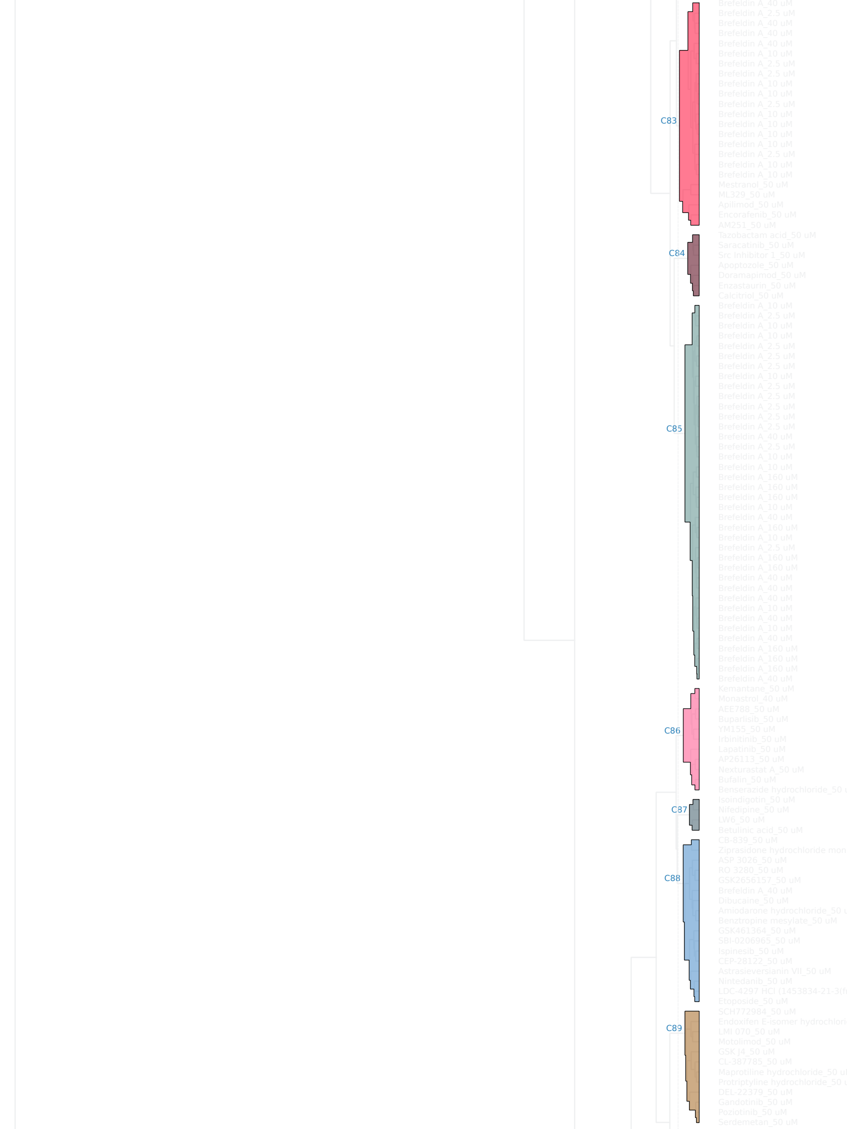
C83
C84
C85
C86
C87
C88
C89
Brefeldin A_40 uM
Brefeldin A_2.5 uM
Brefeldin A_40 uM
Brefeldin A_40 uM
Brefeldin A_40 uM
Brefeldin A_10 uM
Brefeldin A_2.5 uM
Brefeldin A_2.5 uM
Brefeldin A_10 uM
Brefeldin A_10 uM
Brefeldin A_2.5 uM
Brefeldin A_10 uM
Brefeldin A_10 uM
Brefeldin A_10 uM
Brefeldin A_10 uM
Brefeldin A_2.5 uM
Brefeldin A_10 uM
Brefeldin A_10 uM
Mestranol_50 uM
ML329_50 uM
Apilimod_50 uM
Encorafenib_50 uM
AM251_50 uM
Tazobactam acid_50 uM
Saracatinib_50 uM
Src Inhibitor 1_50 uM
Apoptozole_50 uM
Doramapimod_50 uM
Enzastaurin_50 uM
Calcitriol_50 uM
Brefeldin A_10 uM
Brefeldin A_2.5 uM
Brefeldin A_10 uM
Brefeldin A_10 uM
Brefeldin A_2.5 uM
Brefeldin A_2.5 uM
Brefeldin A_2.5 uM
Brefeldin A_10 uM
Brefeldin A_2.5 uM
Brefeldin A_2.5 uM
Brefeldin A_2.5 uM
Brefeldin A_2.5 uM
Brefeldin A_2.5 uM
Brefeldin A_40 uM
Brefeldin A_2.5 uM
Brefeldin A_10 uM
Brefeldin A_10 uM
Brefeldin A_160 uM
Brefeldin A_160 uM
Brefeldin A_160 uM
Brefeldin A_10 uM
Brefeldin A_40 uM
Brefeldin A_160 uM
Brefeldin A_10 uM
Brefeldin A_2.5 uM
Brefeldin A_160 uM
Brefeldin A_160 uM
Brefeldin A_40 uM
Brefeldin A_40 uM
Brefeldin A_40 uM
Brefeldin A_10 uM
Brefeldin A_40 uM
Brefeldin A_10 uM
Brefeldin A_40 uM
Brefeldin A_160 uM
Brefeldin A_160 uM
Brefeldin A_160 uM
Brefeldin A_40 uM
Kemantane_50 uM
Monastrol_40 uM
AEE788_50 uM
Buparlisib_50 uM
YM155_50 uM
Irbinitinib_50 uM
Lapatinib_50 uM
AP26113_50 uM
Nexturastat A_50 uM
Bufalin_50 uM
Benserazide hydrochloride_50 u
Isoindigotin_50 uM
Nifedipine_50 uM
LW6_50 uM
Betulinic acid_50 uM
CB-839_50 uM
Ziprasidone hydrochloride mon
ASP 3026_50 uM
RO 3280_50 uM
GSK2656157_50 uM
Brefeldin A_40 uM
Dibucaine_50 uM
Amiodarone hydrochloride_50 u
Benztropine mesylate_50 uM
GSK461364_50 uM
SBI-0206965_50 uM
Ispinesib_50 uM
CEP-28122_50 uM
Astrasieversianin VII_50 uM
Nintedanib_50 uM
LDC-4297 HCl (1453834-21-3(f
Etoposide_50 uM
SCH772984_50 uM
Endoxifen E-isomer hydrochlori
LMI 070_50 uM
Motolimod_50 uM
GSK J4_50 uM
CL-387785_50 uM
Maprotiline hydrochloride_50 u
Protriptyline hydrochloride_50 u
DEL-22379_50 uM
Gandotinib_50 uM
Poziotinib_50 uM
Serdemetan_50 uM

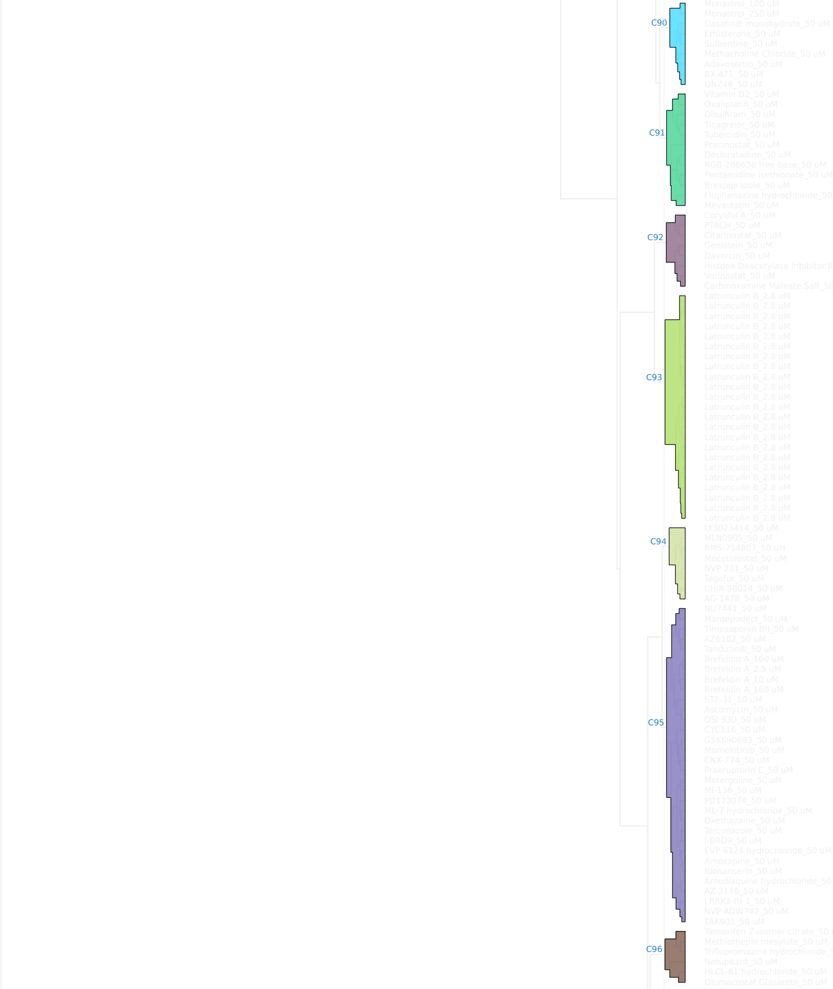
C90
C91
C92
C93
C94
C95
C96
Monastrol_100 uM
Monastrol_250 uM
Dasatinib monohydrate_50 uM
Ethisterone_50 uM
Sulbentine_50 uM
Methacholine Chloride_50 uM
Adavosertib_50 uM
BX-471_50 uM
QNZ46_50 uM
Vitamin D2_50 uM
Oxaliplatin_50 uM
Disulfiram_50 uM
Ticagrelor_50 uM
Tubercidin_50 uM
Pracinostat_50 uM
Desloratadine_50 uM
RGB-286638 free base_50 uM
Pentamidine isethionate_50 uM
Brexpiprazole_50 uM
Mevastatin_50 uM
Corylifol A_50 uM
PTACH_50 uM
Citarinostat_50 uM
Genistein_50 uM
Davercin_50 uM
Vorinostat_50 uM
Latrunculin B_2.8 uM
LY3023414_50 uM
MLN0905_50 uM
BMS-754807_50 uM
Mocetinostat_50 uM
NVP 231_50 uM
Tegafur_50 uM
CHIR-98014_50 uM
AG-1478_50 uM
NU7441_50 uM
Mardepodect_50 uM
Timosaponin BII_50 uM
AZ6102_50 uM
Tandutinib_50 uM
Brefeldin A_160 uM
Brefeldin A_2.5 uM
Brefeldin A_10 uM
Brefeldin A_160 uM
STF-31_50 uM
Ascomycin_50 uM
OSI 930_50 uM
CYC116_50 uM
GSK690693_50 uM
Momelotinib_50 uM
CNX-774_50 uM
Praeruptorin C_50 uM
Metergoline_50 uM
MI-136_50 uM
PD173074_50 uM
ML-7 hydrochloride_50 uM
Oxethazaine_50 uM
Terconazole_50 uM
I-BRD9_50 uM
EVP-6124 hydrochloride_50 uM
Amoxapine_50 uM
Blonanserin_50 uM
AZ 3146_50 uM
LRRK2-IN-1_50 uM
NVP-ADW742_50 uM
TAK901_50 uM
Methiothepin mesylate_50 uM
Netupitant_50 uM
HLCL-61 hydrochloride_50 uM
Olumacostat Glasaretil_50 uM

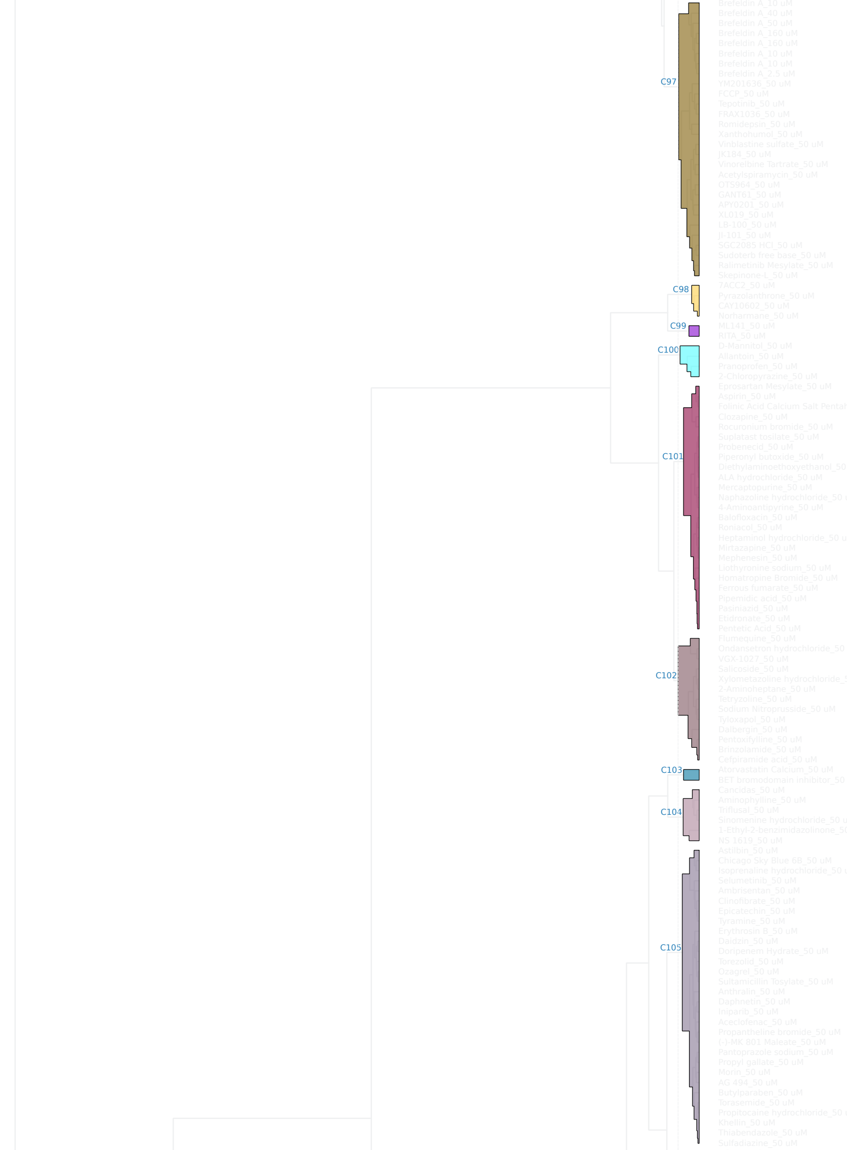

C97
C98
C99
C100
C101
C102
C103
C104
C105
Brefeldin A_10 uM
Brefeldin A_40 uM
Brefeldin A_50 uM
Brefeldin A_160 uM
Brefeldin A_160 uM
Brefeldin A_10 uM
Brefeldin A_10 uM
Brefeldin A_2.5 uM
YM201636_50 uM
FCCP_50 uM
Tepotinib_50 uM
FRAX1036_50 uM
Romidepsin_50 uM
Xanthohumol_50 uM
Vinblastine sulfate_50 uM
JK184_50 uM
Vinorelbine Tartrate_50 uM
Acetylspiramycin_50 uM
OTS964_50 uM
GANT61_50 uM
APY0201_50 uM
XL019_50 uM
LB-100_50 uM
JI-101_50 uM
SGC2085 HCl_50 uM
Sudoterb free base_50 uM
Ralimetinib Mesylate_50 uM
Skepinone-L_50 uM
7ACC2_50 uM
Pyrazolanthrone_50 uM
CAY10602_50 uM
Norharmane_50 uM
ML141_50 uM
RITA_50 uM
D-Mannitol_50 uM
Allantoin_50 uM
Pranoprofen_50 uM
2-Chloropyrazine_50 uM
Eprosartan Mesylate_50 uM
Aspirin_50 uM
Folinic Acid Calcium Salt Pentah
Clozapine_50 uM
Rocuronium bromide_50 uM
Suplatast tosilate_50 uM
Probenecid_50 uM
Piperonyl butoxide_50 uM
Diethylaminoethoxyethanol_50
ALA hydrochloride_50 uM
Mercaptopurine_50 uM
Naphazoline hydrochloride_50 u
4-Aminoantipyrine_50 uM
Balofloxacin_50 uM
Roniacol_50 uM
Heptaminol hydrochloride_50 u
Mirtazapine_50 uM
Mephenesin_50 uM
Liothyronine sodium_50 uM
Homatropine Bromide_50 uM
Ferrous fumarate_50 uM
Pipemidic acid_50 uM
Pasiniazid_50 uM
Etidronate_50 uM
Pentetic Acid_50 uM
Flumequine_50 uM
Ondansetron hydrochloride_50
VGX-1027_50 uM
Salicoside_50 uM
Xylometazoline hydrochloride_5
2-Aminoheptane_50 uM
Tetryzoline_50 uM
Sodium Nitroprusside_50 uM
Tyloxapol_50 uM
Dalbergin_50 uM
Pentoxifylline_50 uM
Brinzolamide_50 uM
Cefpiramide acid_50 uM
Atorvastatin Calcium_50 uM
BET bromodomain inhibitor_50
Cancidas_50 uM
Aminophylline_50 uM
Triflusal_50 uM
Sinomenine hydrochloride_50 u
1-Ethyl-2-benzimidazolinone_50
NS 1619_50 uM
Astilbin_50 uM
Chicago Sky Blue 6B_50 uM
Isoprenaline hydrochloride_50 u
Selumetinib_50 uM
Ambrisentan_50 uM
Clinofibrate_50 uM
Epicatechin_50 uM
Tyramine_50 uM
Erythrosin B_50 uM
Daidzin_50 uM
Doripenem Hydrate_50 uM
Torezolid_50 uM
Ozagrel_50 uM
Sultamicillin Tosylate_50 uM
Anthralin_50 uM
Daphnetin_50 uM
Iniparib_50 uM
Aceclofenac_50 uM
Propantheline bromide_50 uM
(-)-MK 801 Maleate_50 uM
Pantoprazole sodium_50 uM
Propyl gallate_50 uM
Morin_50 uM
AG 494_50 uM
Butylparaben_50 uM
Torasemide_50 uM
Propitocaine hydrochloride_50 u
Khellin_50 uM
Thiabendazole_50 uM
Sulfadiazine_50 uM

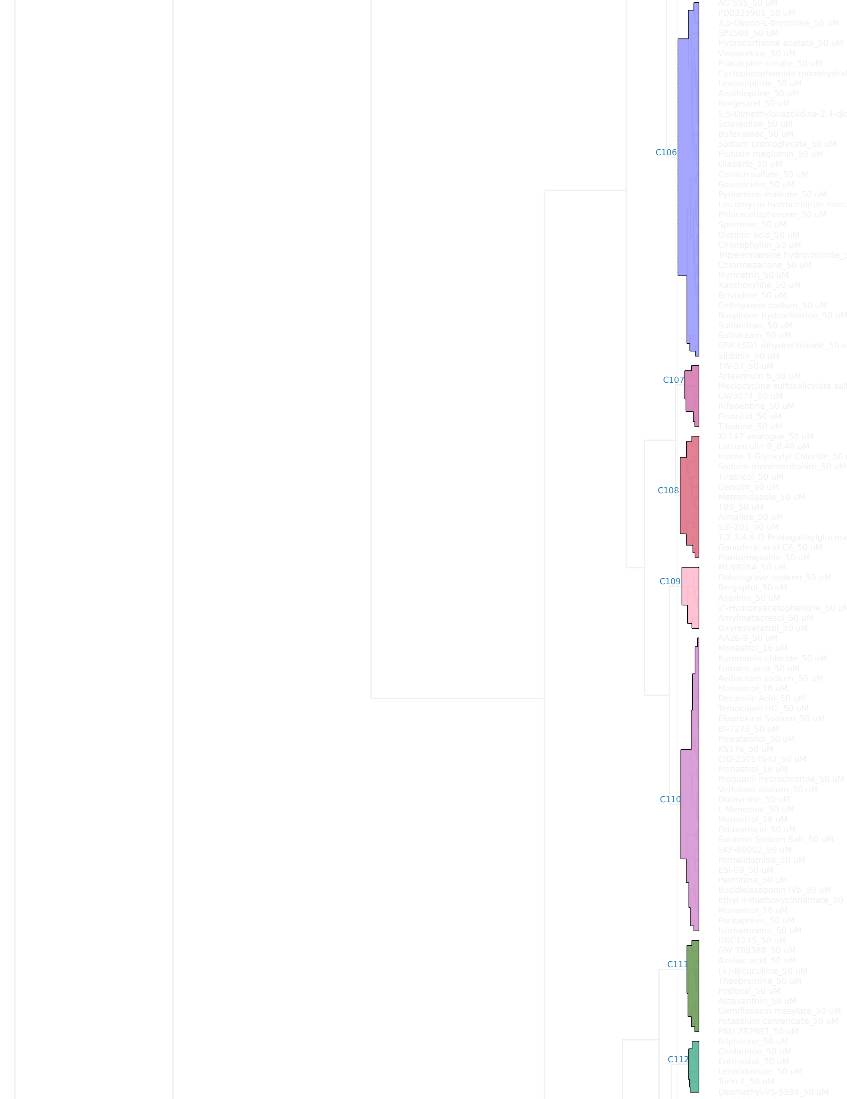
C106
C107
C108
C109
C110
C111
C112
AG 555_50 uM
PD0325901_50 uM
3,5-Diiodo-L-thyronine_50 uM
SP2509_50 uM
Hydrocortisone acetate_50 uM
Vinpocetine_50 uM
Pilocarpine nitrate_50 uM
Cyclophosphamide monohydrat
Levosulpiride_50 uM
Azathioprine_50 uM
Norgestrel_50 uM
5,5-Dimethyloxazolidine-2,4-dic
Sclareolide_50 uM
Bufexamac_50 uM
Sodium cromoglycate_50 uM
Flunixin meglumin_50 uM
Olaparib_50 uM
Colistin sulfate_50 uM
Bovinocidin_50 uM
Pyrilamine maleate_50 uM
Lincomycin hydrochloride mono
Phloracetophenone_50 uM
Spermine_50 uM
Oxolinic acid_50 uM
Chlorophyllin_50 uM
Tripelennamine hydrochloride_5
Chlormezanone_50 uM
Myricetrin_50 uM
Xanthoxyline_50 uM
Brivudine_50 uM
Ceftriaxone sodium_50 uM
Buspirone hydrochloride_50 uM
Sulfanitran_50 uM
Sulbactam_50 uM
GSK-LSD1 dihydrochloride_50 u
Silibinin_50 uM
TW-37_50 uM
Arteannuin B_50 uM
Meclocycline sulfosalicylate sal
GW5074_50 uM
Rifapentine_50 uM
Fluonilid_50 uM
Tibolone_50 uM
XL147 analogue_50 uM
Latrunculin B_0.46 uM
Indole-3-Glyoxylyl Chloride_50
Sodium montmorillonite_50 uM
Tiratricol_50 uM
Genipin_50 uM
Metronidazole_50 uM
TBB_50 uM
Ajmaline_50 uM
S3I-201_50 uM
1,2,3,4,6-O-Pentagalloylglucose
Ganoderic acid C6_50 uM
Plantamajoside_50 uM
MLN8054_50 uM
Dolutegravir sodium_50 uM
Bergaptol_50 uM
Asarinin_50 uM
2'-Hydroxyacetophenone_50 u
Amylmetacresol_50 uM
Oxyresveratrol_50 uM
AA26-9_50 uM
Monastrol_16 uM
Kuromanin chloride_50 uM
Fumaric acid_50 uM
Avibactam sodium_50 uM
Monastrol_16 uM
Decanoic Acid_50 uM
Temocapril HCl_50 uM
Efaproxiral Sodium_50 uM
BI-7273_50 uM
Piceatannol_50 uM
KS176_50 uM
CID-25014542_50 uM
Monastrol_16 uM
Proguanil hydrochloride_50 uM
Verlukast sodium_50 uM
Doravirine_50 uM
L-Mimosine_50 uM
Monastrol_16 uM
Fidaxomicin_50 uM
Suramin Sodium Salt_50 uM
SKF-86002_50 uM
Pomalidomide_50 uM
ESI-09_50 uM
Peiminine_50 uM
Buddlejasaponin IVb_50 uM
Ethyl 4-methoxycinnamate_50
Monastrol_16 uM
Paritaprevir_50 uM
Isorhamnetin_50 uM
UNC1215_50 uM
GW 788388_50 uM
Azelaic acid_50 uM
(+)-Bicuculline_50 uM
Theobromine_50 uM
Fosfosal_50 uM
Astaxanthin_50 uM
Gemifloxacin mesylate_50 uM
Potassium canrenoate_50 uM
PNU 282987_50 uM
Rilpivirine_50 uM
Chidamide_50 uM
Entinostat_50 uM
Lenalidomide_50 uM
Torin 1_50 uM
Desmethyl-VS-5584_50 uM

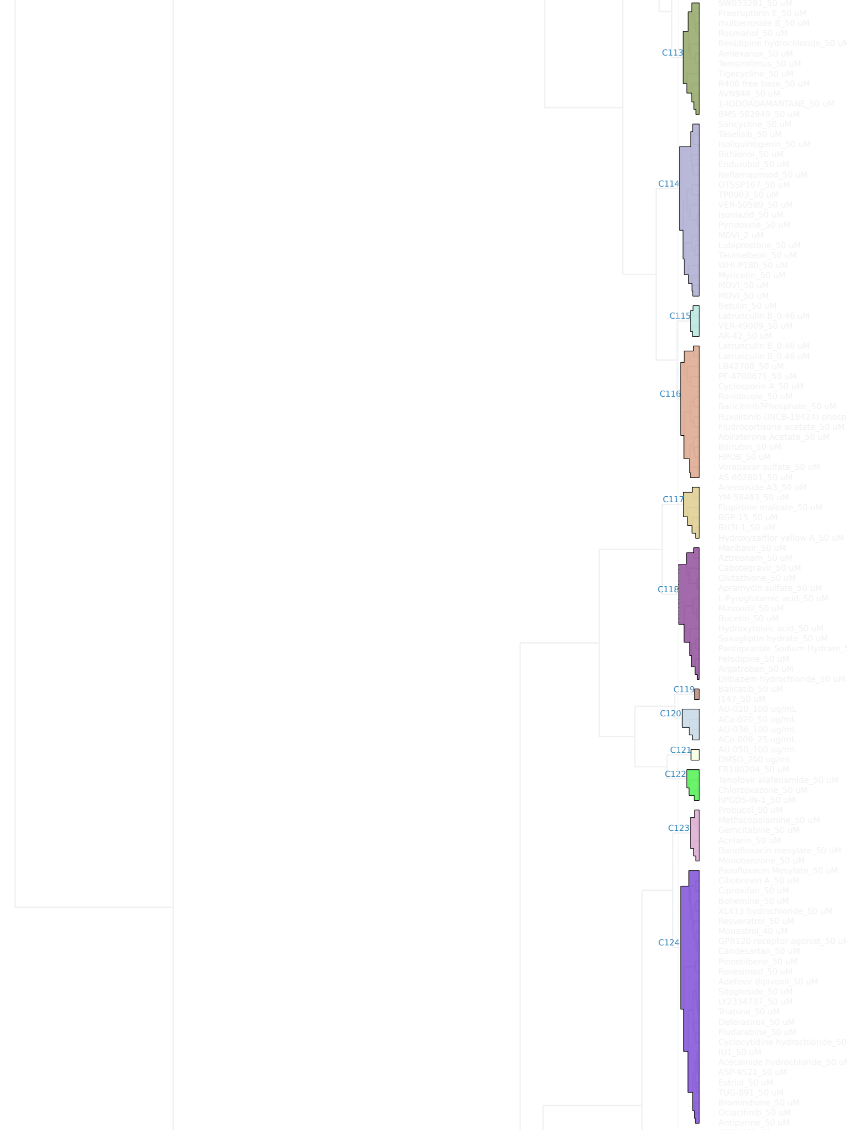
C113
C114
C115
C116
C117
C118
C119
C120
C121
C122
C123
C124
SW033291_50 uM
Praeruptorin E_50 uM
mulberroside B_50 uM
Rosmanol_50 uM
Benidipine hydrochloride_50 uM
Amlexanox_50 uM
Temsirolimus_50 uM
Tigecycline_50 uM
R406 free base_50 uM
AVN944_50 uM
1-IODOADAMANTANE_50 uM
BMS-582949_50 uM
Sancycline_50 uM
Taselisib_50 uM
Isoliquiritigenin_50 uM
Bithionol_50 uM
Endurobol_50 uM
Neflamapimod_50 uM
OTSSP167_50 uM
TP0903_50 uM
VER-50589_50 uM
Isoniazid_50 uM
Pyridoxine_50 uM
MDVI_2 uM
Lubiprostone_50 uM
Tasimelteon_50 uM
WHI-P180_50 uM
Myricetin_50 uM
MDVI_50 uM
MDVI_50 uM
Betulin_50 uM
Latrunculin B_0.46 uM
VER-49009_50 uM
AR-42_50 uM
Latrunculin B_0.46 uM
Latrunculin B_0.46 uM
LB42708_50 uM
PF-4708671_50 uM
Cyclosporin A_50 uM
Ronidazole_50 uM
Baricitinib?Phosphate_50 uM
Ruxolitinib (INCB-18424) phosp
Fludrocortisone acetate_50 uM
Abiraterone Acetate_50 uM
Bilirubin_50 uM
HPOB_50 uM
Vorapaxar sulfate_50 uM
AS 602801_50 uM
Anemoside A3_50 uM
YM-58483_50 uM
Flupirtine maleate_50 uM
BGP-15_50 uM
BH3I-1_50 uM
Hydroxysafflor yellow A_50 uM
Maribavir_50 uM
Aztreonam_50 uM
Cabotegravir_50 uM
Glutathione_50 uM
Apramycin sulfate_50 uM
L-Pyroglutamic acid_50 uM
Minoxidil_50 uM
Bucetin_50 uM
Hydroxytoluic acid_50 uM
Saxagliptin hydrate_50 uM
Pantoprazole Sodium Hydrate_
Felodipine_50 uM
Argatroban_50 uM
Diltiazem hydrochloride_50 uM
Balicatib_50 uM
J147_50 uM
AU-020_100 ug/mL
ACa-020_50 ug/mL
AU-036_100 ug/mL
ACo-009_25 ug/mL
AU-050_100 ug/mL
DMSO_200 ug/mL
FR180204_50 uM
Tenofovir alafenamide_50 uM
Chlorzoxazone_50 uM
hPGDS-IN-1_50 uM
Probucol_50 uM
Methscopolamine_50 uM
Gemcitabine_50 uM
Acelarin_50 uM
Danofloxacin mesylate_50 uM
Monobenzone_50 uM
Pazufloxacin Mesylate_50 uM
Ciliobrevin A_50 uM
Ciproxifan_50 uM
Bohemine_50 uM
XL413 hydrochloride_50 uM
Resveratrol_50 uM
Monastrol_40 uM
GPR120 receptor agonist_50 uM
Candesartan_50 uM
Pinostilbene_50 uM
Ponesimod_50 uM
Adefovir dipivoxil_50 uM
Sitogluside_50 uM
LY2334737_50 uM
Triapine_50 uM
Deferasirox_50 uM
Fludarabine_50 uM
Cyclocytidine hydrochloride_50
IU1_50 uM
Acecainide hydrochloride_50 uM
ASP-9521_50 uM
Estriol_50 uM
TUG-891_50 uM
Bromindione_50 uM
Oclacitinib_50 uM
Antipyrine_50 uM

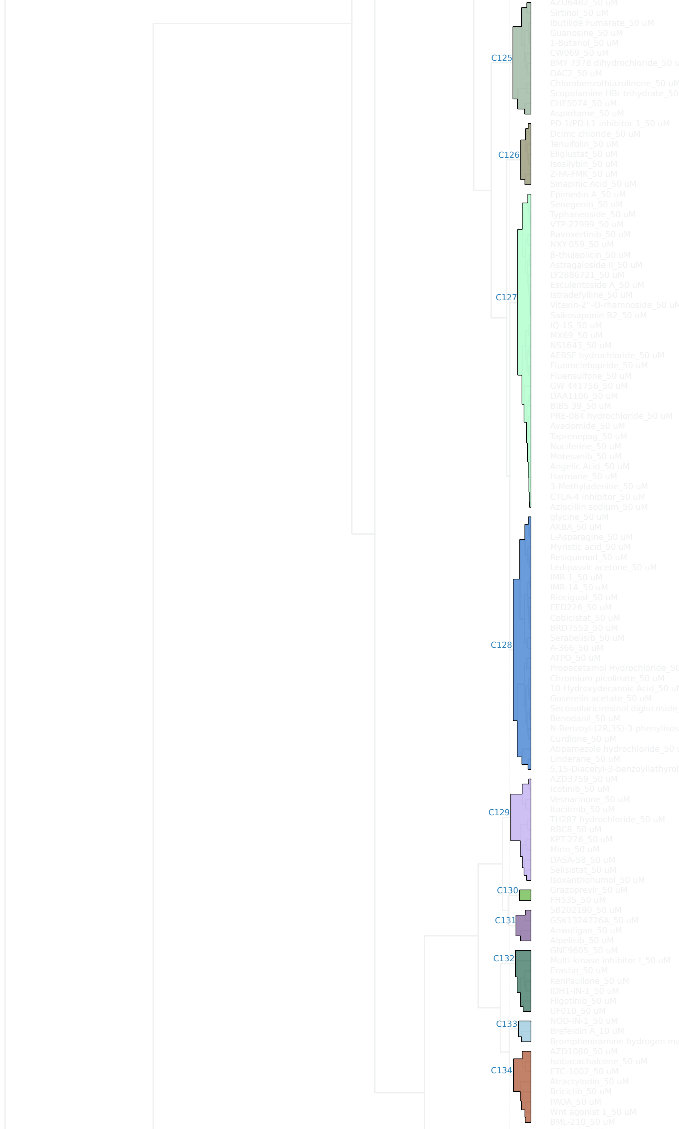
C125
C126
C127
C128
C129
C130
C131
C132
C133
C134
AZD6482_50 uM
Sirtinol_50 uM
Ibutilide Fumarate_50 uM
Guanosine_50 uM
1-Butanol_50 uM
CW069_50 uM
BMY 7378 dihydrochloride_50 u
OAC2_50 uM
Chlorobenzothiazolinone_50 uM
Scopolamine HBr trihydrate_50
CHF5074_50 uM
Aspartame_50 uM
PD-1/PD-L1 inhibitor 1_50 uM
Dcimc chloride_50 uM
Tenuifolin_50 uM
Eliglustat_50 uM
Isosilybin_50 uM
Z-FA-FMK_50 uM
Sinapinic Acid_50 uM
Epimedin A_50 uM
Senegenin_50 uM
Typhaneoside_50 uM
VTP-27999_50 uM
Ravoxertinib_50 uM
NXY-059_50 uM
β-thujaplicin_50 uM
Astragaloside II_50 uM
LY2886721_50 uM
Esculentoside A_50 uM
Istradefylline_50 uM
Vitexin-2''-O-rhamnoside_50 uM
Saikosaponin B2_50 uM
IQ-1S_50 uM
MX69_50 uM
NS1643_50 uM
AEBSF hydrochloride_50 uM
Fluoroclebopride_50 uM
Fluensulfone_50 uM
GW 441756_50 uM
DAA1106_50 uM
BIBS 39_50 uM
PRE-084 hydrochloride_50 uM
Avadomide_50 uM
Taprenepag_50 uM
Nuciferine_50 uM
Motesanib_50 uM
Angelic Acid_50 uM
Harmane_50 uM
3-Methyladenine_50 uM
CTLA-4 inhibitor_50 uM
Azlocillin sodium_50 uM
glycine_50 uM
AKBA_50 uM
L-Asparagine_50 uM
Myristic acid_50 uM
Resiquimod_50 uM
Ledipasvir acetone_50 uM
IMR-1_50 uM
IMR-1A_50 uM
Riociguat_50 uM
EED226_50 uM
Cobicistat_50 uM
BRD7552_50 uM
Serabelisib_50 uM
A-366_50 uM
ATPO_50 uM
Propacetamol Hydrochloride_50
Chromium picolinate_50 uM
10-Hydroxydecanoic Acid_50 u
Goserelin acetate_50 uM
Secoisolariciresinol diglucoside_
Benodanil_50 uM
N-Benzoyl-(2R,3S)-3-phenylisos
Curdione_50 uM
Atipamezole hydrochloride_50
Linderane_50 uM
5,15-Diacetyl-3-benzoyllathyrol
AZD3759_50 uM
Icotinib_50 uM
Vesnarinone_50 uM
Itacitinib_50 uM
TH287 hydrochloride_50 uM
RBC8_50 uM
KPT-276_50 uM
Mirin_50 uM
DASA-58_50 uM
Selisistat_50 uM
Isoxanthohumol_50 uM
Grazoprevir_50 uM
FH535_50 uM
SB202190_50 uM
GSK1324726A_50 uM
Anwuligan_50 uM
Alpelisib_50 uM
GNE9605_50 uM
Multi-kinase inhibitor I_50 uM
Erastin_50 uM
KenPaullone_50 uM
IDH1-IN-1_50 uM
Filgotinib_50 uM
UF010_50 uM
NOD-IN-1_50 uM
Brefeldin A_10 uM
Brompheniramine hydrogen ma
AZD1080_50 uM
Isobacachalcone_50 uM
ETC-1002_50 uM
Atractylodin_50 uM
Briciclib_50 uM
PAOA_50 uM
Wnt agonist 1_50 uM
BML-210_50 uM

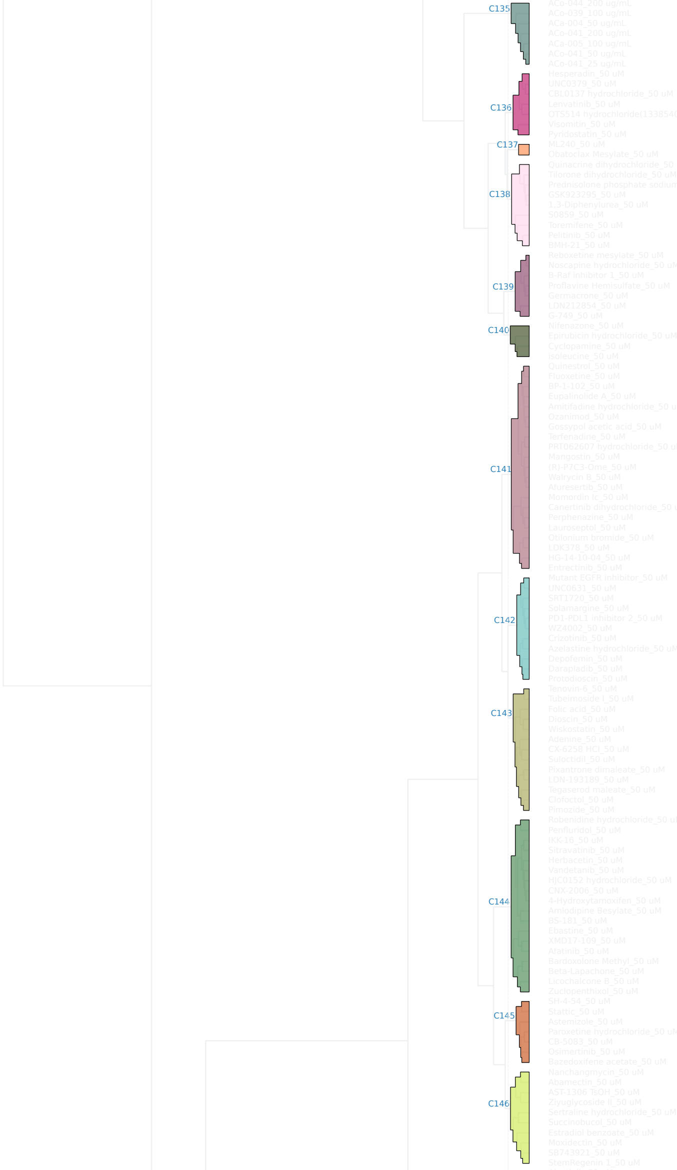
C135
C136
C137
C138
C139
C140
C141
C142
C143
C144
C145
C146
ACo-044_200 ug/mL
ACo-039_100 ug/mL
ACa-004_50 ug/mL
ACo-041_200 ug/mL
ACa-005_100 ug/mL
ACo-041_50 ug/mL
ACo-041_25 ug/mL
Hesperadin_50 uM
UNC0379_50 uM
CBL0137 hydrochloride_50 uM
Lenvatinib_50 uM
OTS514 hydrochloride(133854
Visomitin_50 uM
Pyridostatin_50 uM
ML240_50 uM
Obatoclax Mesylate_50
Quinacrine dihydrochloride_50
Tilorone dihydrochloride_50 uM
Prednisolone phosphate sodium
GSK923295_50 uM
1,3-Diphenylurea_50 uM
S0859_50 uM
Toremifene_50 uM
Pelitinib_50 uM
BMH-21_50 uM
Reboxetine mesylate_50 uM
Noscapine hydrochloride_50 uM
B-Raf inhibitor 1_50 uM
Proflavine Hemisulfate_50 uM
Germacrone_50 uM
LDN212854_50 uM
G-749_50 uM
Nifenazone_50 uM
Epirubicin hydrochloride_50 uM
Cyclopamine_50 uM
isoleucine_50 uM
Quinestrol_50 uM
Fluoxetine_50 uM
BP-1-102_50 uM
Eupalinolide A_50 uM
Amitifadine hydrochloride_50 u
Ozanimod_50 uM
Gossypol acetic acid_50 uM
Terfenadine_50 uM
PRT062607 hydrochloride_50 u
Mangostin_50 uM
(R)-P7C3-Ome_50 uM
Walrycin B_50 uM
Afuresertib_50 uM
Momordin Ic_50 uM
Canertinib dihydrochloride_50 u
Perphenazine_50 uM
Lauroseptol_50 uM
Otilonium bromide_50 uM
LDK378_50 uM
HG-14-10-04_50 uM
Entrectinib_50 uM
Mutant EGFR inhibitor_50 uM
UNC0631_50 uM
SRT1720_50 uM
Solamargine_50 uM
PD1-PDL1 inhibitor 2_50 uM
WZ4002_50 uM
Crizotinib_50 uM
Azelastine hydrochloride_50 uM
Depofemin_50 uM
Darapladib_50 uM
Protodioscin_50 uM
Tenovin-6_50 uM
Tubeimoside I_50 uM
Folic acid_50 uM
Dioscin_50 uM
Wiskostatin_50 uM
Adenine_50 uM
CX-6258 HCl_50 uM
Suloctidil_50 uM
Pixantrone dimaleate_50 uM
LDN-193189_50 uM
Tegaserod maleate_50 uM
Clofoctol_50 uM
Pimozide_50 uM
Robenidine hydrochloride_50 u
Penfluridol_50 uM
IKK-16_50 uM
Sitravatinib_50 uM
Herbacetin_50 uM
Vandetanib_50 uM
HJC0152 hydrochloride_50 uM
CNX-2006_50 uM
4-Hydroxytamoxifen_50 uM
Amlodipine Besylate_50 uM
BS-181_50 uM
Ebastine_50 uM
XMD17-109_50 uM
Afatinib_50 uM
Bardoxolone Methyl_50 uM
Beta-Lapachone_50 uM
Licochalcone B_50 uM
Zuclopenthixol_50 uM
SH-4-54_50 uM
Stattic_50 uM
Astemizole_50 uM
Paroxetine hydrochloride_50 uM
CB-5083_50 uM
Osimertinib_50 uM
Bazedoxifene acetate_50 uM
Nanchangmycin_50 uM
Abamectin_50 uM
AST-1306 TsOH_50 uM
Ziyuglycoside II_50 uM
Sertraline hydrochloride_50 uM
Succinobucol_50 uM
Estradiol benzoate_50 uM
Moxidectin_50 uM
SB743921_50 uM
StemRegenin 1_50 uM

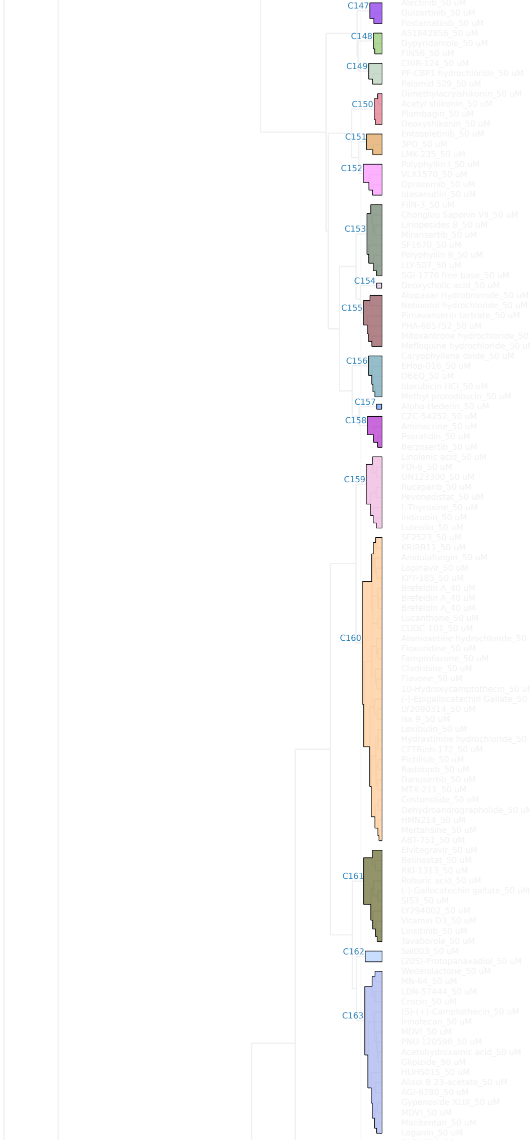
C147
C148
C149
C150
C151
C152
C153
C154
C155
C156
C157
C158
C159
C160
C161
C162
C163
Alectinib_50 uM
Quizartinib_50 uM
Fostamatinib_50 uM
AS1842856_50 uM
Dypyridamole_50 uM
FIN56_50 uM
CHIR-124_50 uM
PF-CBP1 hydrochloride_50 uM
Palomid 529_50 uM
Dimethylacrylshikonin_50 uM
Acetyl shikonin_50 uM
Plumbagin_50 uM
Deoxyshikonin_50 uM
Entospletinib_50 uM
3PO_50 uM
LMK-235_50 uM
Polyphyllin I_50 uM
VLX1570_50 uM
Oprozomib_50 uM
Idasanutlin_50 uM
FIIN-3_50 uM
Chonglou Saponin VII_50 uM
Liriopesides B_50 uM
Miransertib_50 uM
SF1670_50 uM
Polyphyllin B_50 uM
LLY-507_50 uM
SGI-1776 free base_50 uM
Deoxycholic acid_50 uM
Atopaxar Hydrobromide_50 uM
Nebivolol hydrochloride_50 uM
Pimavanserin tartrate_50 uM
PHA-665752_50 uM
Mitoxantrone hydrochloride_50
Mefloquine hydrochloride_50 u
Caryophyllene oxide_50 uM
EHop-016_50 uM
DBEQ_50 uM
Idarubicin HCl_50 uM
Methyl protodioscin_50 uM
Alpha-Hederin_50 uM
CZC-54252_50 uM
Aminacrine_50 uM
Psoralidin_50 uM
Berzosertib_50 uM
Linolenic acid_50 uM
FDI-6_50 uM
ON123300_50 uM
Rucaparib_50 uM
Pevonedistat_50 uM
L-Thyroxine_50 uM
Indirubin_50 uM
Luteolin_50 uM
SF2523_50 uM
KRIBB11_50 uM
Anidulafungin_50 uM
Lopinavir_50 uM
KPT-185_50 uM
Brefeldin A_40 uM
Brefeldin A_40 uM
Brefeldin A_40 uM
Lucanthone_50 uM
CUDC-101_50 uM
Atomoxetine hydrochloride_50
Floxuridine_50 uM
Famprofazone_50 uM
Cladribine_50 uM
Flavone_50 uM
10-Hydroxycamptothecin_50 u
(-)-Epigallocatechin Gallate_50
LY2090314_50 uM
Isx 9_50 uM
Lexibulin_50 uM
Hydrastinine hydrochloride_50
CFTRinh-172_50 uM
Pictilisib_50 uM
Radotinib_50 uM
Danusertib_50 uM
MTX-211_50 uM
Costunolide_50 uM
Dehydroandrographolide_50 u
HMN214_50 uM
Mertansine_50 uM
ABT-751_50 uM
Elvitegravir_50 uM
Belinostat_50 uM
RKI-1313_50 uM
Roburic acid_50 uM
(-)-Gallocatechin gallate_50 uM
SIS3_50 uM
LY294002_50 uM
Vitamin D3_50 uM
Linsitinib_50 uM
Tavaborole_50 uM
Sal003_50 uM
(20S)-Protopanaxadiol_50 uM
Wedelolactone_50 uM
MN-64_50 uM
LDN-57444_50 uM
Crocin_50 uM
(S)-(+)-Camptothecin_50 uM
Irinotecan_50 uM
MDVI_50 uM
PNU-120596_50 uM
Acetohydroxamic acid_50 uM
Glipizide_50 uM
HUHS015_50 uM
Alisol B 23-acetate_50 uM
AGI-6780_50 uM
Gypenoside XLIX_50 uM
MDVI_50 uM
Macitentan_50 uM
Loganin_50 uM

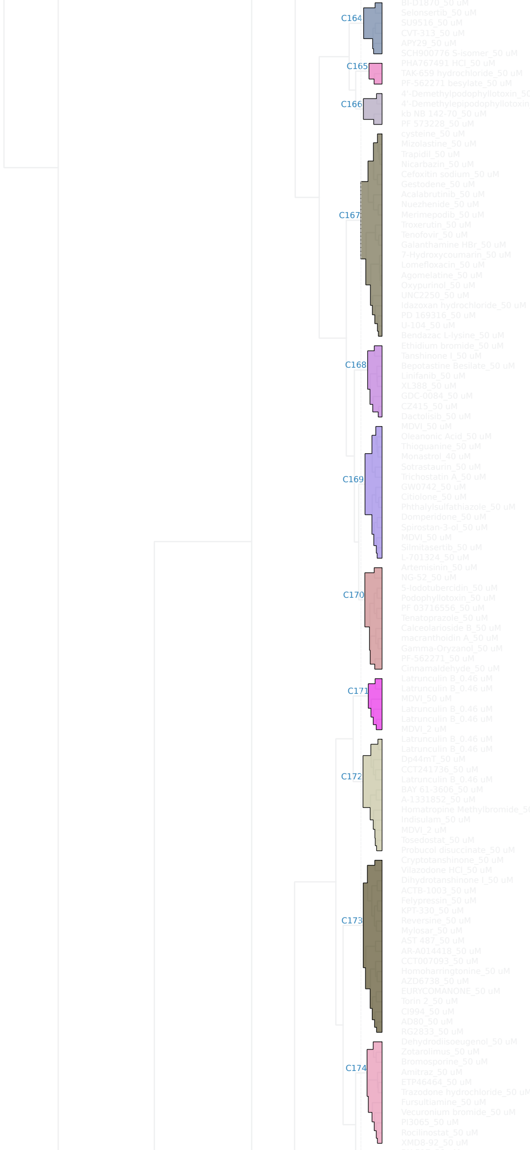
C164
C165
C166
C167
C168
C169
C170
C171
C172
C173
C174
Selonsertib_50 uM
SU9516_50 uM
CVT-313_50 uM
APY29_50 uM
SCH900776 S-isomer_50 uM
PHA767491 HCl_50 uM
TAK-659 hydrochloride_50 uM
PF-562271 besylate_50 uM
4'-Demethylpodophyllotoxin_50
4'-Demethylepipodophyllotoxin
kb NB 142-70_50 uM
PF 573228_50 uM
cysteine_50 uM
Mizolastine_50 uM
Trapidil_50 uM
Nicarbazin_50 uM
Cefoxitin sodium_50 uM
Gestodene_50 uM
Acalabrutinib_50 uM
Nuezhenide_50 uM
Merimepodib_50 uM
Troxerutin_50 uM
Tenofovir_50 uM
Galanthamine HBr_50 uM
7-Hydroxycoumarin_50 uM
Lomefloxacin_50 uM
Agomelatine_50 uM
Oxypurinol_50 uM
UNC2250_50 uM
Idazoxan hydrochloride_50 uM
PD 169316_50 uM
U-104_50 uM
Bendazac L-lysine_50 uM
Ethidium bromide_50 uM
Tanshinone I_50 uM
Bepotastine Besilate_50 uM
Linifanib_50 uM
XL388_50 uM
GDC-0084_50 uM
CZ415_50 uM
Dactolisib_50 uM
MDVI_50 uM
Oleanonic Acid_50 uM
Thioguanine_50 uM
Monastrol_40 uM
Sotrastaurin_50 uM
Trichostatin A_50 uM
GW0742_50 uM
Citiolone_50 uM
Phthalylsulfathiazole_50 uM
Domperidone_50 uM
Spirostan-3-ol_50 uM
MDVI_50 uM
Silmitasertib_50 uM
L-701324_50 uM
Artemisinin_50 uM
NG-52_50 uM
5-Iodotubercidin_50 uM
Podophyllotoxin_50 uM
PF 03716556_50 uM
Tenatoprazole_50 uM
Calceolarioside B_50 uM
macranthoidin A_50 uM
Gamma-Oryzanol_50 uM
PF-562271_50 uM
Cinnamaldehyde_50 uM
Latrunculin B_0.46 uM
Latrunculin B_0.46 uM
MDVI_50 uM
Latrunculin B_0.46 uM
Latrunculin B_0.46 uM
MDVI_2 uM
Latrunculin B_0.46 uM
Latrunculin B_0.46 uM
Dp44mT_50 uM
CCT241736_50 uM
Latrunculin B_0.46 uM
BAY 61-3606_50 uM
A-1331852_50 uM
Homatropine Methylbromide_5
Indisulam_50 uM
MDVI_2 uM
Tosedostat_50 uM
Probucol disuccinate_50 uM
Cryptotanshinone_50 uM
Vilazodone HCl_50 uM
Dihydrotanshinone I_50 uM
ACTB-1003_50 uM
Felypressin_50 uM
KPT-330_50 uM
Reversine_50 uM
Mylosar_50 uM
AST 487_50 uM
AR-A014418_50 uM
CCT007093_50 uM
Homoharringtonine_50 uM
AZD6738_50 uM
EURYCOMANONE_50 uM
Torin 2_50 uM
CI994_50 uM
AD80_50 uM
RG2833_50 uM
Dehydrodiisoeugenol_50 uM
Zotarolimus_50 uM
Bromosporine_50 uM
Amitraz_50 uM
ETP46464_50 uM
Trazodone hydrochloride_50 u
Fursultiamine_50 uM
Vecuronium bromide_50 uM
PI3065_50 uM
Rocilinostat_50 uM
XMD8-92_50 uM

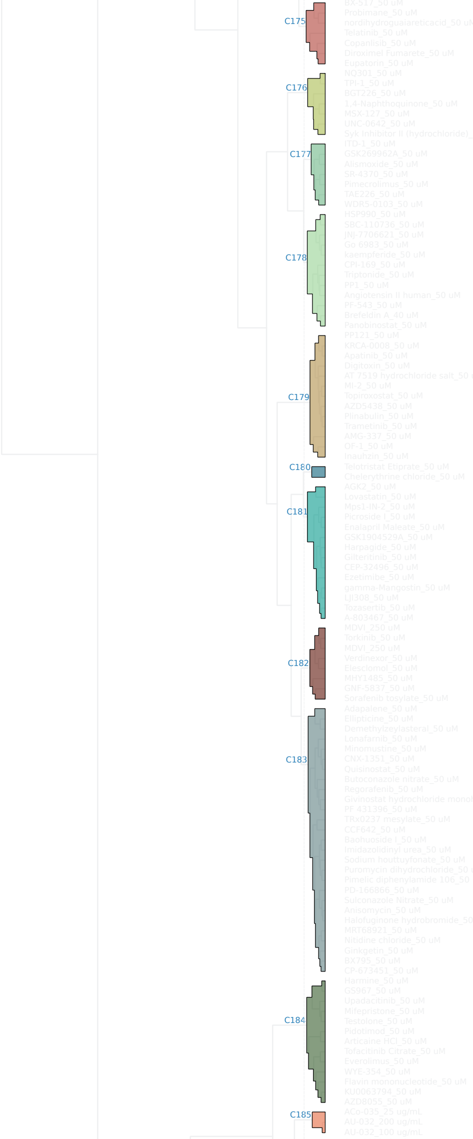

C175
C176
C177
C178
C179
C180
C181
C182
C183
C184
C185
Probimane_50 uM
Telatinib_50 uM
Copanlisib_50 uM
Diroximel Fumarete_50 uM
Eupatorin_50 uM
NQ301_50 uM
TPI-1_50 uM
BGT226_50 uM
1,4-Naphthoquinone_50 uM
MSX-127_50 uM
UNC-0642_50 uM
ITD-1_50 uM
GSK269962A_50 uM
Alismoxide_50 uM
SR-4370_50 uM
Pimecrolimus_50 uM
TAE226_50 uM
WDR5-0103_50 uM
HSP990_50 uM
SBC-110736_50 uM
JNJ-7706621_50 uM
Go 6983_50 uM
kaempferide_50 uM
CPI-169_50 uM
Triptonide_50 uM
PP1_50 uM
Angiotensin II human_50 uM
PF-543_50 uM
Brefeldin A_40 uM
Panobinostat_50 uM
PP121_50 uM
KRCA-0008_50 uM
Apatinib_50 uM
Digitoxin_50 uM
MI-2_50 uM
Topiroxostat_50 uM
AZD5438_50 uM
Plinabulin_50 uM
Trametinib_50 uM
AMG-337_50 uM
OF-1_50 uM
Inauhzin_50 uM
Telotristat Etiprate_50 uM
Chelerythrine chloride_50 uM
AGK2_50 uM
Lovastatin_50 uM
Mps1-IN-2_50 uM
Picroside I_50 uM
Enalapril Maleate_50 uM
GSK1904529A_50 uM
Harpagide_50 uM
Gilteritinib_50 uM
CEP-32496_50 uM
Ezetimibe_50 uM
gamma-Mangostin_50 uM
LJI308_50 uM
Tozasertib_50 uM
A-803467_50 uM
MDVI_250 uM
Torkinib_50 uM
MDVI_250 uM
Verdinexor_50 uM
Elesclomol_50 uM
MHY1485_50 uM
GNF-5837_50 uM
Sorafenib tosylate_50 uM
Adapalene_50 uM
Ellipticine_50 uM
Demethylzeylasteral_50 uM
Lonafarnib_50 uM
Minomustine_50 uM
CNX-1351_50 uM
Quisinostat_50 uM
Butoconazole nitrate_50 uM
Regorafenib_50 uM
PF 431396_50 uM
TRx0237 mesylate_50 uM
CCF642_50 uM
Baohuoside I_50 uM
Imidazolidinyl urea_50 uM
Sodium houttuyfonate_50 uM
PD-166866_50 uM
Sulconazole Nitrate_50 uM
Anisomycin_50 uM
MRT68921_50 uM
Nitidine chloride_50 uM
Ginkgetin_50 uM
BX795_50 uM
CP-673451_50 uM
Harmine_50 uM
GS967_50 uM
Upadacitinib_50 uM
Mifepristone_50 uM
Testolone_50 uM
Pidotimod_50 uM
Articaine HCl_50 uM
Tofacitinib Citrate_50 uM
Everolimus_50 uM
WYE-354_50 uM
Flavin mononucleotide_50 uM
KU0063794_50 uM
AZD8055_50 uM
ACo-035_25 ug/mL
AU-032_200 ug/mL
AU-032_100 ug/mL

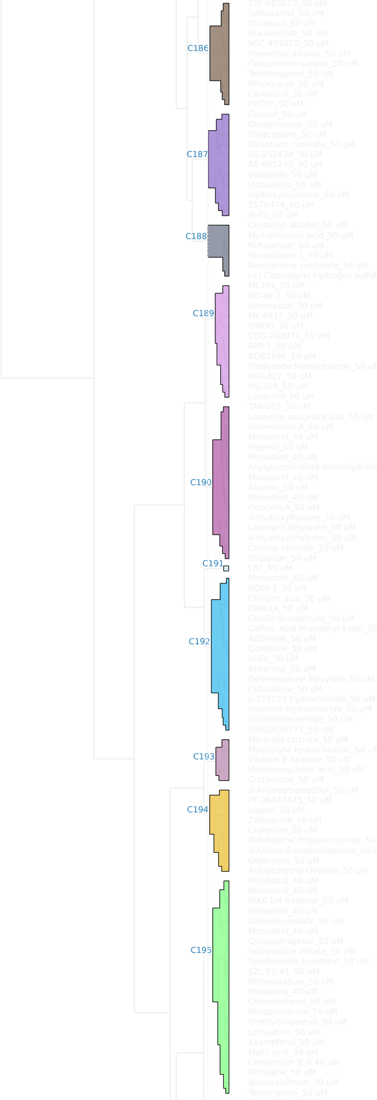
C186
C187
C188
C189
C190
C191
C192
C193
C194
C195
STF-083010_50 uM
Salbutamol_50 uM
Diclazuril_50 uM
Brassinolide_50 uM
NSC 405020_50 uM
Phenethyl alcohol_50 uM
Cefquinome sulfate_50 uM
Tebufenpyrad_50 uM
Ethoxyquin_50 uM
Cadazolid_50 uM
PHTPP_50 uM
Fipronil_50 uM
Drospirenone_50 uM
Entacapone_50 uM
Strontium ranelate_50 uM
AS-252424_50 uM
AS-605240_50 uM
Voxtalisib_50 uM
Vistusertib_50 uM
Hydroxyquinoline_50 uM
ZSTK474_50 uM
JK-P3_50 uM
Cinnamyl alcohol_50 uM
Mycophenolic acid_50 uM
Rufinamide_50 uM
Necrostatin-1_50 uM
Doxylamine succinate_50 uM
(±) Clopidogrel hydrogen sulfat
ML364_50 uM
DO-IN-2_50 uM
Batimastat_50 uM
MK-8617_50 uM
DMOG_50 uM
CCG-203971_50 uM
AMI-1_50 uM
AOB2796_50 uM
Pitofenone hydrochloride_50 uM
PHT-427_50 uM
NS-398_50 uM
Lapachol_50 uM
TAK-063_50 uM
Loxapine succinate salt_50 uM
lavendustin A_50 uM
Monastrol_40 uM
Ingenol_50 uM
Monastrol_40 uM
Allylglucosinolate monohydrate
Monastrol_40 uM
Alizarin_50 uM
Monastrol_40 uM
Oroxylin A_50 uM
3-Hydroxyflavone_50 uM
Lisinopril dihydrate_50 uM
4-Hydroxychalcone_50 uM
Choline chloride_50 uM
Eniporide_50 uM
L67_50 uM
Monastrol_40 uM
NQDI-1_50 uM
Chicoric acid_50 uM
DMXAA_50 uM
Coniferyl aldehyde_50 uM
Caffeic Acid Phenethyl Ester_50
AZD9496_50 uM
Dofetilide_50 uM
Iridin_50 uM
Alibendol_50 uM
Deferoxamine Mesylate_50 uM
Cytarabine_50 uM
L-778123 hydrochloride_50 uM
Harmine hydrochloride_50 uM
Dichlorphenamide_50 uM
GSK-2636771_50 uM
Morantel tartrate_50 uM
Moxisylyte hydrochloride_50 u
Vitamin E Acetate_50 uM
Ursodeoxycholic acid_50 uM
Crotamiton_50 uM
2-Aminoethanethiol_50 uM
PF-06447475_50 uM
Lupeol_50 uM
Zebularine_50 uM
Glafenine_50 uM
Betahistine dihydrochloride_50
2-Amino-6-methylheptane_50 u
Defactinib_50 uM
Acetylcholine chloride_50 uM
Monastrol_40 uM
Monastrol_40 uM
IRAK-1/4 Inhibitor_50 uM
Monastrol_40 uM
Dimenhydrinate_50 uM
Monastrol_40 uM
Cycloastragenol_50 uM
Isoconazole nitrate_50 uM
Turofexorate Isopropyl_50 uM
SZL P1-41_50 uM
Bifendatatum_50 uM
Monastrol_40 uM
Chlorobutanol_50 uM
Phosphoserine_50 uM
Diethylstilbestrol_50 uM
Lomustine_50 uM
Kaempferol_50 uM
Malic acid_50 uM
Latrunculin B_0.46 uM
Bimolane_50 uM
glucosylvitexin_50 uM
Tectorigenin_50 uM

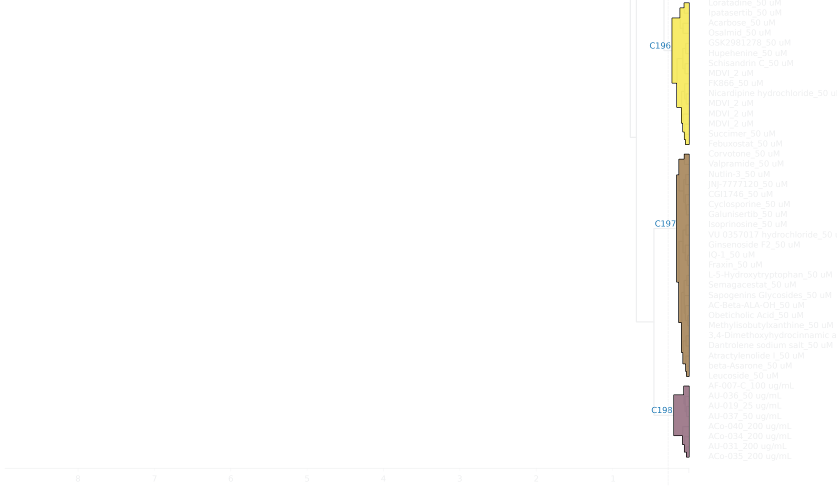

C196
C197
C198
Loratadine_50 uM
Ipatasertib_50 uM
Acarbose_50 uM
Osalmid_50 uM
GSK2981278_50 uM
Hupehenine_50 uM
Schisandrin C_50 uM
MDVI_2 uM
FK866_50 uM
Nicardipine hydrochloride_50 u
MDVI_2 uM
MDVI_2 uM
MDVI_2 uM
Succimer_50 uM
Febuxostat_50 uM
Corvotone_50 uM
Valpramide_50 uM
Nutlin-3_50 uM
JNJ-7777120_50 uM
CGI1746_50 uM
Cyclosporine_50 uM
Galunisertib_50 uM
Isoprinosine_50 uM
VU 0357017 hydrochloride_50 u
Ginsenoside F2_50 uM
IQ-1_50 uM
Fraxin_50 uM
L-5-Hydroxytryptophan_50 uM
Semagacestat_50 uM
Sapogenins Glycosides_50 uM
AC-Beta-ALA-OH_50 uM
Obeticholic Acid_50 uM
Methylisobutylxanthine_50 uM
3,4-Dimethoxyhydrocinnamic a
Dantrolene sodium salt_50 uM
Atractylenolide I_50 uM
beta-Asarone_50 uM
Leucoside_50 uM
AF-007-C_100 ug/mL
AU-036_50 ug/mL
AU-019_25 ug/mL
AU-037_50 ug/mL
ACo-040_200 ug/mL
ACo-034_200 ug/mL
AU-031_200 ug/mL
ACo-035_200 ug/mL
8
7
6
5
4
3
2
1

Figure S1. Full hierarchical clusters of *Aconitum* samples, control compounds, and members of the TargetMol library. Cluster 93 shows positive control latrunculin B as a frame of reference for establishing clustering thresholds.

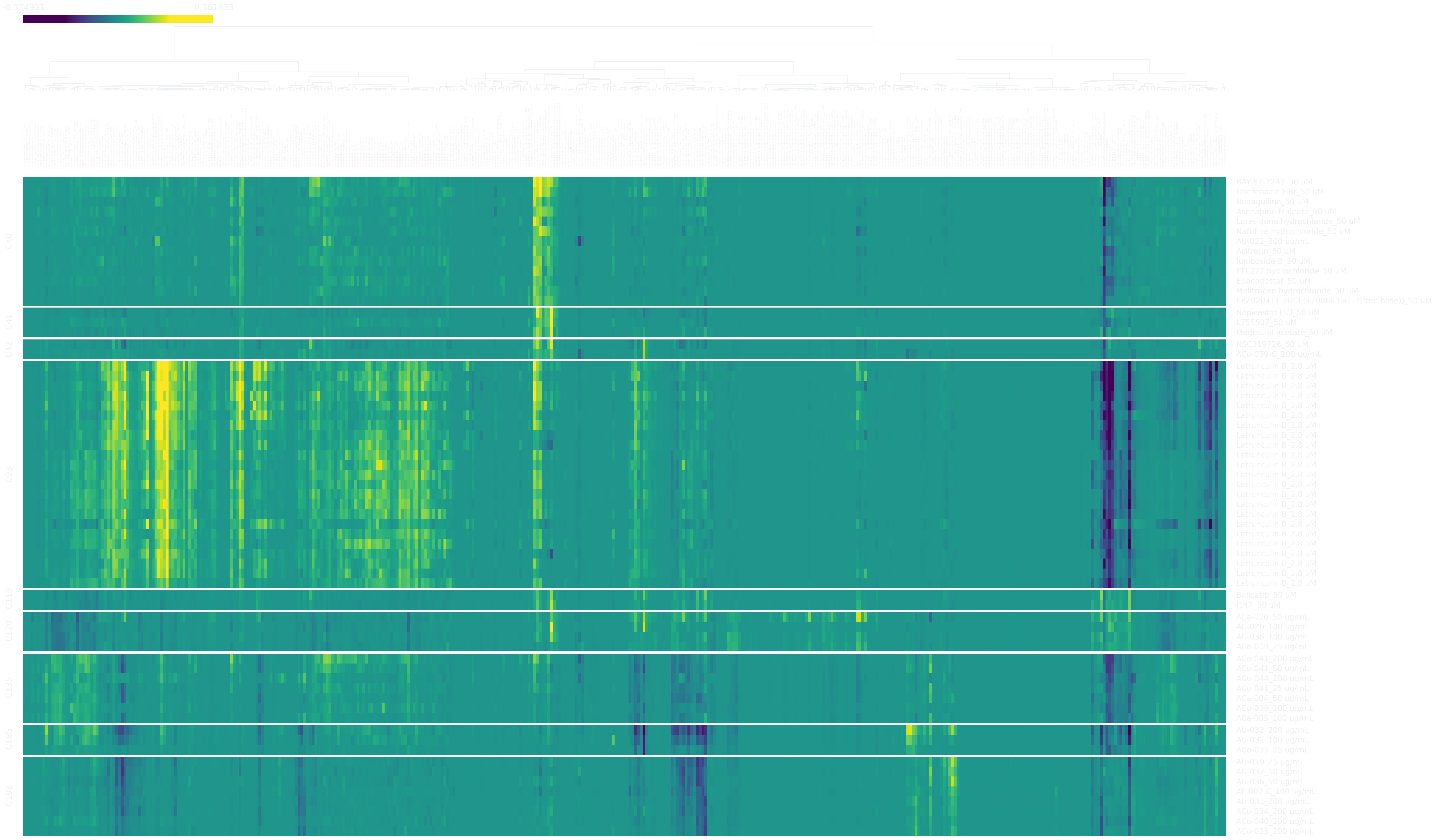
-0.374931
0.361833
C40
C41
C42
C93
C119
C120
C135
C185
C198
BAY 87-2243_50 uM
Darifenacin HBr_50 uM
Bedaquiline_50 uM
Asenapine Maleate_50 uM
Lurasidone hydrochloride_50 uM
Naftifine hydrochloride_50 uM
AU-022_200 ug/mL
Acitretin_50 uM
Jujuboside B_50 uM
FTI 277 hydrochloride_50 uM
Epacadostat_50 uM
Melitracen hydrochloride_50 uM
EPZ020411 2HCl (1700663-41-7(free base))_50 uM
Nepicastat HCl_50 uM
L755507_50 uM
Megestrol acetate_50 uM
NSC319726_50 uM
ACo-059-C_200 ug/mL
Latrunculin B_2.8 uM
Latrunculin B_2.8 uM
Latrunculin B_2.8 uM
Latrunculin B_2.8 uM
Latrunculin B_2.8 uM
Latrunculin B_2.8 uM
Latrunculin B_2.8 uM
Latrunculin B_2.8 uM
Latrunculin B_2.8 uM
Latrunculin B_2.8 uM
Latrunculin B_2.8 uM
Latrunculin B_2.8 uM
Latrunculin B_2.8 uM
Latrunculin B_2.8 uM
Latrunculin B_2.8 uM
Latrunculin B_2.8 uM
Latrunculin B_2.8 uM
Latrunculin B_2.8 uM
Latrunculin B_2.8 uM
Latrunculin B_2.8 uM
Latrunculin B_2.8 uM
Latrunculin B_2.8 uM
Latrunculin B_2.8 uM
Baricitinib_50 uM
J147_50 uM
ACa-020_50 ug/mL
AU-020_100 ug/mL
AU-036_100 ug/mL
ACo-009_25 ug/mL
ACo-041_200 ug/mL
ACo-041_50 ug/mL
ACo-044_200 ug/mL
ACo-041_25 ug/mL
ACa-004_50 ug/mL
ACo-039_100 ug/mL
ACa-005_100 ug/mL
AU-032_200 ug/mL
AU-032_100 ug/mL
ACo-035_25 ug/mL
AU-019_25 ug/mL
AU-037_50 ug/mL
AU-036_50 ug/mL
AF-007-C_100 ug/mL
AU-031_200 ug/mL
ACo-034_200 ug/mL
ACo-040_200 ug/mL
ACo-035_200 ug/mL

Figure S2. Cell Painting heatmap of select *Aconitum*, control, and TargetMol library clusters. Vertical axis shows Cell Painting profiles for all clusters containing *Aconitum* samples and positive control latrunculin B. Horizontal axis shows hierarchical clustering of a reduced subset of 429 features. Yellow shading indicates increasing HistDiff values compared to vehicle control while deep blue shading indicates decreasing HistDiff values compared to vehicle control.

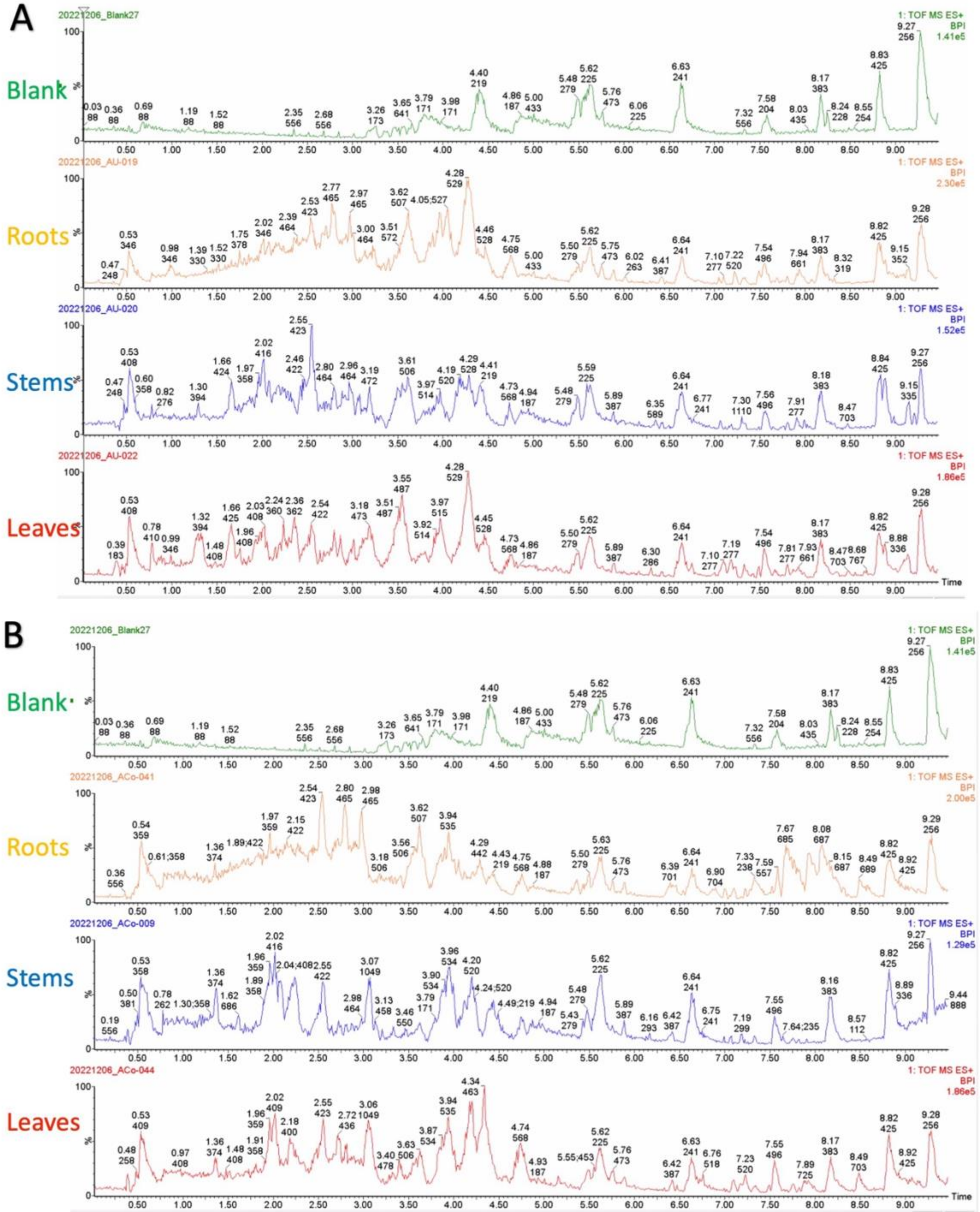

Figure S3. Chromatograms of different parts from American *Aconitum*. A. *Aconitum uncinatum*; B. *Aconitum columbianum.*

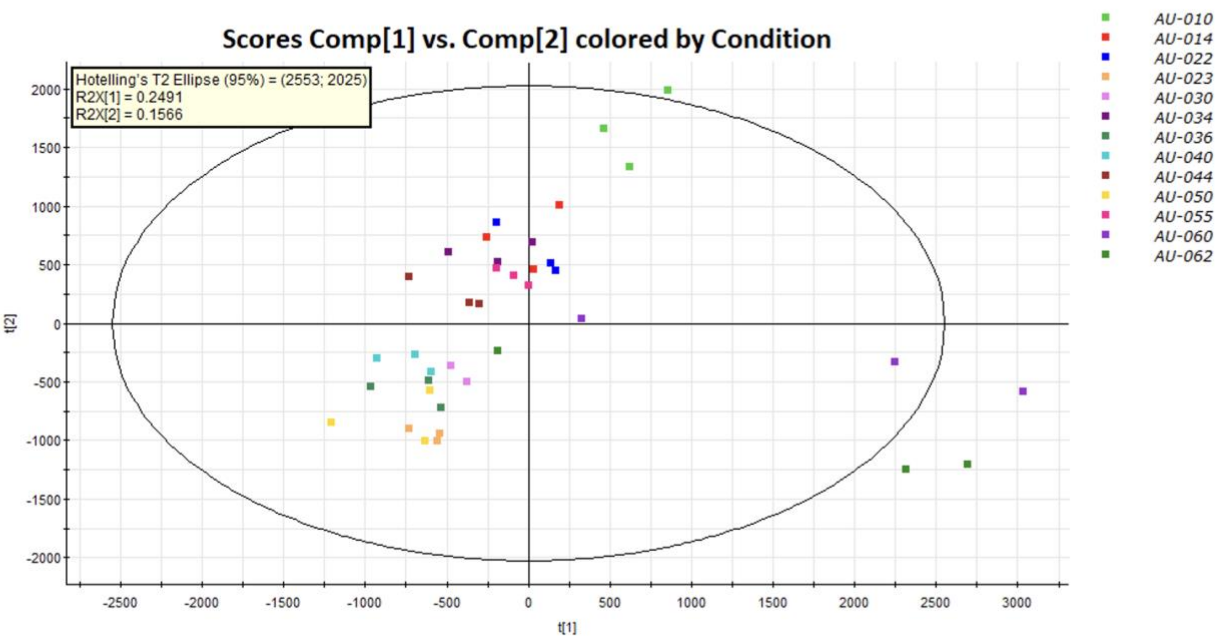

Figure S4. Principal component analysis of thirteen *Aconitum uncinatum* leaf samples. The dark blue squares sample AU-022, and other colored squares from twelve other *Aconitum uncinatum* leaf samples.

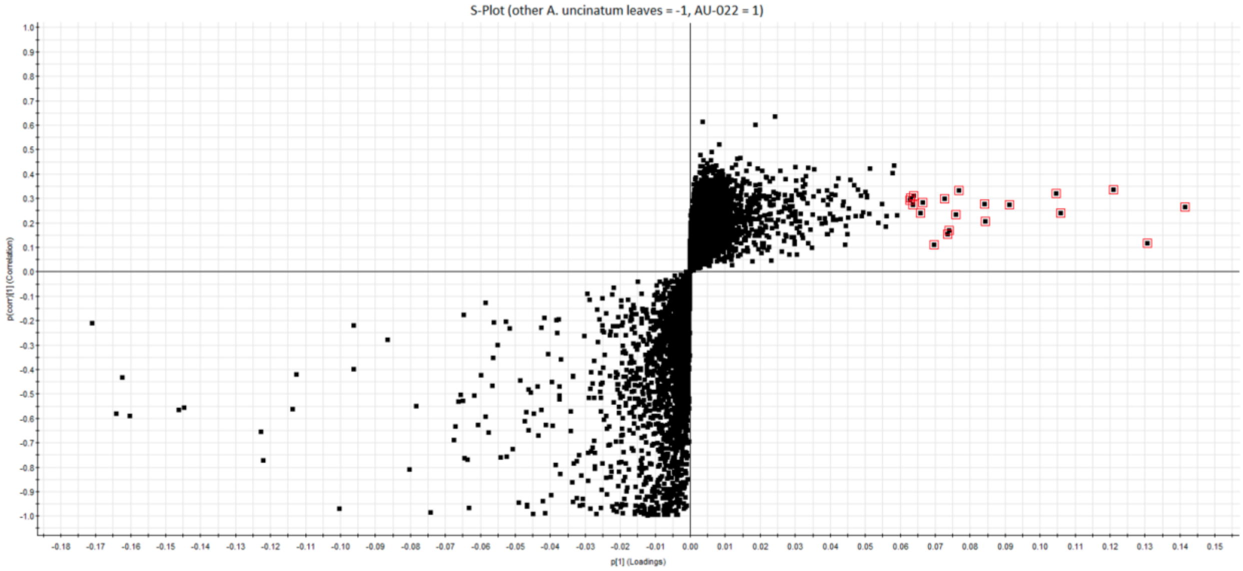

Figure S5. S-plot of *Aconitum uncinatum* leaf samples. The upper right side shows the chemical features from AU-022, and bottom left shows the chemical features from other leaf samples of *Aconitum uncinatum*.
